# Dual Checkpoint Inhibition With or Without Chemotherapy in Metastatic NSCLC: A Reconstructed Individual Patient Data Meta-Analysis

**DOI:** 10.1016/j.jtocrr.2026.101059

**Published:** 2026-08-07

**Authors:** Skyler Taylor, Isadora Mamede, Gabriela Gazzoni, Israt Jahan Riya, Ifrat Jahan Piya, Carlos Stecca, Guilherme Lopes, Antonious Hazim, Vinicius Ernani

**Affiliations:** a Division of Hematology & Medical Oncology, Mayo Clinic Arizona, Phoenix, Arizona; b Faculty of Medicine, Federal University of São João del Rei, Divinópolis, Brazil; c Institute of Medical Assistance to the State Public Servant, São Paulo, Brazil; d Faculty of Medicine, Dhaka Medical College and Hospital, Dhaka, Bangladesh; e Department of Medical Oncology, BC Cancer – Abbotsford, Abbotsford, BC, Canada; f Faculty of Medicine, Centro Universitário Faculdade de Medicina do ABC, Santo André, Brazil

**Keywords:** Immune checkpoint inhibitors, Systematic review, Advanced non–small cell lung cancer, Chemotherapy

## Abstract

**Introduction:**

Dual immune checkpoint inhibition (DICI) is an established first-line treatment for metastatic NSCLC. Whether the addition of chemotherapy provides an incremental survival benefit beyond DICI alone remains unclear.

**Methods:**

We performed a systematic review and reconstructed individual patient data meta-analysis of trials evaluating first-line DICI and DICI plus chemotherapy (DICI-CT) in metastatic NSCLC. Data were reconstructed from Kaplan–Meier curves. Overall survival (OS) and progression-free survival were analyzed using Cox proportional hazards and time-varying models. Prespecified subgroup analyses were conducted according to PD-L1 expression, histology, and STK11 or KEAP1 mutational status.

**Results:**

Four trials comprising 1654 patients were included (699 receiving DICI-CT and 955 receiving DICI). Median OS was similar between the groups (15.4 versus 15.0 mo, hazard ratio = 0.93, 95% confidence interval: 0.81–1.08). Proportional hazards assumptions were violated, and time-varying analyses suggested modest late survival differences favoring DICI, with higher 5-year OS (19.4% versus 17.1%) and progression-free survival (10.5% versus 8.2%). In STK11-mutated tumors, DICI-CT was associated with a limited early survival benefit that was not sustained over time. No clear differences were observed in KEAP1-mutated tumors.

**Conclusions:**

The addition of chemotherapy to DICI was not associated with improved survival in an unselected population of patients with metastatic NSCLC. These findings support an individualized approach to the use of chemotherapy in combination with DICI.

## Introduction

Metastatic NSCLC has undergone a major therapeutic transformation with the incorporation of immunotherapy into routine clinical practice.^1^ For patients without actionable driver mutations, current first-line treatment options include programmed cell death protein 1 (PD-1) or programmed death-ligand 1 (PD-L1) inhibitors as monotherapy, dual immune checkpoint inhibition (DICI) combining PD-1 or PD-L1 with cytotoxic T-lymphocyte–associated protein 4 (CTLA-4) inhibition, chemotherapy combined with PD-1 or PD-L1 inhibitors, or regimens combining chemotherapy with DICI.^2^

Multiple phase III trials have demonstrated the efficacy of DICI and DICI plus chemotherapy (DICI-CT), leading to their incorporation into clinical guidelines,^3^, ^4^, ^5^ with approved regimens including nivolumab plus ipilimumab with or without chemotherapy and durvalumab plus tremelimumab combined with chemotherapy. In the United States, nivolumab plus ipilimumab is not approved for PD-L1 expression less than 1%.^2^^,^^6^^,^^7^ The biological rationale for adding chemotherapy to dual checkpoint blockade lies in its immunomodulatory effects, including the induction of immunogenic cell death, enhanced tumor antigen release and cross-presentation, and the depletion of immunosuppressive cellular populations.^8^^,^^9^

DICI may be particularly beneficial in patient subgroups with limited responses to single-agent immunotherapy, including tumors with low or absent PD-L1 expression. The CheckMate 9LA trial demonstrated that the addition of short-course chemotherapy to nivolumab plus ipilimumab significantly improved overall survival (OS) compared with chemotherapy alone across PD-L1 strata and histologic subtypes.^3^^,^^10^ Similarly, the POSEIDON trial revealed improved OS with durvalumab plus tremelimumab combined with chemotherapy.^5^

However, no randomized trial or previous meta-analysis has directly compared DICI with DICI-CT. This distinction is clinically relevant because it informs whether chemotherapy provides incremental benefit beyond dual immunotherapy or whether selected patients may achieve comparable outcomes while avoiding chemotherapy-related toxicities. The objective of this reconstructed individual patient-level meta-analysis was to compare the efficacy of DICI with or without chemotherapy across available phase III randomized trials in the first-line treatment of metastatic NSCLC.

## Materials and Methods

### Search Strategy and Selection Criteria

This systematic review and meta-analysis was conducted in accordance with the Preferred Reporting Items for Systematic Reviews and Meta-Analyses guidelines (Supplementary Data 1, Supplementary Table 1).^11^ The study protocol was registered with PROSPERO (CRD420251230272). A systematic search of PubMed, Embase, the Cochrane Central Register of Controlled Trials, and ClinicalTrials.gov was performed to identify randomized controlled trials (RCTs) published up to November 10, 2025. Additional sources included conference proceedings from the American Society of Clinical Oncology, the European Society for Medical Oncology, the World Conference on Lung Cancer, and the American Association for Cancer Research. The reference lists of relevant systematic reviews were also screened. The complete search strategy is provided in the appendix (Supplementary Data 1, Supplementary Methods 1). Two investigators (G.G. and I.M.) independently screened the studies, with discrepancies resolved by consensus.

Eligible studies were RCTs or exploratory analyses of RCTs enrolling patients with treatment-naive advanced NSCLC and comparing a PD-1 or PD-L1 inhibitor plus a CTLA-4 inhibitor, with or without chemotherapy, versus platinum-based chemotherapy. Additional inclusion criteria were the availability of survival outcomes with long-term follow-up (a minimum follow-up of 5 y) or subgroup analyses stratified by KRAS, STK11, or KEAP1 mutational status, as well as the inclusion of Kaplan–Meier (KM) survival curves. Studies were excluded if they had overlapping patient populations or lacked a control arm. No restrictions were applied regarding language or publication date. When multiple reports of a trial existed, the most recent or most complete publication was used.

As this study is a systematic review and meta-analysis based exclusively on previously published aggregate data, informed consent from individual participants was not required. All included studies were conducted in accordance with the ethical standards of their respective institutions.

### Data Synthesis and Statistical Analysis

Two independent reviewers (G.G. and I.M.) extracted baseline characteristics and outcome data from all eligible studies. The extracted baseline variables included study identifiers, author and publication information, study design, treatment arms, sample size, mean or median age, sex distribution, histology, PD-L1 tumor proportion score (TPS), number of mutation-assessable patients, prevalence of KRAS, STK11, and KEAP1 mutations, and follow-up duration.

Time-to-event outcomes were extracted from published KM curves. Curves were digitized using WebPlotDigitizer (version 4), and individual patient data (IPD) were reconstructed using the IPD from KM algorithm.^12^ Three independent authors (I.M., I.J.P., and I.J.R.) performed the extraction of KM coordinates, numbers at risk, and OS and progression-free survival (PFS) curves for the overall population and available subgroups (histology, PD-L1 TPS, KRAS, STK11, KEAP1).

Reconstructed KM curves were compared with the original publications. Extraction was repeated whenever predefined discrepancies were observed: more than or equal to 1-patient difference in the number at risk at time 0, more than 0.3-month difference in the reported median OS, or more than or equal to 2% difference in 5-year OS. This quality-control process was implemented to improve the accuracy and reproducibility of IPD reconstruction.

The primary end point was OS comparing DICI versus DICI-CT in the overall population. A secondary end point was PFS in the overall population. We performed subgroup analyses for the OS end point defined by PD-L1 TPS, tumor histology, and STK11 or KEAP1 mutational status. A prespecified subgroup analysis of OS according to KRAS mutation status could not be performed because subgroup-specific KM curves were unavailable for the DICI arm. OS was defined as the interval from randomization to death from any cause. PFS was defined as the interval from randomization to disease progression or death from any cause.

All analyses were performed in R version 4.5.0 using the *survival*, *coxme*, *survminer*, *broom*, and *survRM2* packages. To enable an indirect treatment comparison anchored to a common reference, the chemotherapy-alone (CT) arm from each included trial was reconstructed from published KM curves using procedures identical to those applied to the primary treatment arms. Because no trial directly randomized patients between DICI and DICI-CT, all comparative estimates constitute indirect treatment comparisons. The primary analysis used a mixed-effects Cox proportional hazards (PH) model with a random intercept by trial to account for between-trial heterogeneity in the baseline hazard. CT was the reference category. Fixed effects for DICI and DICI-CT versus CT were estimated, and the indirect DICI versus DICI-CT contrast was derived as the difference between the two log-hazard ratios, with variance propagated from the full variance–covariance matrix, including the covariance term. Survival distributions were summarized using KM curves. Median follow-up was estimated using the reverse KM method. The PH assumption was evaluated using scaled Schoenfeld residuals, graphical inspection of residual-versus-time plots, and global PH tests from the cox.zph function. When the PH assumption was violated for the treatment effect, a time-varying coefficient Cox model was fitted using a log-time interaction.

$$h\left(t\right)=h_{0}\left(t\right)\exp\left\{\beta_{0}\cdot treat+\beta_{1}\cdot treat\times \log\left(t+1\right)\right\}\text{,}$$

allowing estimation of a time-dependent hazard ratio, HR(t). HR(t) curves with 95% confidence intervals were generated in the observed follow-up period.

Because non-PH and time-varying treatment effects were observed in individual trials, such as MYSTIC, a reconstructed IPD meta-analysis was preferred over aggregate-data approaches that assume PH. Survival outcomes in the chemotherapy control arms of the individual trials were within the range typically reported for first-line platinum-doublet chemotherapy in metastatic NSCLC, supporting the general comparability of the trial populations, although residual cross-trial confounding cannot be excluded. Restricted mean survival time (RMST) analyses were also performed using the rmst2 function.^13^ RMST was computed up to a prespecified truncation time of τ = 60 months or 24 months. Several sensitivity analyses were performed to assess the robustness of the study. A random-effects network meta-analysis was performed using published HRs from the original trial reports (R package netmeta). As no closed loop was present, formal inconsistency testing was not applicable.

Subgroup interaction tests used likelihood ratio tests comparing coxme models with and without the treatment-by-subgroup interaction term (1-degree-of-freedom Wald contrast for indirect DICI versus DICI-CT). Baseline characteristics of the pooled DICI and DICI-CT cohorts were compared using standardized mean differences (SMDs) and chi-square or Welch *t* tests as appropriate. An SMD exceeding 0.10 was considered indicative of a potentially meaningful imbalance.

### Quality Assessment and Publication Bias

Risk of bias was assessed independently by two reviewers (G.L. and I.M.) using the Cochrane Risk of Bias 2 (RoB-2) tool.^14^ Publication bias for OS and PFS outcomes was not tested because fewer than 10 studies were available, in accordance with Cochrane guidance.

## Results

A total of 3227 citations were identified, of which 2450 remained after duplicate removal. Furthermore, 101 records underwent full-text screening, and, ultimately, eight reports from four randomized trials met the inclusion criteria (Fig. 1).^3^, ^4^, ^5^^,^^10^^,^^15^, ^16^, ^17^, ^18^ All eligible studies reported OS outcomes. Among the 1654 included patients, 955 (57.8%) received DICI and 699 (42.3%) received DICI-CT (Table 1). Nonsquamous histology was present in 1180 patients (71.3%), 1109 (67.0%) had a PD-L1 expression more than or equal to 1%, 121 had KEAP1-mutated tumors, and 187 had STK11-mutated tumors. Detailed baseline characteristics for each study and by arm included in this analysis are provided in Supplementary Data 1, Supplementary Tables 2 and 3. Across trials, baseline characteristics were broadly balanced. Median age ranged from 63 to 65 years, the proportion of current or former smokers was approximately 83% to 87%, and Eastern Cooperative Oncology Group performance status 1 was present in 62% to 68% of patients. The DICI-CT arm had a higher proportion of squamous histology (34.2% versus 28.3%; SMD = 0.21; *p* = 0.01) and a lower proportion of PD-L1 more than or equal to 1% expression (59.7% versus 72.5%; SMD = 0.21; *p* < 0.001) compared with the DICI arm. KEAP1 wild-type prevalence was higher in the DICI arm (64.8% versus 49.9%; SMD = 0.30; *p* < 0.001). These imbalances reflect the enrollment composition of CheckMate 9LA and POSEIDON and should be considered when interpreting pooled results.

**Figure 1:**
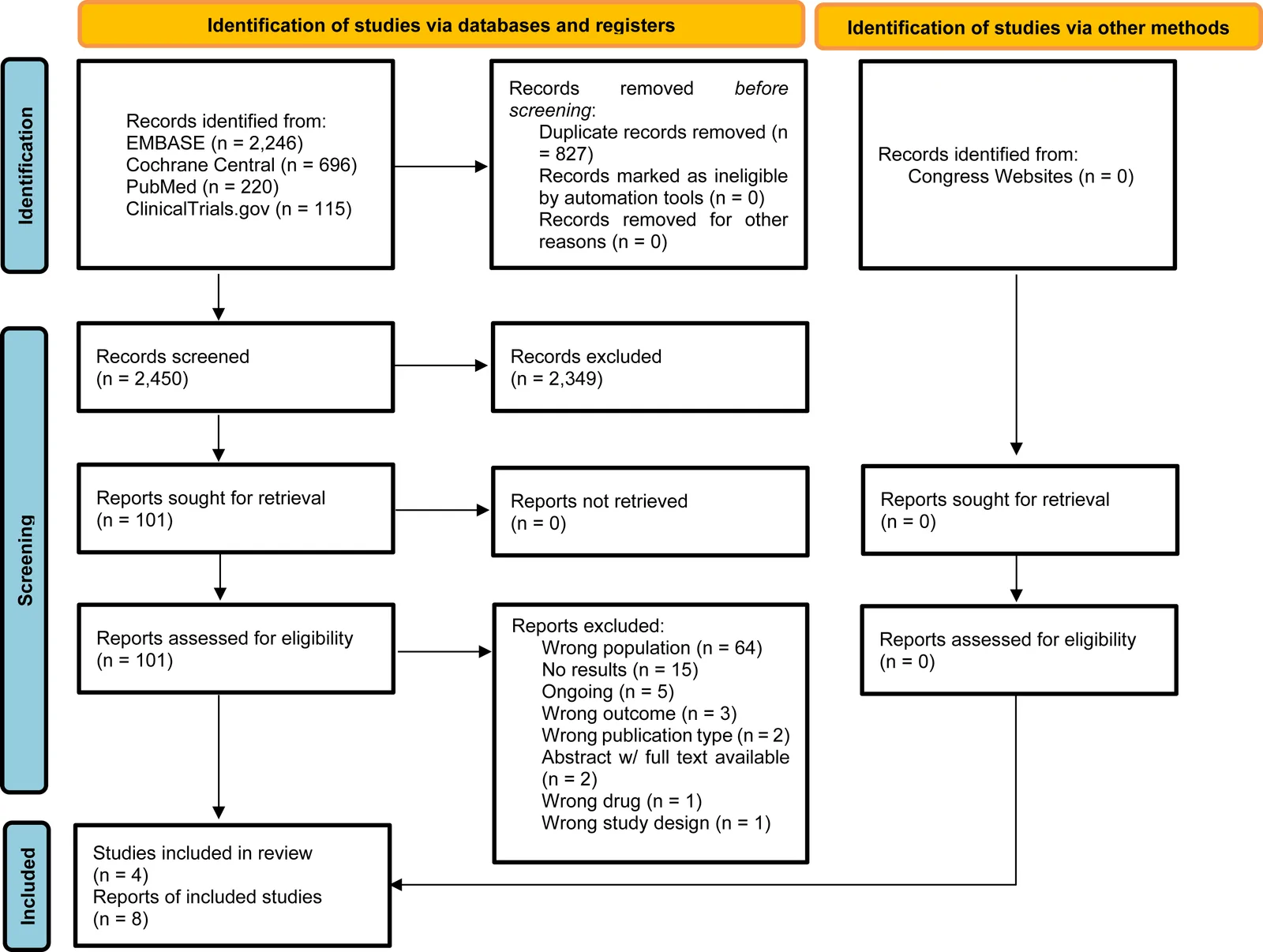
PRISMA flowchart of included studies. PRISMA, Preferred Reporting Items for Systematic Reviews and Meta-Analyses.

**Table 1:** Study Characteristics and Available Analyses of Included Trials

Trial	CheckMate 227 Part 1^4^^,^^16^^,^^17^	CheckMate 9LA^3^^,^^10^	MYSTIC^15^^,^^18^	POSEIDON^5^
Arm included in the analysis	Group 1: nivolumab + ipilimumab (PD-L1 ≥ 1%) Group 2: nivolumab + ipilimumab (PD-L1 < 1%)	Nivolumab + ipilimumab + chemotherapy	Durvalumab + tremelimumab	Durvalumab + tremelimumab + chemotherapy
No. of patients included	Group 1: 396 Group 2: 187	361	372	338
ICI class	Anti–CTLA-4 + anti–PD-1	Anti–CTLA-4 + anti–PD-1	Anti–CTLA-4 + anti–PD-L1	Anti–CTLA-4 + anti–PD-L1
Primary end point	OS (PD-L1 ≥ 1%)	OS	OS and PFS (PD-L1 ≥ 25%)	OS and PFS
Available outcomes	OS; PFS; PD-L1 ≥ and < 1%; SQ and nonSQ	OS; PFS; PD-L1 ≥ and < 1%; SQ and nonSQ; STK11; KRAS	OS; PFS (PD-L1 ≥ 25%); PD-L1 ≥ 25%; KEAP1; STK11	OS; PFS; PD-L1 ≥ and < 1%; SQ and nonSQ; KEAP1; STK11; KRAS

ICI, immune checkpoint inhibition; NonSQ, nonsquamous histology; OS, overall survival; PFS, progression-free survival; SQ, squamous histology.

The median follow-up of the overall cohort was 65.5 months (95% CI: 65.5–67.7). Among censored patients, the median follow-up was 60.5 months (interquartile range = 29.8–66.5) for DICI and 67.5 months (interquartile range = 61.5–73.4) for DICI-CT. The median follow-up in each analysis is available in Supplementary Data 1, Supplementary Table 4.

Reconstructed KM curves for all studies are found in Supplementary Data 1, Supplementary Figure 1; study-level data use is summarized in Supplementary Data 1, Supplementary Table 5. Original and reconstructed survival estimates are compared in Supplementary Data 1, Supplementary Tables 6 and 7.

The median OS was similar between treatment groups (DICI-CT: 15.4 mo [95% CI: 13.7–16.9] versus DICI: 15.0 mo [95% CI: 13.2–16.8]), with no significant difference in the mixed-effects Cox model (HR = 0.93, 95% CI: 0.81–1.08, *p* = 0.33; Fig. 2*A*). The aggregate-data network meta-analysis sensitivity analysis was consistent (DICI versus DICI-CT: HR = 0.95, 95% CI: 0.75–1.21, *p* = 0.667; Supplementary Tables 8). Because the PH assumption was violated (Supplementary Data 1, Supplementary Fig. 2*A*; Supplementary Table 9), a time-varying coefficient Cox model was applied. This analysis suggested modest late survival differences favoring DICI, with HR(t) crossing above 1 after the initial treatment period (Supplementary Data 1, Supplementary Fig. 3*A*). Consistently, 5-year OS was higher with DICI (19.4%, 95% CI: 16.8%–22.4%) than with DICI-CT (17.1%, 95% CI: 14.5%–20.2%). RMST differences at 60 months were not significant (Supplementary Data 1, Supplementary Fig. 5*A*).

**Figure 2:**
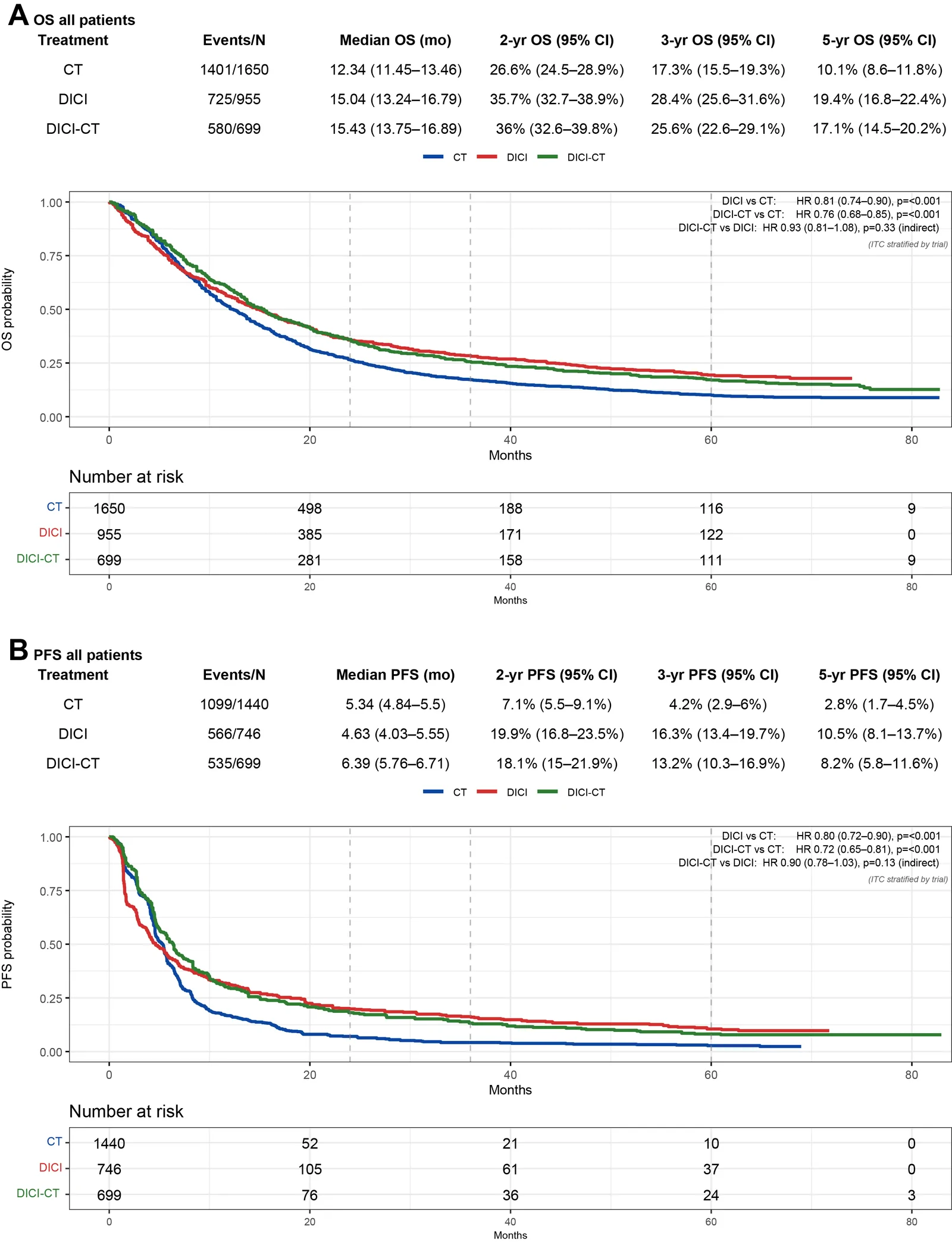
(*A*) OS and (*B*) PFS with DICI and DICI-CT. DICI, dual immune checkpoint inhibition; DICI-CT, dual immune checkpoint inhibition plus chemotherapy; OS, overall survival; PFS, progression-free survival.

No significant difference in PFS was observed in the mixed-effect analysis (Fig. 2*B*). However, due to a PH violation (Supplementary Data 1, Supplementary Fig. 2*B*; Supplementary Table 9), the time-varying model suggested delayed differences favoring DICI after 12 months (Supplementary Data 1, Supplementary Fig. 3*B*).

In PD-L1 more than or equal to 1%, median OS did not differ significantly (DICI-CT: 15.8 mo versus DICI: 16.6 mo; HR = 0.94, 95% CI: 0.78–1.12, *p* = 0.49; Fig. 3*A*). With PH violation (Supplementary Data 1, Supplementary Fig. 2*C*; Supplementary Table 9), time-varying modeling suggested delayed survival separation favoring DICI (Supplementary Data 1, Supplementary Fig. 3*C*). The 5-year OS favored DICI (22.8% versus 19.6%). RMST differences at 60 months were nonsignificant (Supplementary Data 1, Supplementary Fig. 5*C*). In PD-L1 less than 1%, no OS difference was observed between the treatment groups (Fig. 3*B*).

**Figure 3:**
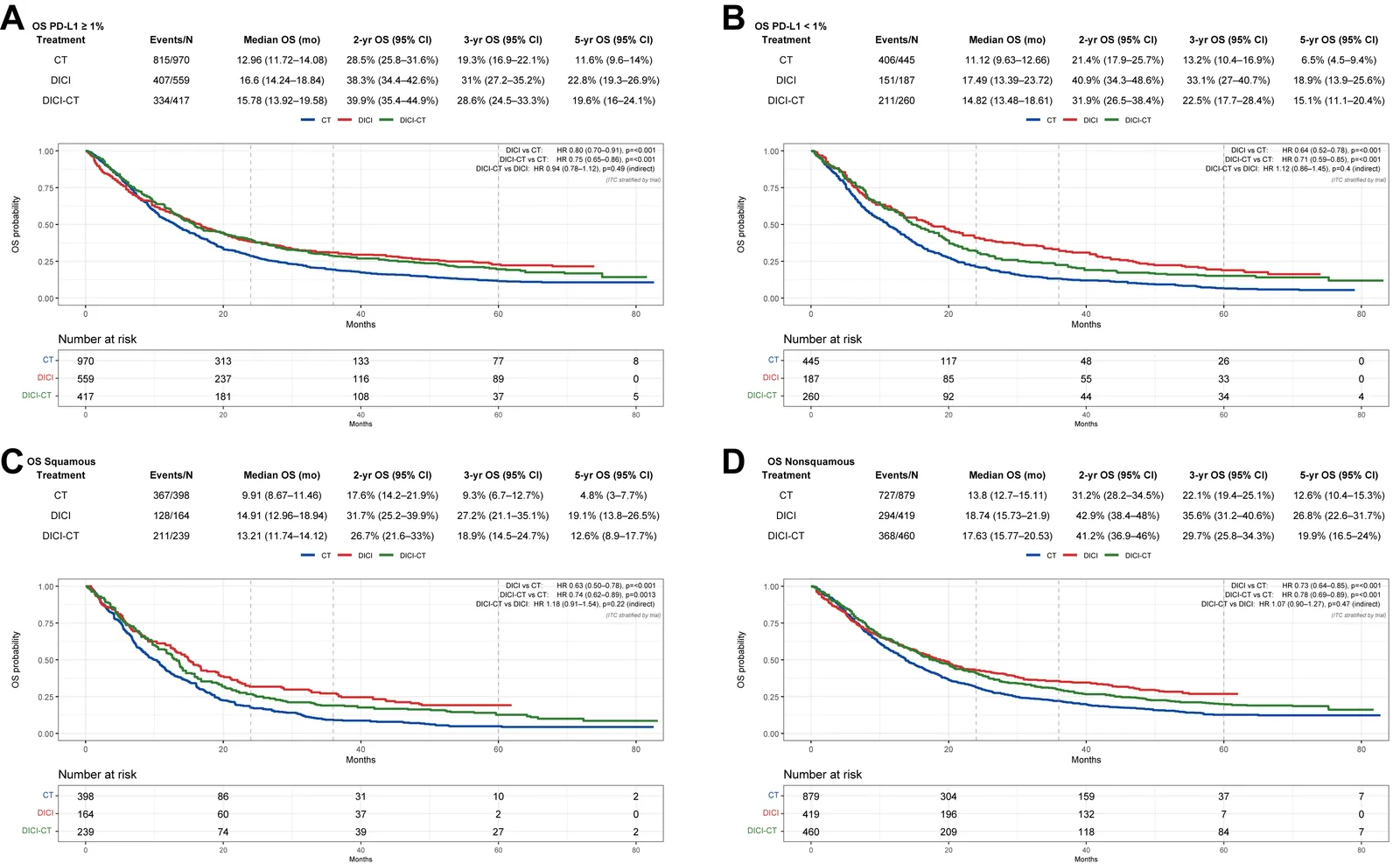
OS with DICI and DICI-CT in (*A*) PD-L1 more than or equal to 1%, (*B*) PD-L1 less than 1%, (*C*) squamous, and (*D*) nonsquamous patients. DICI, dual immune checkpoint inhibition; DICI-CT, dual immune checkpoint inhibition plus chemotherapy.

When stratified by histology, among squamous NSCLC, OS did not differ (HR = 1.18, 95% CI: 0.91–1.54, *p* = 0.22; Fig. 3*C*). In nonsquamous disease, no difference was detected in the indirect comparison (HR = 1.07, 95% CI: 0.90–1.27, *p* = 0.47; Fig. 3*D*), but a time-varying analysis suggested delayed survival differences favoring DICI beyond 24 months (Supplementary Data 1, Supplementary Fig. 3*D*).

Among patients with KEAP1-mutated tumors (*n* = 85), no significant difference in OS was observed (HR = 0.64, 95% CI: 0.36–1.14, *p* = 0.13; Fig. 4*A*). In patients with KEAP1 wild-type tumors (*n* = 562), DICI-CT was associated with improved OS (HR = 0.75, 95% CI: 0.60–0.95, *p* = 0.02; Fig. 4*B*). Among patients with STK11 mutation (*n* = 132), DICI-CT did not improve OS in the indirect comparison model (HR = 0.78, 95% CI: 0.46–1.31, *p* = 0.34; Fig. 4*C*). The PH assumption was violated (Supplementary Data 1, Supplementary Fig. 4*C*), and time-varying modeling suggested an early association favoring DICI-CT (Supplementary Data 1, Supplementary Fig. 3*E*). RMST analysis supported this early benefit, with a significant difference at 24 months (Supplementary Data 1, Supplementary Fig. 5*E*). In patients with STK11 wild-type tumors (*n* = 564), DICI-CT was associated with improved OS (HR = 0.68, 95% CI: 0.56–0.82, *p* < 0.001; Fig. 4*D*). Exploratory subgroup interaction analysis was nonsignificant (Supplementary Data 1, Supplementary Table 10).

**Figure 4:**
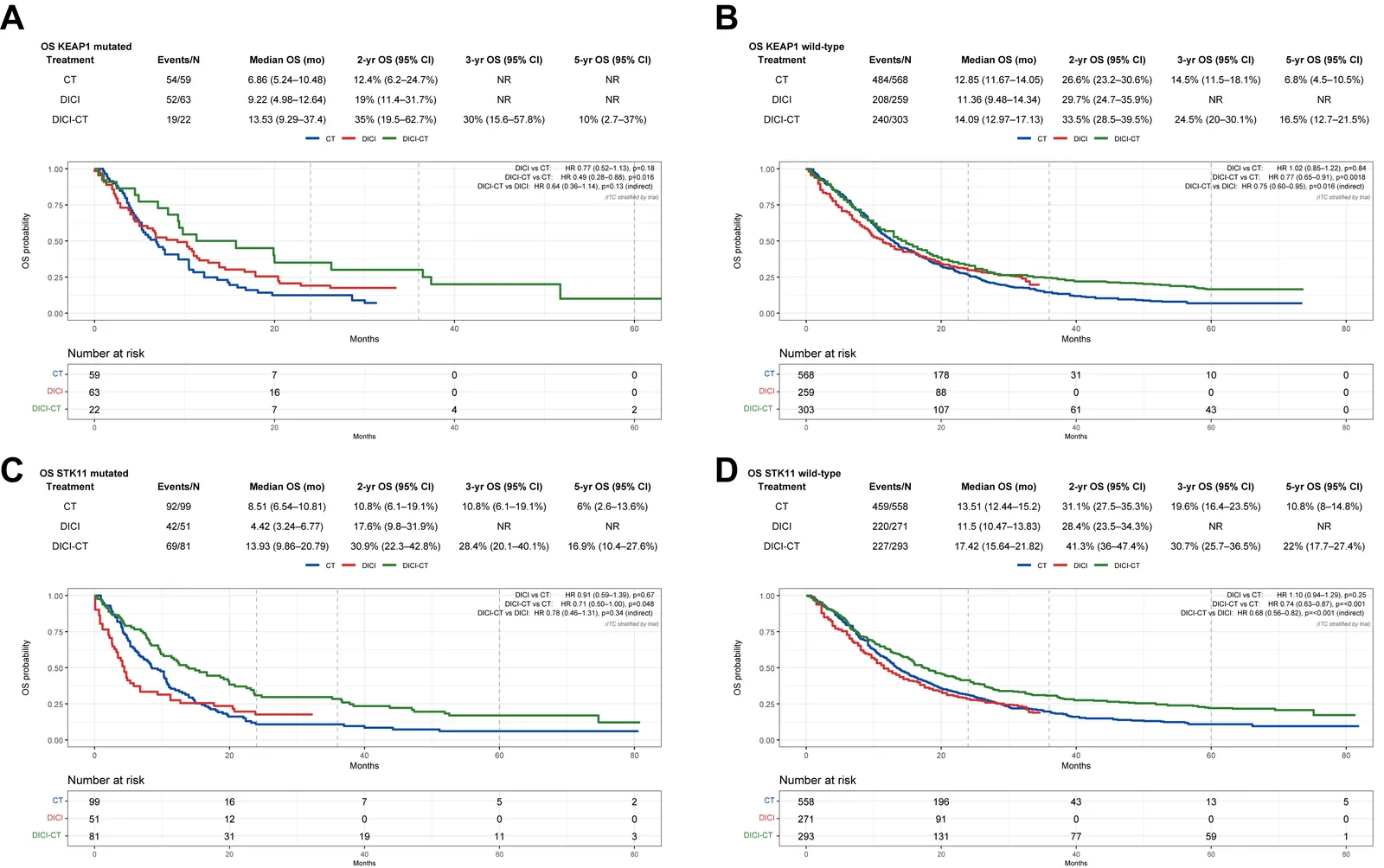
Overall survival with DICI and DICI-CT in patients with (*A*) KEAP1-mutated, (*B*) KEAP1 wild-type, (*C*) STK11-mutated, and (*D*) STK11 wild-type tumors. DICI, dual immune checkpoint inhibition; DICI-CT, dual immune checkpoint inhibition plus chemotherapy.

### Quality Assessment

All studies were judged to have a low risk of bias based on the assessed domains (Supplementary Data 1, Supplementary Figs. 6–7). Because fewer than 10 reports met the inclusion criteria, funnel plot asymmetry tests were not performed, consistent with the Cochrane methodological guidance.

### Discussion

This reconstructed individual patient-level meta-analysis evaluated the association between the addition of chemotherapy to DICI and clinical outcomes in treatment-naive metastatic NSCLC. Across four randomized trials (*N* = 1654), the addition of chemotherapy was not associated with a significant improvement in OS or PFS. Median OS was nearly identical between the strategies. Time-varying analyses suggested modest late survival differences favoring DICI; however, the absolute difference in 5-year OS was small (19.4% versus 17.1%). Collectively, these findings suggest that chemotherapy may not uniformly enhance the efficacy of DICI in unselected patients with metastatic NSCLC. As such, the addition of chemotherapy to DICI may be most clinically useful when rapid disease control is necessary.

These results should be interpreted in the context of pivotal trials, such as CheckMate 9LA and POSEIDON, which demonstrated improved OS with DICI-CT compared with CT.^5^^,^^10^ Importantly, neither trial directly compared DICI-CT with DICI. The current analysis addresses the evidence gap by leveraging reconstructed individual patient-level data across randomized trials evaluating dual checkpoint inhibition strategies, providing the first indirect estimate of the incremental contribution of chemotherapy beyond dual immunotherapy. Notably, the absence of a durable OS or PFS advantage with DICI-CT is consistent with emerging evidence of long-term survival plateaus associated with DICI, particularly in immunologically responsive tumors.^16^^,^^19^

However, design differences exist between these trials. In CheckMate 9LA, patients received nivolumab (360 mg every 3 weeks) plus ipilimumab (1 mg/kg every 6 weeks) with only 2 cycles of histology-directed platinum-doublet chemotherapy, a strategy intended to provide early disease control while minimizing prolonged cytotoxic exposure.^10^ In contrast, POSEIDON administered durvalumab (1500 mg) plus tremelimumab (75 mg) every 3 weeks with up to 4 cycles of platinum-doublet chemotherapy, followed by durvalumab 1500 mg every 4 weeks and one additional dose of tremelimumab 75 mg at week 16. The greater cumulative chemotherapy exposure in POSEIDON may have influenced durable immune-mediated responses. Importantly, neither trial directly compared DICI plus chemotherapy with DICI alone.

Prospective evidence supporting this observation is provided by the phase 2 Canadian CCTG BR34 trial, which directly compared durvalumab plus tremelimumab with or without platinum-based chemotherapy and did not demonstrate an OS improvement with the addition of chemotherapy (median OS 16.6 versus 14.1 mo; HR = 0.88, *p* = 0.46), despite an improvement in PFS (HR = 0.67).^20^ Although the trial was underpowered and limited by early closure, these findings are consistent with the current analysis and support the hypothesis that the addition of chemotherapy to dual immunotherapy may not confer a clinically meaningful survival advantage in unselected populations. Similarly, the randomized phase 3 JCOG2007 (NIPPON trial),^21^ comparing platinum-based chemotherapy plus nivolumab-ipilimumab with platinum-based chemotherapy plus pembrolizumab, was terminated early due to a high incidence of treatment-related deaths in the nivolumab-ipilimumab arm and did not reveal a significant OS difference between arms (median 23.7 versus 20.5 mo; HR = 0.98, *p* = 0.46), raising concerns about an unfavorable benefit-risk profile for the CTLA-4–containing regimen. Taken together, these findings suggest that the incremental contribution of chemotherapy to dual immunotherapy may be limited in unselected metastatic NSCLC. Importantly, these trials suggest that the benefits of chemotherapy, when present, tend to occur early, whereas long-term outcomes appear to be largely driven by the immunotherapy backbone. This distinction is clinically relevant, particularly in patients with aggressive disease biology or significant symptom burden.

The observed late differences favoring DICI are biologically plausible. Dual checkpoint blockade induces robust and sustained T-cell priming, clonal expansion, and immune memory,^22^ which could potentially be influenced by chemotherapy-related immunologic effects, including lymphodepletion and T-cell exhaustion.^23^ Although chemotherapy remains important for achieving rapid disease control in symptomatic patients with a high tumor burden, its addition may not be necessary or may not meaningfully improve long-term immune-mediated tumor control in selected patients. Furthermore, although chemotherapy may facilitate early immune priming through immunogenic cell death and antigen release, these effects may be insufficient to translate into meaningful long-term survival improvements at the population level. These observations underscore the importance of individualized treatment selection when considering DICI-based strategies. Clinical factors, including symptom burden and rapidly progressive disease, should inform the decision to add chemotherapy. In the absence of these considerations, this analysis suggests that proceeding with DICI alone may be reasonable in selected patients.

Methodologically, this analysis highlights the limitations of conventional PH models in immunotherapy trials. Delayed treatment effects and long-term survival plateaus are hallmark features of immune-based therapies and may be obscured by median survival estimates and constant HRs.^24^^,^^25^ Consistent with previous observations that PH assumptions are frequently violated in oncology, and particularly in immunotherapy studies,^24^^,^^25^ our use of time-varying hazard models and restricted mean survival time provides a more nuanced and clinically informative assessment of treatment effects.^13^

Exploratory subgroup analyses further suggest a time-dependent role for chemotherapy. In patients with nonsquamous histology and PD-L1 expression more than or equal to 1%, DICI was associated with numerically higher long-term survival, with late benefits emerging beyond 2 years of follow-up. In contrast, no meaningful differences were observed in PD-L1–negative or squamous populations. Among molecular subgroups, STK11-mutated tumors were the only group in which DICI-CT was associated with an early OS benefit. This finding is consistent with the known immune-cold phenotype and primary resistance to PD-(L)1 blockade in STK11-mutated tumors, which chemotherapy may partially overcome through transient immune priming.^26^ However, the absence of a sustained benefit in time-varying analyses suggests that chemotherapy may not fully overcome STK11-associated immune resistance^27^, ^28^, ^29^ and primarily contributes to short-term disease control rather than long-term survival.

Although nivolumab plus ipilimumab is not approved in the United States for PD-L1 less than 1%, its use in this population is common in clinical practice. From a clinical perspective, our findings suggest that both DICI and DICI-CT are effective first-line treatment options and that the decision to add chemotherapy should be made in consideration of the clinical context, including symptom burden and the need for rapid disease control. These findings generate the hypothesis that patients with STK11-mutated tumors may derive an early survival benefit from the addition of chemotherapy to DICI without a corresponding long-term survival advantage. Prospective trials incorporating molecular stratification are needed to validate this hypothesis and to optimize patient selection strategies.

Several limitations warrant consideration. First, reconstructed individual patient-level data derived from published KM curves cannot fully replicate true patient-level data sets, particularly with respect to covariate adjustment and post-progression therapies. Second, the number of KEAP1-mutated tumors treated with DICI-CT was limited, reducing the statistical power in this subgroup. Third, data were unavailable for DICI in KRAS-mutated or KRAS co-mutated populations. Fourth, heterogeneity existed across trials with respect to chemotherapy regimens, duration, and study design. Finally, clinically relevant imbalances in baseline characteristics across pooled treatment groups, including PD-L1 expression and histology, may have influenced subgroup and long-term survival comparisons.

In this cohort of patients with metastatic NSCLC, the addition of chemotherapy to DICI was not associated with a significant OS benefit. Exploratory subgroup analyses suggested numerically improved long-term outcomes with DICI in PD-L1–positive and nonsquamous subgroups, whereas DICI-CT conferred a limited early survival benefit in STK11-mutated tumors, which was not maintained with longer follow-up. Our findings support an individualized approach to using chemotherapy in combination with DICI.

## CRediT Authorship Contribution Statement

**Skyler Taylor**: Conceptualization, Writing - original draft, Writing - review & editing.

**Isadora Mamede**: Conceptualization, Data curation, Formal analysis, Investigation, Visualization, Writing - original draft.

**Gabriela Gazzoni**: Data curation, Investigation, Formal analysis, Visualization, Writing - review & editing.

**Israt Jahan Riya**: Validation, Visualization, Writing - review & editing.

**Ifrat Jahan Piya**: Validation, Visualization, Writing - review & editing.

**Carlos Stecca**: Writing - review & editing.

**Antonious Hazim**: Writing - review & editing.

**Guilherme Lopes**: Validation, Writing - review & editing.

**Vinicius Ernani**: Conceptualization, Supervision, Writing - review & editing.

## Data Availability Statement

The data that support the findings of this study will be shared on reasonable request to the corresponding author.

## Disclosure

Dr. Ernani reports having advisory roles with AstraZeneca, Johnson & Johnson, Daiichi Sankyo, Jazz Pharmaceuticals, Nuvation Bio, BioAtla, Novocure, Bayer, and Pfizer. Dr. Stecca reports receiving consulting fees, honoraria, and travel support and having advisory board participation from Johnson & Johnson, Adium, Bayer, Astellas, and AstraZeneca. The remaining authors declare no conflict of interest.
