# Supplementary material for: Dual Checkpoint Inhibition With or Without Chemotherapy in Metastatic NSCLC: A Reconstructed Individual Patient Data Meta-Analysis
Source: JTO Clin Res Rep. 2026 Aug 7;7(11):101059. doi: 10.1016/j.jtocrr.2026.101059 (PMC13694867; doi:10.1016/j.jtocrr.2026.101059)

SUPPLEMENTARY MATERIAL

Dual Checkpoint Inhibition With or Without Chemotherapy for Metastatic Non Small Cell Lung Cancer: A Reconstructed Individual Patient Data Meta-Analysis

Skyler Taylor MD*[a], Isadora Mamede MD*[b], Gabriela Gazzoni MD[c], Israt Jahan Riya[d], Ifrat Jahan Piya[d], Carlos Stecca MD[e], Guilherme Lopes[f], Antonious Hazim MD[a], Vinicius Ernani MD[a]
*Both authors contribute equally to this study

[a]Division of Hematology & Medical Oncology, Mayo Clinic Arizona, 85054, Phoenix, AZ, United States of America;
[b]Faculty of Medicine, Federal University of São João del Rei, 35501-296, Divinópolis, Brazil;
[c]Institute of Medical Assistance to the State Public Servant (IAMSPE), 04039-000 , São Paulo, Brazil;
[d]Faculty of Medicine, Dhaka Medical College and Hospital, 1000, Dhaka, Bangladesh;
[e]Department of Medical Oncology, BC Cancer – Abbotsford, V2S 0C2, Abbotsford, BC, Canada;
[f]Faculty of Medicine, Centro Universitário Faculdade de Medicina do ABC, 09060-870, Santo André, Brazil;

Corresponding Author:
Name: Vinicius Ernani
Email: Ernani.Vinicius@mayo.edu
Address: 5777 E Mayo Blvd, Phoenix, AZ, USA, 85054

TABLE OF CONTENTS

Supplementary Table 1. PRISMA 3

A) PRISMA 2020 for Abstracts Checklist. *4*

Supplementary Methods 1 - Search strategy 6

Supplementary Table 2. Baseline characteristics of included arms 7

Supplementary Table 3. Baseline characteristics of included studies 9

Supplementary Table 4. Median follow-up in each analysis 11

Supplementary Table 6. Median overall survival (OS) and 5-year OS rates reported by original studies and their corresponding values reconstructed through the IPDfromKM method in the CT arm. 13

Supplementary Table 7. Median overall survival (OS) and 5-year OS rates reported by original studies and their corresponding values reconstructed through the IPDfromKM method in the DICI +- CT arms 16

Supplementary Table 8. Aggregated data network meta-analysis OS results summary 19

Supplementary Table 9. Proportional hazard assumption based on the scaled Schoenfeld residuals in each analysis. 20

Supplementary Table 10. Subgroup interaction analysis in the DICI-CT vs DICI comparison 21

Supplementary Table 11. Summary of treatment effect measures for the DICI-CT versus DICI indirect comparison across all analyses 22

Supplementary Figure 1. Reconstructed Kaplan-Meier plots of individual studies 23

A) CheckMate 227 Part 1 .... *23*

B) CheckMate 9LA *25*

C) MYSTIC *27*

D) POSEIDON *29*

Supplementary Figure 2. Schoenfeld residuals plots: OS and PFS (all patients; PD-L1 ≥ 1%; PD-L1 < 1%; Squamous and Nonqsquamous patients) 32

Supplementary Figure 3. Time-varying hazard ratios 33

Supplementary Figure 4. Schoenfeld residuals plots: OS (KEAP1 mutated and wild-type; KTK11 mutated and wild-type patients) 34

Supplementary Figure 5. Restricted Mean Survival Time plots 35

Supplementary Figure 6 - Traffic light plot of RoB assessment 36

Supplementary Figure 7 - Summary plot of RoB assessment 36

Supplementary Table 1. PRISMA

Section and Topic	Item #	Checklist item	Location where item is reported
TITLE			
Title	1	Identify the report as a systematic review.	Title Page
ABSTRACT			
Abstract	2	See the PRISMA 2020 for Abstracts checklist.	eTable 1
INTRODUCTION			
Rationale	3	Describe the rationale for the review in the context of existing knowledge.	Page 4
Objectives	4	Provide an explicit statement of the objective(s) or question(s) the review addresses.	Page 4
METHODS			
Eligibility criteria	5	Specify the inclusion and exclusion criteria for the review and how studies were grouped for the syntheses.	Page 5
Information sources	6	Specify all databases, registers, websites, organisations, reference lists and other sources searched or consulted to identify studies. Specify the date when each source was last searched or consulted.	Page 5
Search strategy	7	Present the full search strategies for all databases, registers and websites, including any filters and limits used.	Page 5
Selection process	8	Specify the methods used to decide whether a study met the inclusion criteria of the review, including how many reviewers screened each record and each report retrieved, whether they worked independently, and if applicable, details of automation tools used in the process.	Page 5
Data collection process	9	Specify the methods used to collect data from reports, including how many reviewers collected data from each report, whether they worked independently, any processes for obtaining or confirming data from study investigators, and if applicable, details of automation tools used in the process.	Page 5-7
Data items	10a	List and define all outcomes for which data were sought. Specify whether all results that were compatible with each outcome domain in each study were sought (e.g. for all measures, time points, analyses), and if not, the methods used to decide which results to collect.	Page 5-7
	10b	List and define all other variables for which data were sought (e.g. participant and intervention characteristics, funding sources). Describe any assumptions made about any missing or unclear information.	Page 5-7
Study risk of bias assessment	11	Specify the methods used to assess risk of bias in the included studies, including details of the tool(s) used, how many reviewers assessed each study and whether they worked independently, and if applicable, details of automation tools used in the process.	Page 7
Effect measures	12	Specify for each outcome the effect measure(s) (e.g. risk ratio, mean difference) used in the synthesis or presentation of results.	Page 5-7
Synthesis methods	13a	Describe the processes used to decide which studies were eligible for each synthesis (e.g. tabulating the study intervention characteristics and comparing against the planned groups for each synthesis (item #5)).	Page 5-7
	13b	Describe any methods required to prepare the data for presentation or synthesis, such as handling of missing summary statistics, or data conversions.	Page 5-7
	13c	Describe any methods used to tabulate or visually display results of individual studies and syntheses.	Page 5-7
	13d	Describe any methods used to synthesize results and provide a rationale for the choice(s). If meta-analysis was performed, describe the model(s), method(s) to identify the presence and extent of statistical heterogeneity, and software package(s) used.	Page 5-7
	13e	Describe any methods used to explore possible causes of heterogeneity among study results (e.g. subgroup analysis, meta-regression).	Page 7
	13f	Describe any sensitivity analyses conducted to assess robustness of the synthesized results.	Page 7
Reporting bias assessment	14	Describe any methods used to assess risk of bias due to missing results in a synthesis (arising from reporting biases).	Page 7
Certainty assessment	15	Describe any methods used to assess certainty (or confidence) in the body of evidence for an outcome.	NA

RESULTS			
Study selection	16a	Describe the results of the search and selection process, from the number of records identified in the search to the number of studies included in the review, ideally using a flow diagram.	Page 7
	16b	Cite studies that might appear to meet the inclusion criteria, but which were excluded, and explain why they were excluded.	Page 7
Study characteristics	17	Cite each included study and present its characteristics.	Page 7
Risk of bias in studies	18	Present assessments of risk of bias for each included study.	Page 9
Results of individual studies	19	For all outcomes, present, for each study: (a) summary statistics for each group (where appropriate) and (b) an effect estimate and its precision (e.g. confidence/credible interval), ideally using structured tables or plots.	Page 8-9
Results of syntheses	20a	For each synthesis, briefly summarise the characteristics and risk of bias among contributing studies.	Page 9
	20b	Present results of all statistical syntheses conducted. If meta-analysis was done, present for each the summary estimate and its precision (e.g. confidence/credible interval) and measures of statistical heterogeneity. If comparing groups, describe the direction of the effect.	Page 8-9
	20c	Present results of all investigations of possible causes of heterogeneity among study results.	Page 7-9
	20d	Present results of all sensitivity analyses conducted to assess the robustness of the synthesized results.	Page 7-9
Reporting biases	21	Present assessments of risk of bias due to missing results (arising from reporting biases) for each synthesis assessed.	Page 9
Certainty of evidence	22	Present assessments of certainty (or confidence) in the body of evidence for each outcome assessed.	NA
DISCUSSION			
Discussion	23a	Provide a general interpretation of the results in the context of other evidence.	Page 8-12
	23b	Discuss any limitations of the evidence included in the review.	Page 12
	23c	Discuss any limitations of the review processes used.	Page 12
	23d	Discuss implications of the results for practice, policy, and future research.	Page 12
OTHER INFORMATION			
Registration and protocol	24a	Provide registration information for the review, including register name and registration number, or state that the review was not registered.	Page 5
	24b	Indicate where the review protocol can be accessed, or state that a protocol was not prepared.	Page 5
	24c	Describe and explain any amendments to information provided at registration or in the protocol.	Page 5
Support	25	Describe sources of financial or non-financial support for the review, and the role of the funders or sponsors in the review.	Page 13
Competing interests	26	Declare any competing interests of review authors.	Page 13
Availability of data, code and other materials	27	Report which of the following are publicly available and where they can be found: template data collection forms; data extracted from included studies; data used for all analyses; analytic code; any other materials used in the review.	Page 13

From: Page MJ, McKenzie JE, Bossuyt PM, Boutron I, Hoffmann TC, Mulrow CD, et al. The PRISMA 2020 statement: an updated guideline for reporting systematic reviews. BMJ 2021;372:n71. doi: 10.1136/bmj.n71[1]
For more information, visit: http://www.prisma-statement.org

A) PRISMA 2020 for Abstracts Checklist.

Section and Topic	Item #	Checklist item	Reported (Yes/No)
TITLE			

Title	1	Identify the report as a systematic review.	yes
BACKGROUND			
Objectives	2	Provide an explicit statement of the main objective(s) or question(s) the review addresses.	yes
METHODS			
Eligibility criteria	3	Specify the inclusion and exclusion criteria for the review.	yes
Information sources	4	Specify the information sources (e.g. databases, registers) used to identify studies and the date when each was last searched.	yes
Risk of bias	5	Specify the methods used to assess risk of bias in the included studies.	no
Synthesis of results	6	Specify the methods used to present and synthesise results.	yes
RESULTS			
Included studies	7	Give the total number of included studies and participants and summarise relevant characteristics of studies.	yes
Synthesis of results	8	Present results for main outcomes, preferably indicating the number of included studies and participants for each. If meta-analysis was done, report the summary estimate and confidence/credible interval. If comparing groups, indicate the direction of the effect (i.e. which group is favoured).	yes
DISCUSSION			
Limitations of evidence	9	Provide a brief summary of the limitations of the evidence included in the review (e.g. study risk of bias, inconsistency and imprecision).	no
Interpretatio n	10	Provide a general interpretation of the results and important implications.	yes
OTHER INFORMATION			
Funding	11	Specify the primary source of funding for the review.	no
Registration	12	Provide the register name and registration number	no

From: Page MJ, McKenzie JE, Bossuyt PM, Boutron I, Hoffmann TC, Mulrow CD, et al. The PRISMA 2020 statement: an updated guideline for reporting systematic reviews. BMJ 2021;372:n71. doi: 10.1136/bmj.n71[1]
For more information, visit: http://www.prisma-statement.org

Supplementary Methods 1 - Search strategy

PUBMED: 220 results

("non-small cell lung cancer" OR NSCLC OR "lung cancer" OR "lung carcinoma") AND (metastatic OR advanced OR "stage IV" OR recurrent) AND (mutation* OR mutated OR mutant OR alteration* OR gene OR KRAS OR STK11 OR KEAP1) AND ("first-line" OR "treatment-naïve" OR untreated OR "initial therapy") AND ("programmed death-ligand 1 inhibitor" OR "programmed cell death protein-1 inhibitor" OR "PD-1 inhibitor" OR "PD-L1 inhibitor" OR "anti-PD-1" OR "anti-PD-L1" OR "anti-PD1" OR "anti PDL1" OR "immune checkpoint inhibitor" OR ICI OR nivolumab OR pembrolizumab OR atezolizumab OR durvalumab OR cemiplimab OR "CTLA-4 inhibitor" OR "anti-CTLA-4" OR ipilimumab OR tremelimumab OR immunotherapy) AND (randomized controlled trial[pt] OR controlled clinical trial[pt] OR clinical trials as topic[mesh:noexp] OR trial[ti] OR random*[tiab] OR placebo*[tiab] OR "phase 3")

EMBASE: 2246 results

('non-small cell lung cancer' OR NSCLC OR 'lung cancer' OR 'lung carcinoma') AND (metastatic OR advanced OR 'stage IV' OR recurrent) AND (mutation* OR mutated OR mutant OR alteration* OR gene OR KRAS OR STK11 OR KEAP1) AND ('first-line' OR 'treatment-naïve' OR untreated OR 'initial therapy') AND ('programmed death-ligand 1 inhibitor' OR 'programmed cell death protein-1 inhibitor' OR 'PD-1 inhibitor' OR 'PD-L1 inhibitor' OR 'anti-PD-1' OR 'anti-PD-L1' OR 'anti-PD1' OR 'anti-PDL1' OR 'immune checkpoint inhibitor' OR ICI OR nivolumab OR pembrolizumab OR atezolizumab OR durvalumab OR cemiplimab OR 'CTLA-4 inhibitor' OR 'anti-CTLA-4' OR ipilimumab OR tremelimumab OR immunotherapy) AND ('randomized controlled trial'/exp OR 'controlled clinical trial'/exp OR 'clinical trial'/exp OR trial OR random* OR placebo OR 'phase 3')

Cochrane: 696 results

("non-small cell lung cancer" OR NSCLC OR "lung cancer" OR "lung carcinoma") AND (metastatic OR advanced OR "stage IV" OR recurrent) AND (mutation OR mutated OR mutant OR alteration OR gene OR KRAS OR STK11 OR KEAP1) AND ("first-line" OR "treatment-naïve" OR untreated OR "initial therapy") AND ("programmed death-ligand 1 inhibitor" OR "programmed cell death protein-1 inhibitor" OR "PD-1 inhibitor" OR "PD-L1 inhibitor" OR "anti-PD-1" OR "anti-PD-L1" OR "anti- PD1" OR "anti-PDL1" OR "immune checkpoint inhibitor" OR ICI OR nivolumab OR pembrolizumab OR atezolizumab OR durvalumab OR cemiplimab OR "CTLA-4 inhibitor" OR "anti-CTLA-4" OR ipilimumab OR tremelimumab OR immunotherapy) AND ("randomized controlled trial" OR "controlled clinical trial" OR "clinical trial" OR trial OR placebo OR "phase 3")

ClinicalTrials.gov: 115 results

("lung cancer" OR "non-small cell lung cancer" OR NSCLC) | Other terms: (metastatic OR advanced OR "stage IV" OR recurrent) AND (mutation OR mutated OR mutant OR alteration OR gene OR KRAS OR STK11 OR KEAP1) AND ("first-line" OR "treatment-naïve" OR untreated OR "initial therapy") | ('programmed death-ligand 1 inhibitor' OR 'programmed cell death protein-1 inhibitor' OR 'PD-1 inhibitor' OR 'PD-L1 inhibitor' OR 'anti-PD-1' OR 'anti-PD-L1' OR 'anti-PD1' OR 'anti-PDL1' OR 'immune checkpoint inhibitor' OR ICI OR nivolumab OR pembrolizumab OR atezolizumab OR durvalumab OR cemiplimab OR 'CTLA-4 inhibitor' OR 'anti-CTLA-4' OR ipilimumab OR tremelimumab OR immunotherapy) | Interventional studies

Supplementary Table 2. Baseline characteristics of included arms

Characteristic	DICI-CT	DICI	SMD (95% CI)	p-value
N	699	955		
Age	63.7 (8.8)	63.7 (9.7)	0.000 (-0.098; 0.098)	1
**Sex**			-0.123 (-0.221; -0.025)	0.014
Female	178 (25.5%)	296 (31.0%)		
Male	521 (74.5%)	659 (69.0%)		
**Smoking status**			-0.004 (-0.102; 0.093)	0.657
Current/former	594 (85.0%)	813 (85.1%)		
Never	105 (15.0%)	135 (14.1%)		
Missing	0 (0%)	7 (0.7%)		
**ECOG PS**			-0.119 (-0.216; -0.021)	0.028
0	224 (32.0%)	360 (37.7%)		
1	474 (67.8%)	593 (62.1%)		
2	0 (0%)	2 (0.2%)		
Missing	1 (0.1%)	0 (0%)		
**Histology**			-0.126 (-0.223; -0.028)	0.01
Nonsquamous	460 (65.8%)	684 (71.6%)		
Squamous	239 (34.2%)	270 (28.3%)		
Missing	0 (0%)	1 (0.1%)		
**PD-L1 TPS**			0.208 (0.110; 0.305)	<0.001
<1%	260 (37.2%)	263 (27.5%)		
≥1%	417 (59.7%)	692 (72.5%)		
Missing	22 (3.1%)	0 (0%)		
**Baseline brain metastasis**				
No	601 (86.0%)	NA		
Yes	98 (14.0%)	NA		
**Mutation-evaluable**				
Yes	491 (70.2%)	NA		
**KEAP1**			-0.129 (-0.226; -0.032)	<0.001
Mutant	38 (5.4%)	83 (8.7%)		
Wild-type	453 (64.8%)	477 (49.9%)		

**STK11**			0.139 (0.041; 0.237)	0.119
Mutant	97 (13.9%)	90 (9.4%)		
Wild-type	394 (56.4%)	470 (49.2%)		
**KRAS**				
Mutant	133 (19.0%)	NA		
Wild-type	358 (51.2%)	NA		

CI = Confidence Interval; CT = chemotherapy; DICI = Dual Immune Checkpoint Inhibition; DICI-CT = Dual Immune Checkpoint Inhibition plus Chemotherapy; ECOG PS = Eastern Cooperative Oncology Group Performance Status; ICI = immune checkpoint inhibitor; NA = not available; PD-L1 = programmed death-ligand 1; SMD = Standardized Mean Difference; TPS = tumor proportion score.

Supplementary Table 3. Baseline characteristics of included studies

Trial	CM 227 Part 1		CM 9LA	MYSTIC	POSEIDON
**Characteristic**	**NI, PD-L1 <1% (N=187)**	**NI, PD-L1 ≥1% (N=396)**	**NIC (N=361)**	**DT (N=372)**	**DTC (N=338)**
Age	63 (34–87)	64 (26–84)	65 (35–81)	64.3 (9.52)	63 (27-83)
**Sex**					
Female	49 (26.2%)	141 (35.6%)	109 (30.2%)	106 (28.5%)	69 (20.4%)
Male	138 (73.8%)	255 (64.4%)	252 (69.8%)	266 (71.5%)	269 (79.6%)
**Smoking status**					
Current/former	163 (87.2%)	334 (84.3%)	315 (87.3%)	316 (84.9%)	279 (82.5%)
Never	23 (12.3%)	56 (14.1%)	46 (12.7%)	56 (15.1%)	59 (17.5%)
Missing	1 (0.5%)	6 (1.5%)	0 (0%)	0 (0%)	0 (0%)
**ECOG PS**					
0	69 (36.9%)	135 (34.1%)	114 (31.6%)	156 (41.9%)	110 (32.5%)
1	117 (62.6%)	260 (65.7%)	246 (68.1%)	216 (58.1%)	228 (67.5%)
2	1 (0.5%)	1 (0.3%)	0 (0%)	0 (0%)	0 (0%)
Missing	0 (0%)	0 (0%)	1 (0.3%)	0 (0%)	0 (0%)
**Histology**					
Non-squamous	140 (74.9%)	279 (70.5%)	246 (68.1%)	265 (71.2%)	214 (63.3%)
Squamous	46 (24.6%)	117 (29.5%)	115 (31.9%)	107 (28.8%)	124 (36.7%)
Missing	1 (0.5%)	0 (0%)	0 (0%)	0 (0%)	0 (0%)
**PD-L1 TPS**					
<1%	187 (100%)	0 (0%)	135 (37.4%)	76 (20.4%)	125 (37.0%)
≥1%	0 (0%)	396 (100%)	204 (60.2%)	296 (79.6)	213 (63.0%)
Missing	0 (0%)	0 (0%)	22 (6.1%)	0 (0%)	0 (0%)
**Baseline brain metastasis**					
No	164 (87.7%)	355 (89.6%)	296 (82%)	NA	305 (90.2%)
Yes	23 (12.3%)	41 (10.4%)	65 (18%)	NA	33 (9.8%)
**Mutation-evaluable**					
Yes	475 (81.5%)		166 (46%)	NA	325 (96.2%)
**KEAP1**					
Mutant	20 (3.4%)		16 (4.4%)	63 (16.9%)	22 (6.8%)
Wild-type	218 (37.4%)		150 (41.6%)	259 (69.6%)	303 (93.2%)
**STK11**					
Mutant	39 (6.7%)		50 (13.8%)	51 (13.7%)	47 (14.5%)
Wild-type	199 (34.1%)		116 (32.1%)	271 (72.8%)	278 (85.5%)
**KRAS**					

Trial	CM 227 Part 1	CM 9LA	MYSTIC	POSEIDON
Mutant	88 (15.1%)	61 (16.9%)	NA	72 (22.2%)
Wild-type	150 (25.7%)	105 (29.1%)	NA	253 (77.8%)

CM = CheckMate; DT= Durvalumab + Tremelimumab; DTC = Durvalumab + Tremelimumab + Chemotherapy; NA = not available; NI = Nivolumab + Ipilimumab; NIC = Nivolumab + Ipilimumab + Chemotherapy; PD-L1 = programmed death-ligand 1;

Supplementary Table 4. Median follow-up in each analysis.

Outcome	Median Follow-up months
**OS**	
All patients	65.5 (95% CI 65.5–66.7)
KEAP1mut	33.6 (95% CI 33.6–NA)
KEAP1wt	61.7 (95% CI 34.6–63.4)
STK11mut	67.8 (95% CI 55.8–80.6)
STK11wt	62.9 (95% CI 61.4–64.9)
PD-L1 ≥ 1%	65.5 (95% CI 60.5–65.5)
PD-L1 < 1%	65 (95% CI 65–66.8)
SQ	61 (95% CI 56.2–69.7)
NonSQ	59.3 (95% CI 58.3–62.1)
**PFS**	
All patients	37.1 (95% CI 23.2–60.3)

mut = mutated; NA = not available; NonSQ = non-squamous; OS = overall survival; PD-L1 = programmed death-ligand 1; PFS = progression-free survival; SQ = squamous; wt = wild-type.

Supplementary Table 5. Studies included in each analysis

Trial	CM 227 Part 1	CM 9LA	MYSTIC	POSEIDON
OS All patients	X (PD-L1 ≥1% and PD-L1 < 1% data)	X	X	X
OS KEAP1mut			X	X
OS KEAP1wt			X	X
OS STK11mut		X	X	X
OS STK11wt		X	X	X
OS PD-L1 ≥1%	X	X	X (PD-L1 ≥25%)	X
OS PD-L1 < 1%	X	X		X
OS SQ	X (PD-L1 ≥1% and PD-L1 < 1% data)	X		X
OS NonSQ	X (PD-L1 ≥1% and PD-L1 < 1% data)	X		X
PFS All patients	X	X	X (PD-L1 ≥25%)	X

CM = CheckMate; mut = mutated; NA = not available; NonSQ = non-squamous; OS = overall survival; PD-L1 = programmed death-ligand 1; PFS = progression-free survival; SQ = squamous; wt = wild-type.

Supplementary Table 6. Median overall survival (OS) and 5-year OS rates reported by original studies and their corresponding values reconstructed through the IPDfromKM method in the CT arm.

TRIAL	CT regimen	ANALYSIS	SOURCE	MEDIAN OS	5y SURVIVAL
OS all patients					
POSEIDON	Platinum-doublet (every 3 wk for 6 cycles)	OS all	Original	11.6 (10.5-13.1)	6.8% (4.4-10.0%)
			Reconstructed	11.8 (10.7–13.3)	7.3% (4.8–10.9%)
CM9LA	Platinum-doublet (every 3 wk for 4 cycles)	OS all	Original	11.0 (9.5-12.7)	11% (NA-NA)
			Reconstructed	11.3 (10–13.7)	10.8% (7.9–14.7%)
MYSTIC	Platinum-doublet (4 to 6 cycles)	OS all	Original	11.8 (10.5-13.3)	24.1% (19.8-28.6%)^
			Reconstructed	12 (10.6–13.6)	24.7% (20.6–29.7%)^
PD-L1 Status					
POSEIDON	Platinum-doublet (every 3 wk for 6 cycles)	PD-L1 ≥ 1 %	Original	12.2 (10.4-15.2)	8.6% (5.2-13.1%)
			Reconstructed	12.4 (10.4–15.1)	9.1% (5.8–14.4%)
POSEIDON		PD-L1 < 1%	Original	11.0 (8.7-12.7)	4.0% (1.5-8.6%)
			Reconstructed	10.8 (8.9–13.3)	4.2% (1.8–9.9%)
MYSTIC	Platinum-doublet (4 to 6 cycles)	PD-L1 ≥ 1 %	Original	12.9 (10.5-15.0)	22.7% (16.5-29.5%)
			Reconstructed	13.1 (10.8–15.2)	22.7% (17–30.3%)^
CM9LA	Platinum-doublet (every 3 wk for 4 cycles)	PD-L1 ≥ 1 %	Original	10.9 (9.5-13.2)	11% (NA-NA)
			Reconstructed	10.6 (9.7–13.2)	10.8% (7.1–16.3%)
CM9LA		PD-L1 < 1%	Original	9.8 (7.7-13.5)	8% (NA-NA)
			Reconstructed	10 (7.9–13.7)	8.1% (4.4–14.9%)
CM 227 PART 1	Platinum-doublet (every 3 wk for 4 cycles)	PD-L1 ≥ 1 %	Original	14.9 (12.7-16.7)	14% (NA-NA)
			Reconstructed	15 (13–16.7)	14.4% (11.2–18.5%)
CM 227 PART 1		PD-L1 < 1%	Original	12.2 (9.2-14.3)	7% (NA-NA)
			Reconstructed	12.2 (9.2–14.7)	7% (4.1–12%)
KEAP1 Mutation Status					
POSEIDON	Platinum-doublet (every 3 wk for 6 cycles)	KEAP1 mut	Original	8.7 (5.1-NA)	NA
			Reconstructed	8.7 (6–NA)	NA
POSEIDON		KEAP1 wt	Original	12.1 (10.6-13.9)	7% (4.4-10.3%)
			Reconstructed	12 (10.7–14.1)	6.4% (4–10.2%)
MYSTIC	Platinum-doublet (4 to 6 cycles)	KEAP1 mut	Original	6.3 (4.4-9.5)	NA
			Reconstructed	6.2 (4.8–10.5)	13.2% (6.6–26.3%)^
MYSTIC		KEAP1 wt	Original	13.3 (11.6-15.5)	NA
			Reconstructed	13.6 (12.1–15.9)	29.3% (24.2–35.4%)^
STK11 Mutation Status					
POSEIDON	Platinum-doublet (every 3 wk for 6 cycles)	STK11 mut	Original	10.7 (6.0-14.9)	4.5 (0.3-18.9)^
			Reconstructed	10.5 (6.8–14.9)	4.5% (0.7–30.8%)^
POSEIDON		STK11 wt	Original	13.4 (11.5-16.8)	10.4% (6.3-15.6%)

TRIAL	CT regimen	ANALYSIS	SOURCE	MEDIAN OS	5y SURVIVAL
			Reconstructed	13.3 (12–17.9)	10.5% (6.7–16.4%)
MYSTIC	Platinum-doublet (4 to 6 cycles)	STK11 mut	Original	6.7 (4.5-10.1)	NA
			Reconstructed	6.5 (4.9–10.3)	2.4% (0.4–16.9%)^
MYSTIC		STK11 wt	Original	13.1 (11.6-15.1)	NA
			Reconstructed	13.4 (11.9–15.6)	29.9% (24.8–35.9%)^
CM9LA	Platinum-doublet (every 3 wk for 4 cycles)	STK11 mut	Original	10.7 (5.4-14.9)	16% (NA-NA)
			Reconstructed	10.6 (7.4–19)	15.3% (6.9–33.7%)
CM9LA		STK11 wt	Original	13.9 (10.6-17.4)	13% (NA-NA)
			Reconstructed	14.1 (11.5–18.1)	14.3% (8.8–23.2%)
Histology					
POSEIDON	Platinum-doublet (every 3 wk for 6 cycles)	SQ	Original	10.5 (8.0-11.7)	2.9% (0.8-7.3%)
			Reconstructed	10.7 (8.9–12)	2.7% (0.8–9.2%)
POSEIDON		NonSQ	Original	13.0 (10.6-15.1)	9.1% (5.6-13.6%)
			Reconstructed	13.1 (11.3–15.6)	9.3% (6–14.5%)
CM9LA	Platinum-doublet (every 3 wk for 4 cycles)	SQ	Original	9.1 (7.2-11.6)	7% (NA-NA)
			Reconstructed	9.2 (7.4–12.4)	6.4% (3.1–13.1%)
CM9LA		NonSQ	Original	12.0 (9.9-13.9)	12% (NA-NA)
			Reconstructed	12 (10–14.1)	12.6% (8.9–17.8%)
CM 227 PART 1	Platinum-doublet (every 3 wk for 4 cycles)	SQ PD-L1 ≥ 1%	Original	9.2 (7.6-13.9)	19% (NA-NA)^
			Reconstructed	9.2 (7.8–14.1)	20% (13.9–28.8%)^
CM 227 PART 1		SQ PD-L1 < 1%	Original	8.5 (6.4-13.0)	16% (NA-NA)^
			Reconstructed	8.6 (6.9–15.2)	14.6% (7–30.1%)^
CM 227 PART 1		NonSQ PD-L1 ≥ 1%	Original	17.2 (14.3-19.6)	39% (NA-NA)^
			Reconstructed	17.2 (14.6–19.9)	39.3% (33.9–45.6%)^
CM 227 PART 1		NonSQ PD-L1 < 1%	Original	13.1 (9.8-15.3)	25% (NA-NA)^
			Reconstructed	13.1 (10.1–15.3)	25.2% (18.9–33.6%)^
PFS					
POSEIDON	Platinum-doublet (every 3 wk for 6 cycles)	PFS all	Original	4.8 (4.6-5.8)	NA
			Reconstructed	4.8 (4.6–5.8)	NA
CM9LA	Platinum-doublet (every 3 wk for 4 cycles)	PFS all	Original	5.3 (4.4-5.6)	5% (NA-NA)
			Reconstructed	5.2 (4.6–5.7)	4.3% (2.4–7.8%)
MYSTIC	Platinum-doublet (4 to 6 cycles)	PD-L1 ≥ 25%	Original	5.4 (4.6-5.8)	NA
			Reconstructed	5.4 (4.8–6.2)	NA
CM 227 PART 1	Platinum-doublet (every 3 wk for 4 cycles)	PD-L1 ≥ 1 %	Original	5.6 (4.6-5.8)	2% (NA-NA)
			Reconstructed	5.5 (4.6–5.7)	2.3% (0.9–5.9%)
CM 227 PART 1		PD-L1 < 1 %	Original	4.7 (4.2-5.6)	2% (NA-NA)

TRIAL	CT regimen	ANALYSIS	SOURCE	MEDIAN OS	5y SURVIVAL
			Reconstructed	5 (4.1–5.5)	1.7% (0.4–6.5%)

^24 months survival

CM = CheckMate; CT = Chemotherapy; mut = mutated; NA = not available; NonSQ = non-squamous; OS = overall survival; PD-L1 = programmed death-ligand 1; PFS = progression-free survival; SQ = squamous; wt = wild-type; wk = weeks

Supplementary Table 7. Median overall survival (OS) and 5-year OS rates reported by original studies and their corresponding values reconstructed through the IPDfromKM method in the DICI +- CT arms.

TRIAL	ANALYSIS	SOURCE	MEDIAN OS	5y SURVIVAL
OS all patients				
POSEIDON	OS all	Original	14.0 (11.7-16.1)	15.7% (12.0-19.9%)
		Reconstructed	14.1 (12–16.5)	15.5% (12–20.1%)
CM9LA	OS all	Original	15.8 (13.9-19.7)	18% (NA-NA)
		Reconstructed	15.7 (13.8–19.7)	18.4% (14.8–22.9%)
MYSTIC	OS all	Original	11.2 (9.5-12.9)	29.5% (24.9-34.2%)^
		Reconstructed	11.2 (9.6–12.9)	29% (24.7–34%)^
PD-L1 Status				
POSEIDON	PD-L1 ≥ 1 %	Original	15.6 (11.6-18.1)	20.8% (15.6-26.6%)
		Reconstructed	15.8 (11.5–19.6)	21.6% (16.2–28.8%)
POSEIDON	PD-L1 < 1%	Original	12.7 (9.9-15.5)	6.1% (2.6-11.8%)
		Reconstructed	12.9 (10.7–16.6)	6.4% (3–13.6%)
MYSTIC	PD-L1 ≥ 1 %	Original	11.9 (9.0-17.7)	35.4% (28.1-42.8%)^
		Reconstructed	12 (9.2–18.3)	35% (28.3–43.3%)^
CM9LA	PD-L1 ≥ 1 %	Original	15.8 (13.8-22.2)	18% (NA-NA)
		Reconstructed	15.7 (13.8–22.9)	17.7% (13.1–23.9%)
CM9LA	PD-L1 < 1%	Original	17.7 (13.7-20.3)	22% (NA-NA)
		Reconstructed	17.9 (14.1–22.0)	21.7% (15.7–30%)
CM 227 PART 1	PD-L1 ≥ 1 %	Original	17.1 (15.0-20.2)	24% (NA-NA)
		Reconstructed	17.1 (15.2–20.3)	23.9% (20–28.6%)
CM 227 PART 1	PD-L1 < 1%	Original	17.4 (13.2-22.0)	19% (NA-NA)
		Reconstructed	17.5 (13.4–23.7)	18.9% (13.9–25.6%)
KEAP1 Mutation Status				
POSEIDON	KEAP1 mut	Original	13.7(7.2-26.5)	10.0%(1.7-27.1%)
		Reconstructed	13.5 (9.3–37.4)	10% (2.7–37%)
POSEIDON	KEAP1 wt	Original	14.0 (11.8-16.1)	16.2%(12.2-20,6)
		Reconstructed	14.1 (13–17.1)	16.5% (12.7–21.5%)
MYSTIC	KEAP1 mut	Original	9.2 (4.6-11.2)	NA
		Reconstructed	9.2 (5–12.6)	19% (11.4–31.7%)^
MYSTIC	KEAP1 wt	Original	11.3 (9.3-13.7)	NA
		Reconstructed	11.4 (9.5–14.3)	29.7% (24.7–35.9%)^
STK11 Mutation Status				
POSEIDON	STK11 mut	Original	15.0 (8.2-23.8)	12.9% (4.1-27%)
		Reconstructed	14.9 (9.4–35.2)	12.9% (5.2–32.2%)

TRIAL	ANALYSIS	SOURCE	MEDIAN OS	5y SURVIVAL
POSEIDON	STK11 wt	Original	17.4 (154.9-22.1)	22.0% (16.1-28.5%)
		Reconstructed	17.4 (15.3–24.8)	22.1% (16.6–29.4%)
MYSTIC	STK11 mut	Original	4.4 (3.3-9.2)	NA
		Reconstructed	4.42(3.2–6.8)	17.6% (9.8–31.9%)^
MYSTIC	STK11 wt	Original	11.3 (9.7-13.7)	NA
		Reconstructed	11.5 (10.5–13.8)	28.4% (23.5–34.3%)^
CM9LA	STK11 mut	Original	13.8 (8.6-27.7)	19% (NA-NA)
		Reconstructed	13.8 (9.5–23.6)	19.4% (10.9–34.4%)
CM9LA	STK11 wt	Original	17.8 (13.2-22.8)	22% (NA-NA)
		Reconstructed	17.9 (14–22.9)	21.9% (15.5–30.9%)
Histology				
POSEIDON	SQ	Original	10.4 (8.4-12.7)	7.3% (3.4-13.1%)
		Reconstructed	10.6 (9.1–13.2)	7.1% (3.6–13.8%)
POSEIDON	NonSQ	Original	17.2 (14.9-21.8)	20.5% (15.3-26.2%)
		Reconstructed	17.2 (15.34–22)	20.9% (16–27.3%)
CM9LA	SQ	Original	14.5 (13.-19.3)	18% (NA-NA)
		Reconstructed	14.6 (13.6–19.8)	18.3% (12.4–27%)
CM9LA	NonSQ	Original	17.8 (14.1-20.7)	19% (NA-NA)
		Reconstructed	17.63 (14.4–22.3)	19.1% (14.7–24.7%)
CM 227 PART 1	SQ PD-L1 ≥ 1%	Original	14.8 (12.1-18.7)	NA
		Reconstructed	14.9 (12.4–19.3)	19.6% (13.4–28.5%)
CM 227 PART 1	SQ PD-L1 < 1%	Original	15.9 (9.0-33.9)	37% (NA-NA)^
		Reconstructed	15.6 (11.7–37.2)	37.2% (25.3–54.6%)^
CM 227 PART 1	NonSQ PD-L1 ≥ 1%	Original	19.4 (15.6-24.3)	44% (NA-NA)^
		Reconstructed	19.4 (15.7–24.6)	43.7% (38.2–50.1%)^
CM 227 PART 1	NonSQ PD-L1 < 1%	Original	17.5 (12.3-23.9)	42% (NA-NA)^
		Reconstructed	17.8 (12.6-24.5)	41.2% (33.8-50.4%)
PFS				
POSEIDON	PFS all	Original	6.2 (5-6.5)	NA
		Reconstructed	6.3 (5–6.5)	NA
CM9LA	PFS all	Original	6.7 (5.6-8.0)	9% (NA-NA)
		Reconstructed	6.71 (5.76-8.35)	8.7% (6-12.6%)
MYSTIC	PD-L1 ≥ 25%	Original	3.9 (2.8-5.0)	NA
		Reconstructed	3.9 (2.8-5.54)	NA
CM 227 PART 1	PD-L1 ≥ 1 %	Original	5.1 (4.1-6.3)	12% (NA-NA)
		Reconstructed	5 (4.0–6.5)	12% (8.8–16.3%)

TRIAL	ANALYSIS	SOURCE	MEDIAN OS	5y SURVIVAL
CM 227 PART 1	PD-L1 < 1 %	Original	5.1 (3.5-6.4)	10% (NA-NA)
		Reconstructed	5.0 (3.7-6.9)	9.3% (5.3-16.1%)

^24 months survival
CM = CheckMate; mut = mutated; NA = not available; NonSQ = non-squamous; OS = overall survival; PD-L1 = programmed death-ligand 1; PFS = progression-free survival; SQ = squamous; wt = wild-type.

Supplementary Table 8. Aggregated data network meta-analysis OS results summary

Comparison	HR	CI lower	CI upper	p-value	Source
**OS all patients**					
DICI vs CT	0.79	0.68	0.92	0.003	Direct
DICI-CT vs CT	0.75	0.62	0.9	0.002	Direct
DICI-CT vs DICI	0.95	0.75	1.21	0.667	Indirect
**KEAP1 mutated**					
DICI vs CT	0.79	0.53	1.17	0.243	Direct
DICI-CT vs CT	0.43	0.16	1.15	0.092	Direct
DICI-CT vs DICI	0.54	0.19	1.57	0.26	Indirect
**KEAP1 wild-type**					
DICI vs CT	1.04	0.85	1.26	0.708	Direct
DICI-CT vs CT	0.76	0.64	0.9	0.002	Direct
DICI-CT vs DICI	0.73	0.56	0.95	0.019	Indirect
**STK11 mutated**					
DICI vs CT	0.77	0.55	1.08	0.126	Direct
DICI-CT vs CT	0.72	0.49	1.04	0.078	Direct
DICI-CT vs DICI	0.93	0.57	1.53	0.781	Indirect
**STK11 wild-type**					
DICI vs CT	0.89	0.68	1.17	0.417	Direct
DICI-CT vs CT	0.77	0.58	1.03	0.082	Direct
DICI-CT vs DICI	0.86	0.58	1.29	0.471	Indirect
**PD-L1 ≥ 1%**					
DICI vs CT	0.79	0.68	0.91	0.001	Direct
DICI-CT vs CT	0.73	0.63	0.85	< 0.001	Direct
DICI-CT vs DICI	0.93	0.76	1.14	0.483	Indirect
**PD-L1 < 1%**					
DICI vs CT	0.65	0.48	0.87	0.004	Direct
DICI-CT vs CT	0.72	0.57	0.91	0.006	Direct
DICI-CT vs DICI	1.11	0.76	1.61	0.585	Indirect
**Squamous**					
DICI vs CT	0.63	0.47	0.83	0.001	Direct
DICI-CT vs CT	0.75	0.59	0.94	0.013	Direct
DICI-CT vs DICI	1.19	0.83	1.71	0.347	Indirect
**Nonsquamous**					
DICI vs CT	0.76	0.65	0.89	0.001	Direct
DICI-CT vs CT	0.74	0.64	0.86	< 0.001	Direct
DICI-CT vs DICI	0.97	0.78	1.2	0.777	Indirect

DICI = Dual Immune Checkpoint Inhibitors; CI = Confidence Interval ; CT = Chemotherapy; HR = Hazard Ratio

Supplementary Table 9. Proportional hazard assumption based on the scaled Schoenfeld residuals in each analysis.

Outcome	Proportional hazard assumption based on the scaled Schoenfeld residuals
**OS**	
All patients	5e-04
KEAP1mut	0.5832
KEAP1wt	0.2183
STK11mut	0.0026
STK11wt	0.4851
PD-L1 ≥ 1%	0.0053
PD-L1 < 1%	0.7231
SQ	0.5875
NonSQ	0.0048
**PFS**	
All patients	<0.001

mut = mutated; NA = not available; NonSQ = non-squamous; OS = overall survival; PD-L1 = programmed death-ligand 1; PFS = progression-free survival; SQ = squamous; wt = wild-type.

Supplementary Table 10. Subgroup interaction analysis in the DICI-CT vs DICI comparison

Subgroup	P-interaction
PD-L1 (neg / pos)	0.537
Histology (nonsq / sq)	0.590
STK11 (wt / mut)	0.165
KEAP1 (wt / mut)	0.563

Supplementary Table 11. Summary of treatment effect measures for the DICI-CT versus DICI indirect comparison across all analyses

Analysis	N (DICI-CT/ DICI)	Cox (95% CI)	P	PH p-value	TV-HR t=0 mo	TV-HR t=24 mo	TV-HR t=60 mo	RMST diff, mo (95% CI)	τ, mo	P (RMST)
**OS overall**	699 / 955	0.93 (0.81–1.08)	0.335	0.0005	0.59 (0.43–0.80)*	1.21 (1.05–1.41)*	1.49 (1.18–1.87)*	−0.01 (−0.07 to 0.05)	60	0.815
**PFS overall**	699 / 746	0.90 (0.78–1.03)	0.131	<0.001	0.41 (0.31–0.54)*	1.76 (1.38–2.24)*	2.64 (1.84–3.77)*	−0.01 (−0.05 to 0.04)	60	0.764
**OS PD-L1 ≥1%**	417 / 559	0.93 (0.78–1.12)	0.493	0.005	0.57 (0.38–0.86)*	1.26 (1.04–1.53)*	1.57 (1.16–2.13)*	−0.01 (−0.09 to 0.07)	60	0.855
**OS PD-L1 <1%**	260 / 187	1.12 (0.86–1.45)	0.402	0.723	N/A	N/A	N/A	N/A	—	—
**OS Squamous**	239 / 164	1.18 (0.91–1.54)	0.216	0.588	N/A	N/A	N/A	−0.07 (−0.17 to 0.03)	60	0.178
**OS Non-squamous**	460 / 419	1.06 (0.89–1.27)	0.468	0.005	0.59 (0.38–0.93)*	1.36 (1.11–1.66)*	1.71 (1.23–2.36)*	−0.05 (−0.14 to 0.04)	60	0.269
**OS STK11 mutated**	81 / 51	0.78 (0.46–1.30)	0.339	0.003	0.14 (0.05–0.39)*	1.50 (0.65–3.48)	2.91 (0.82–10.29)	0.43 (0.16–0.71)†	24	0.002
**OS STK11 wild-type**	293 / 271	0.68 (0.56–0.82)	<0.001	0.485	0.59 (0.32–1.09)	0.75 (0.55–1.00)	N/A	0.26 (0.09–0.44)†	24	0.003
**OS KEAP1 mutated**	22 / 63	0.64 (0.36–1.14)	0.129	0.583	N/A	N/A	N/A	N/A	—	—
**OS KEAP1 wild-type**	303 / 259	0.75 (0.60–0.95)	0.016	0.218	N/A	N/A	N/A	0.14 (−0.03 to 0.31)	24	0.113

HR <1 favours DICI-CT. HR >1 favours DICI. All HRs are indirect estimates from the mixed-effects Cox ITC (coxme, random intercept by trial, CT = reference).* p<0.05. N/A = analysis not performed (PH assumption not violated or insufficient follow-up data). Cox PH = proportional hazards model. TV-HR = time-varying coefficient Cox model with log-time interaction. RMST = restricted mean survival time.

†For STK11 subgroups, RMST difference at τ=24 months is reported because mature data beyond 24 months were limited; positive difference indicates DICI-CT favoured at this horizon. For all other analyses, τ=60 months was prespecified; negative difference indicates DICI-CT favoured.

Supplementary Figure 1. Reconstructed Kaplan-Meier plots of individual studies

A) CheckMate 227 Part 1

A) OS PD-L1 ≥ 1%

Treatment	N	Events	Median OS, mc (95% CI)	2−yr OS (95% CI)	3−yr OS (95% CI)	5−yr OS (95% CI)
CT	397	328	15 (13–16.7)	33.3% (28.9–38.3%)	22% (18.2–26.6%)	14.4% (11.2–18.5%)
DICI	396	294	17.1 (15.2–20.3)	39.7% (35–44.9%)	32.5% (28.1–37.5%)	23.9% (20–28.6%)

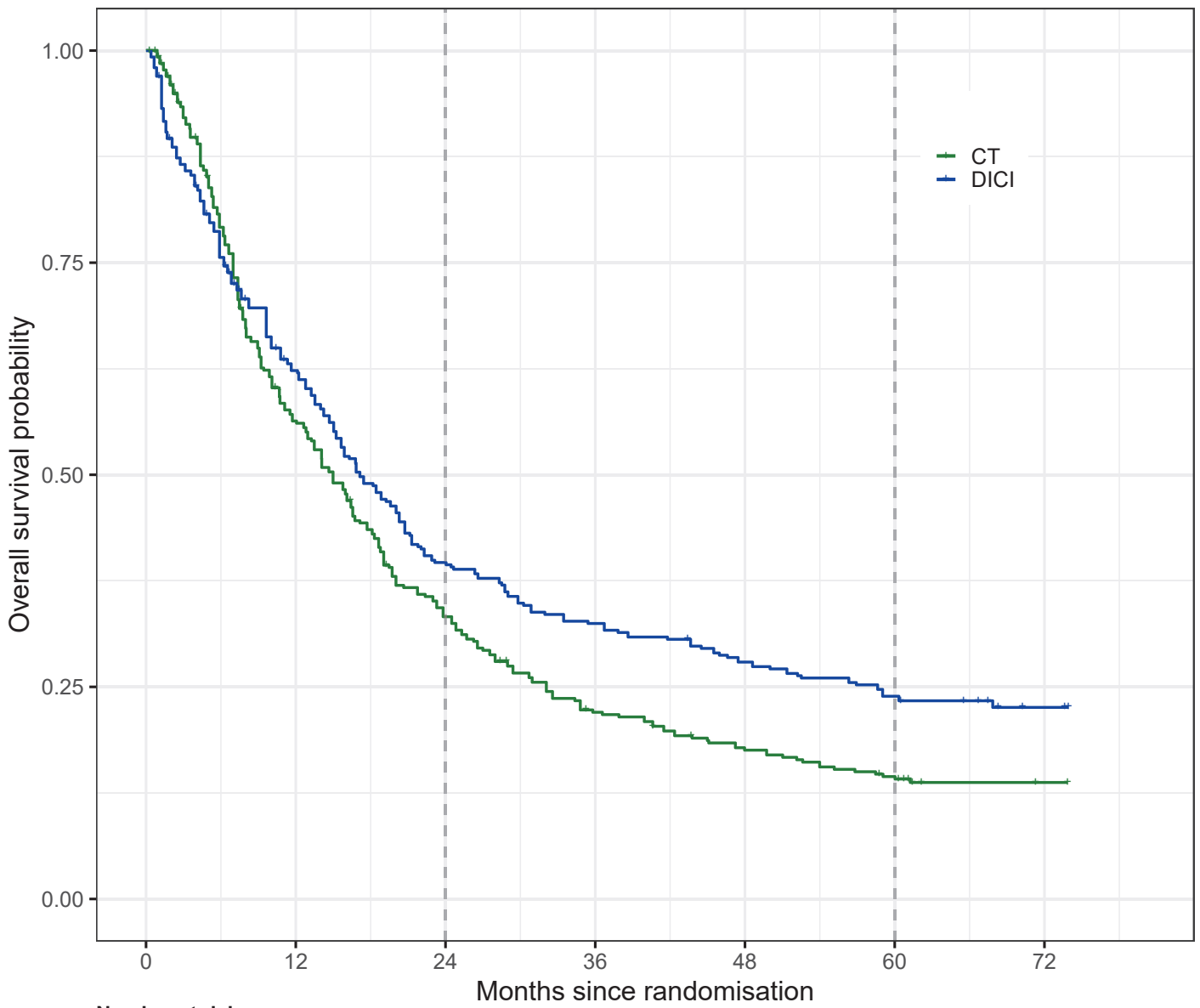

Number at risk

	0	12	24	36	48	60	72
CT	397	216	126	80	62	50	2
DICI	396	234	149	122	104	89	3

B) OS PD-L1 < 1%

Treatment	N	Events	Median OS, mc (95% CI)	2−yr OS (95% CI)	3−yr OS (95% CI)	5−yr OS (95% CI)
CT	186	171	12.2 (9.2–14.7)	23% (17.6–30%)	15.2% (10.7–21.4%)	7% (4.1–12%)
DICI	187	151	17.5 (13.4–23.7)	40.9% (34.3–48.6%)	33.1% (27–40.7%)	18.9% (13.9–25.6%)

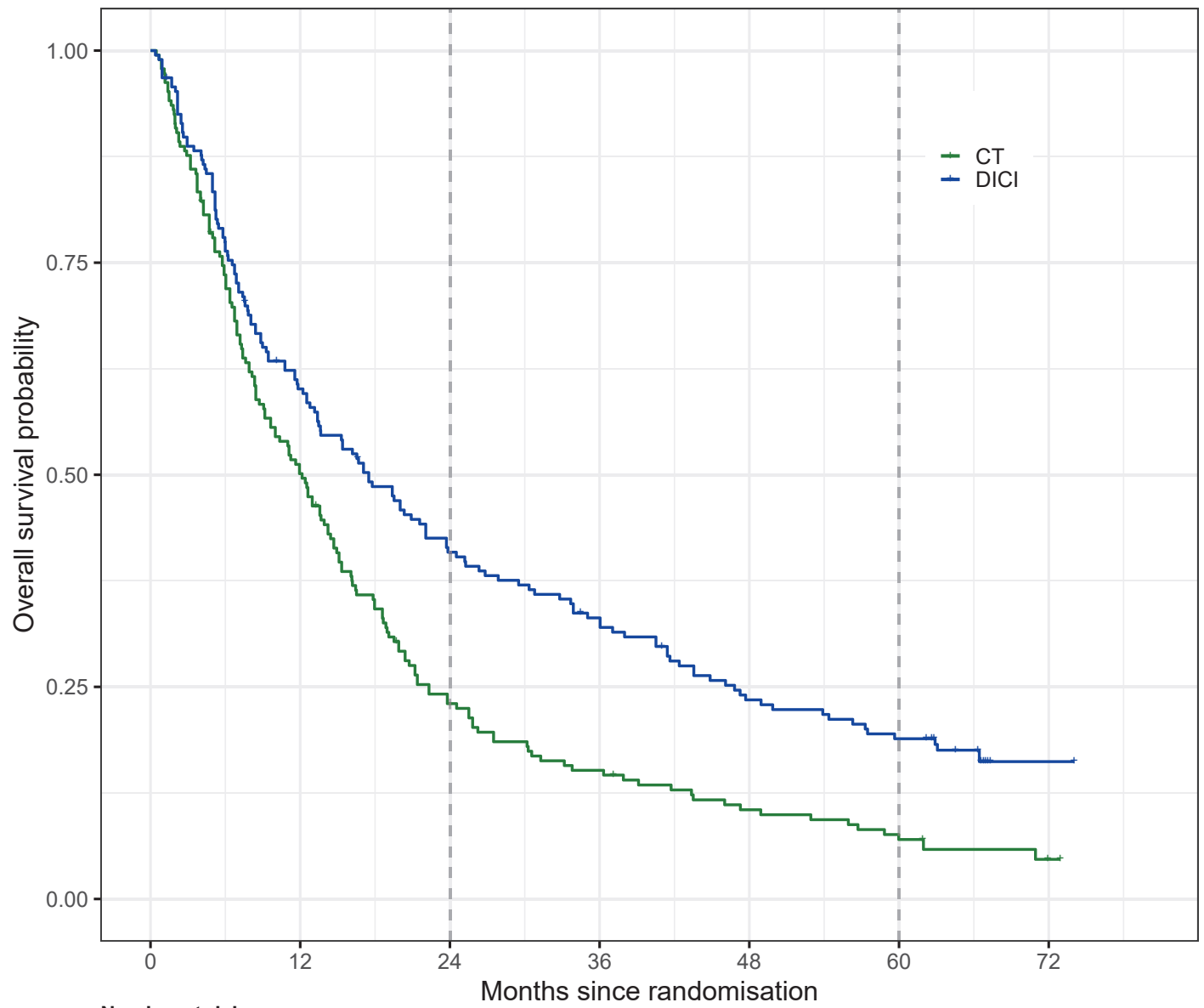

Number at risk

	0	12	24	36	48	60	72
CT	186	92	41	27	18	12	1
DICI	187	110	74	59	41	33	3

C) PFS PD-L1 ≥ 1%

Treatment	N	Events	Median PFS, m (95% CI)	2−yr PFS (95% CI)	3−yr PFS (95% CI)	5−yr PFS (95% CI)
CT	397	292	5.5 (4.6–5.7)	7.4% (4.7–11.6%)	4.6% (2.5–8.6%)	2.3% (0.9–5.9%)
DICI	396	309	5 (4–6.6)	22.5% (18.5–27.4%)	18.7% (14.9–23.4%)	12% (8.8–16.3%)

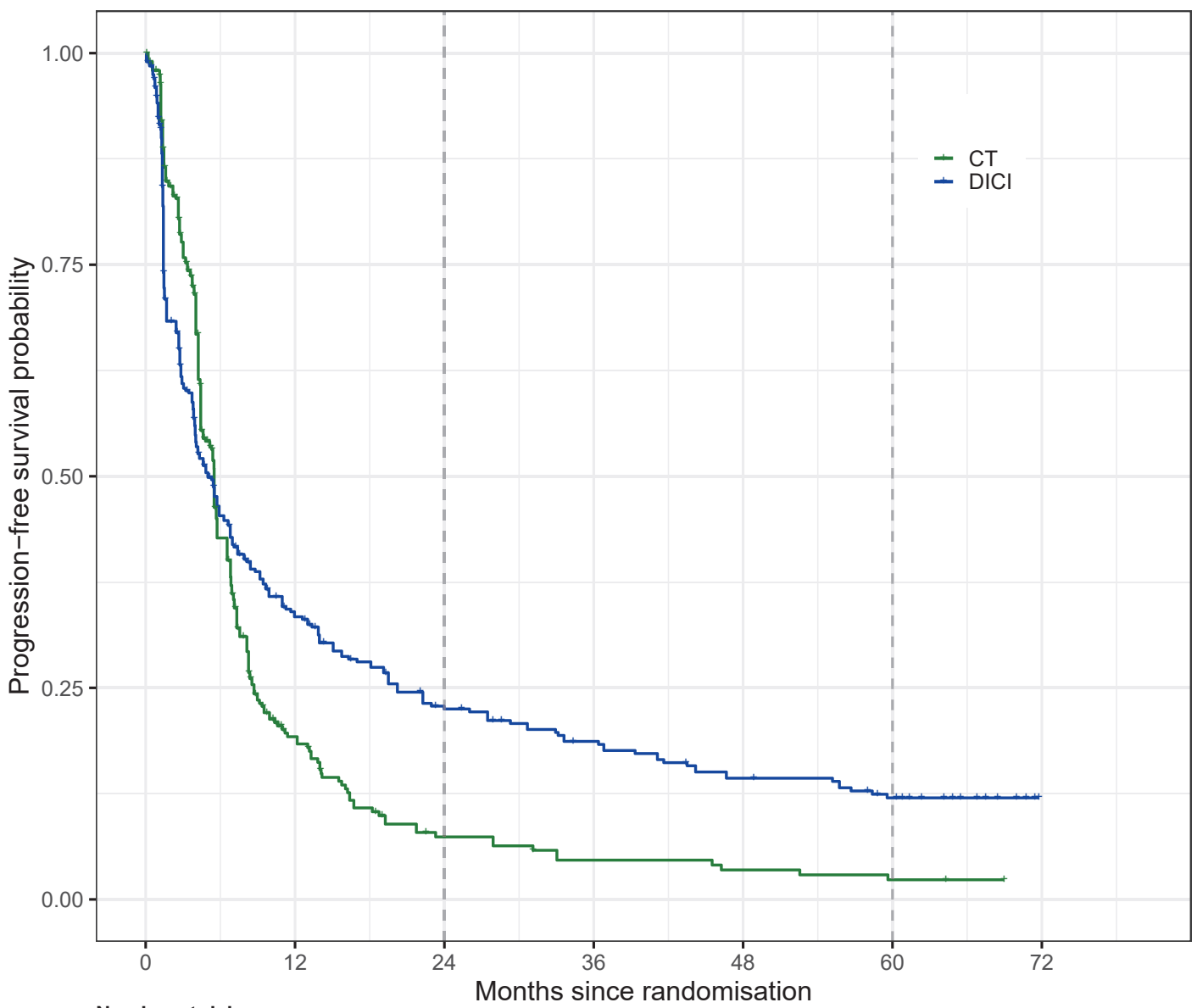

Number at risk

	0	12	24	36	48	60	72
CT	397	45	14	8	6	4	0
DICI	396	111	67	52	39	28	0

D) PFS PD-L1 < 1%

Treatment	N	Events	Median PFS, m (95% CI)	2−yr PFS (95% CI)	3−yr PFS (95% CI)	5−yr PFS (95% CI)
CT	186	153	5 (4.1–5.5)	5% (2.4–10.7%)	2.5% (0.8–7.6%)	1.7% (0.4–6.5%)
DICI	187	140	5 (3.7–6.9)	17.8% (12.5–25.3%)	14% (9.3–21.1%)	9.3% (5.3–16.1%)

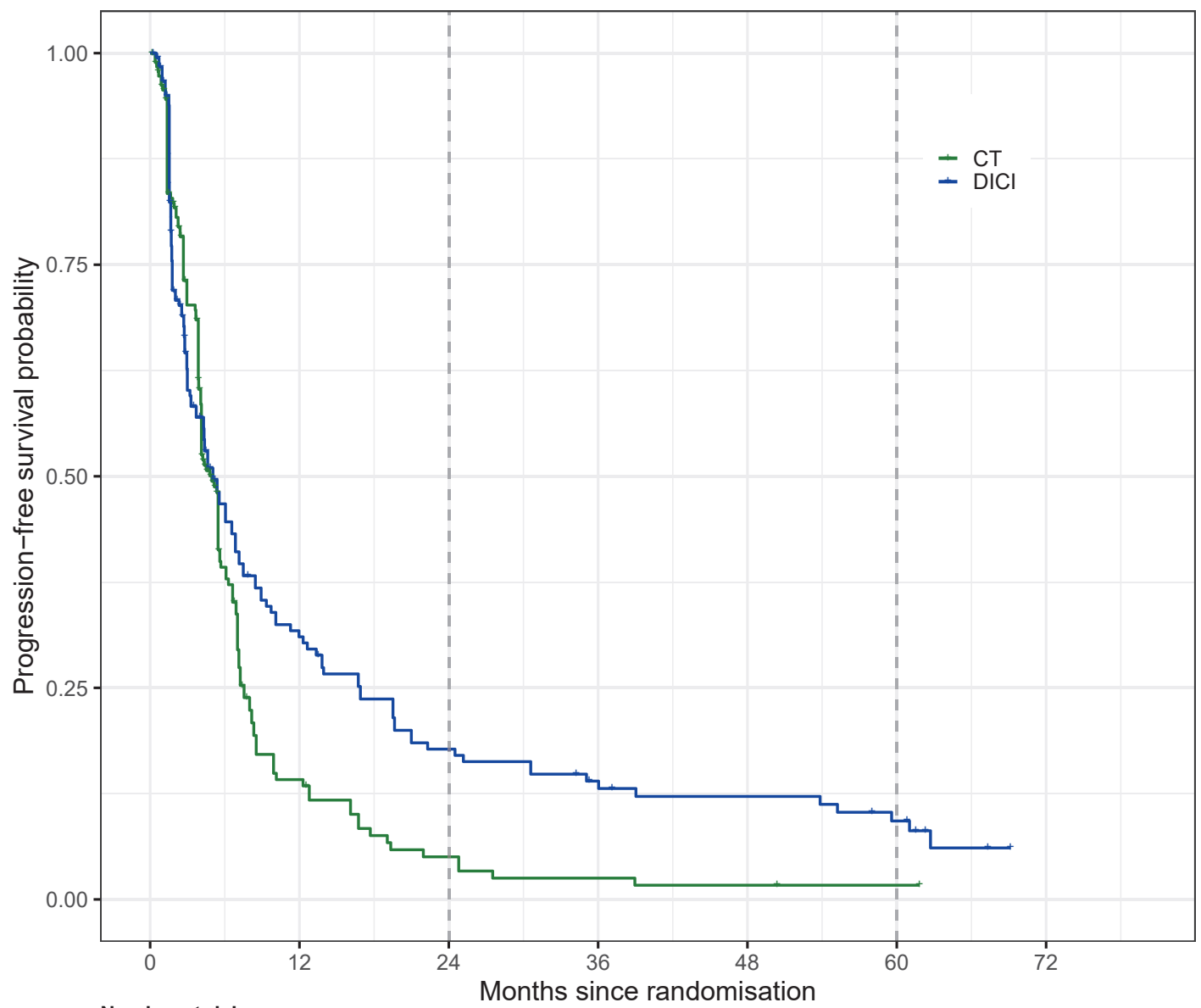

Number at risk

	0	12	24	36	48	60	72
CT	186	19	6	3	2	1	0
DICI	187	43	24	16	13	9	0

E) OS SQUAMOUS PD-L1 ≥ 1%

Treatment	N	Events	Median OS, mc (95% CI)	2−yr OS (95% CI)	3−yr OS (95% CI)	5−yr OS (95% CI)
CT	118	107	9.2 (7.8–14.1)	20% (13.9–28.8%)	9.1% (5–16.6%)	5% (2.2–11.7%)
DICI	118	92	14.9 (12.4–19.3)	29.7% (22.3–39.4%)	24.3% (17.5–33.6%)	19.6% (13.4–28.5%)

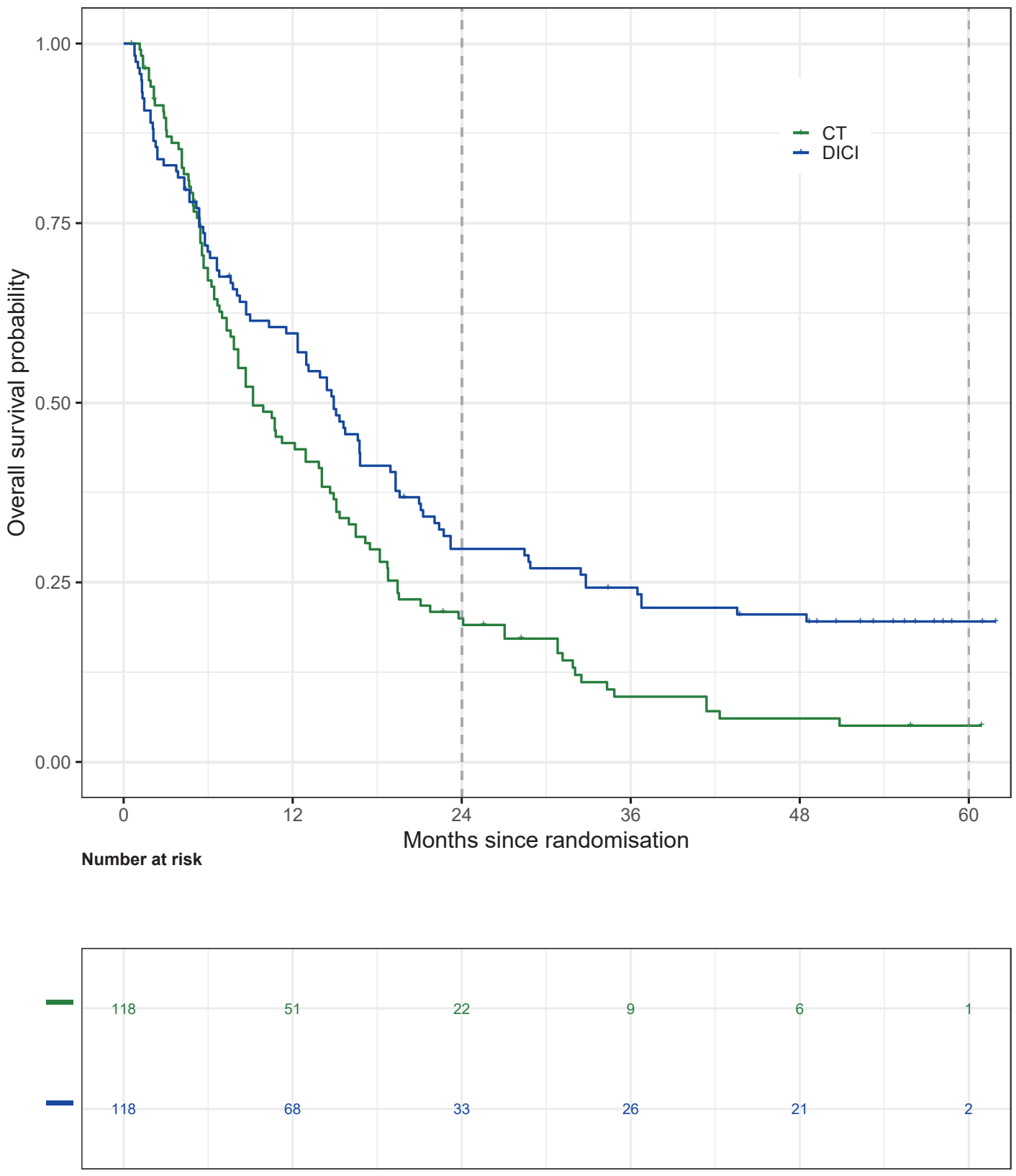

F) OS SQUAMOUS PD-L1 < 1%

Treatment	N	Events	Median OS, mc (95% CI)	2−yr OS (95% CI)	3−yr OS (95% CI)	5−yr OS (95% CI)
CT	46	41	8.6 (6.9–15.2)	14.6% (7–30.1%)	8.7% (3.1–24.2%)	NR
DICI	46	36	15.6 (11.7–37.2)	37.2% (25.3–54.6%)	34.8% (23.2–52.2%)	NR

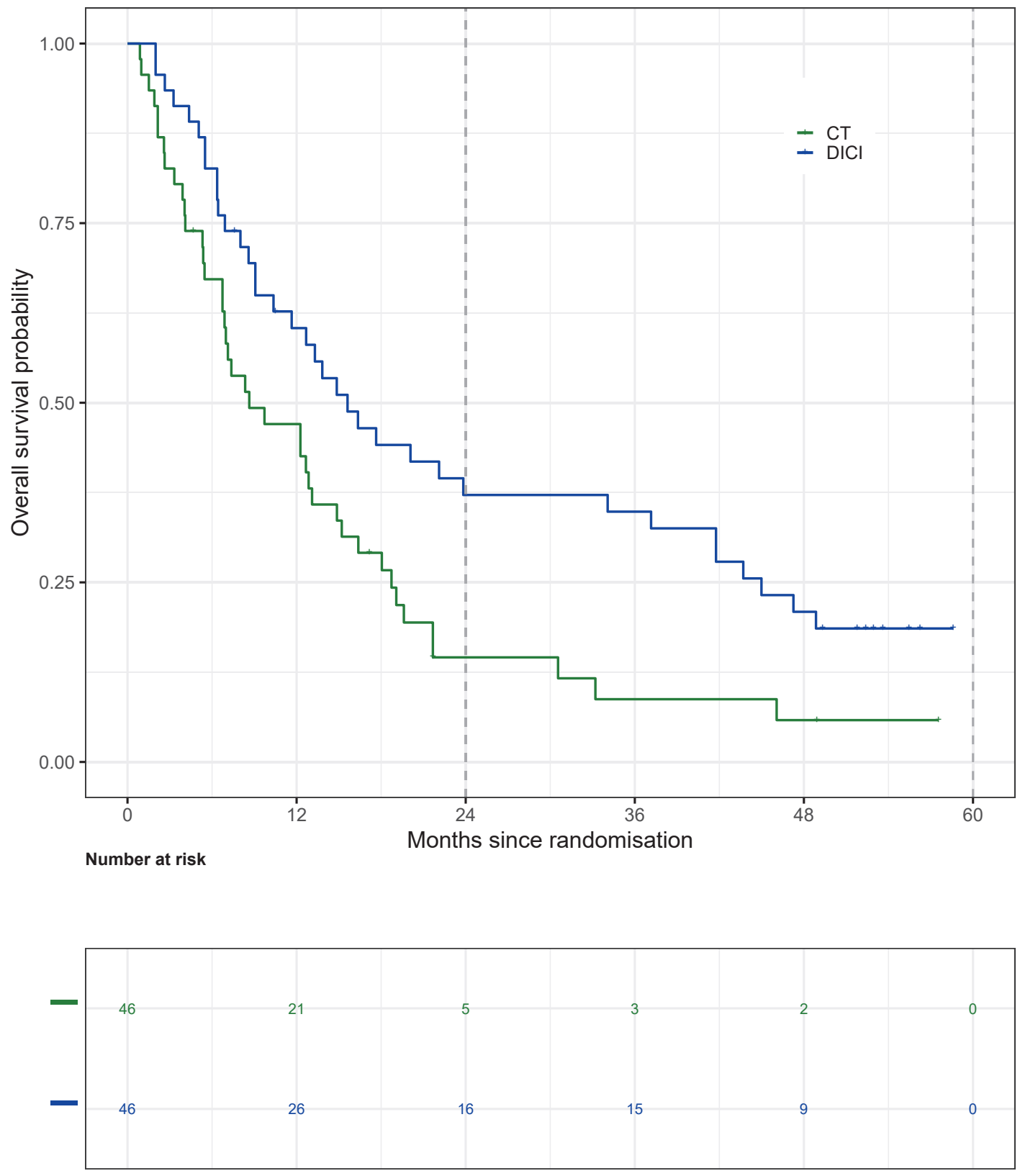

G) OS NONSQUAMOUS PD-L1 ≥ 1%

Treatment	N	Events	Median OS, mc (95% CI)	2−yr OS (95% CI)	3−yr OS (95% CI)	5−yr OS (95% CI)
CT	279	215	17.2 (14.6–19.9)	39.3% (33.9–45.6%)	27.9% (23–33.8%)	18.2% (13.8–24.1%)
DICI	278	189	19.4 (15.7–24.6)	43.7% (38.2–50.1%)	36.5% (31.2–42.7%)	29.5% (24.4–35.8%)

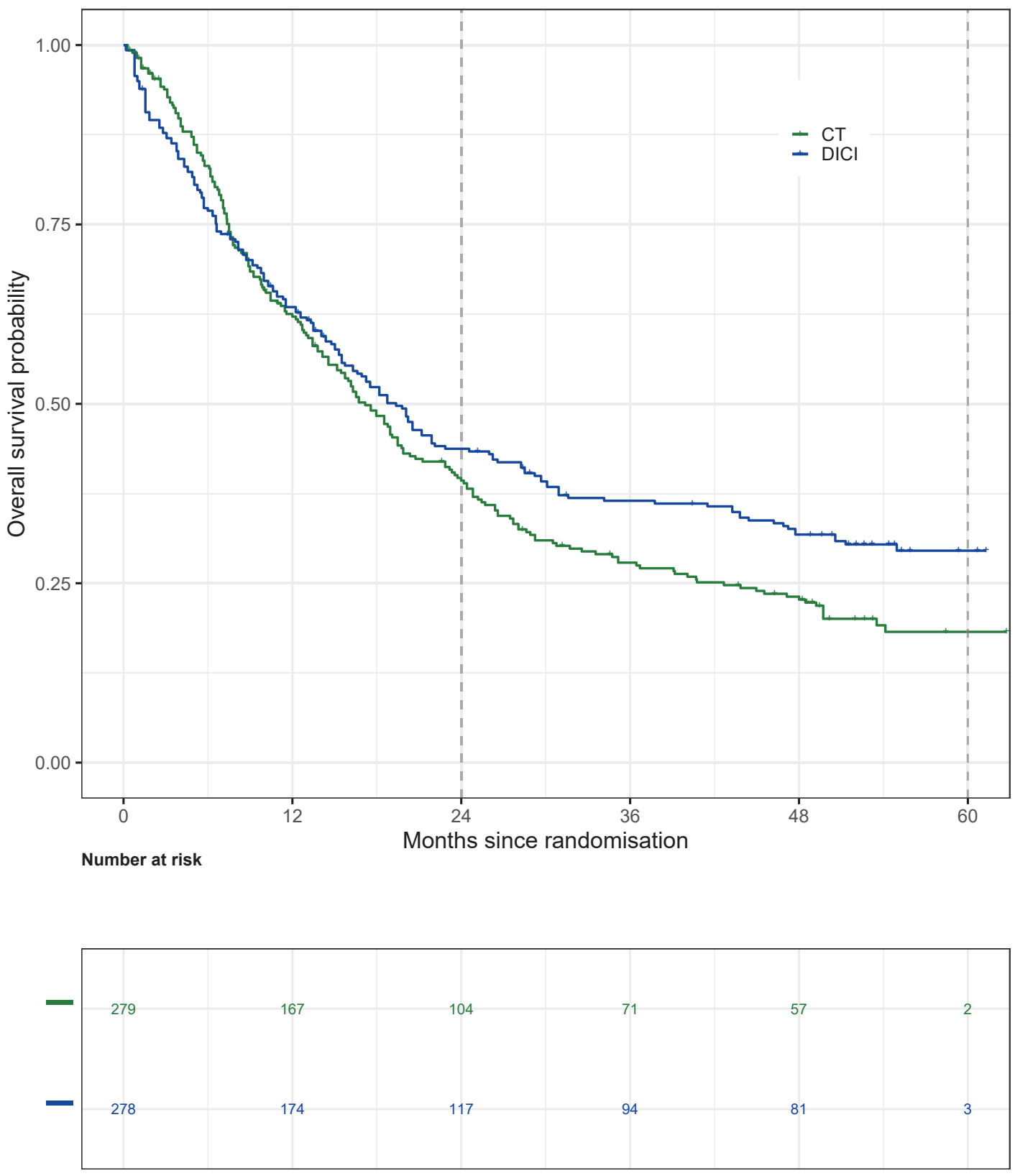

H) OS NONSQUAMOUS PD-L1 < 1%

Treatment	N	Events	Median OS, mc (95% CI)	2−yr OS (95% CI)	3−yr OS (95% CI)	5−yr OS (95% CI)
CT	140	123	13.1 (10.1–15.3)	25.2% (18.9–33.6%)	17% (11.8–24.7%)	9.2% (5.2–16.2%)
DICI	141	105	17.8 (12.6–24.5)	41.2% (33.8–50.4%)	33.7% (26.6–42.7%)	21% (14.7–30%)

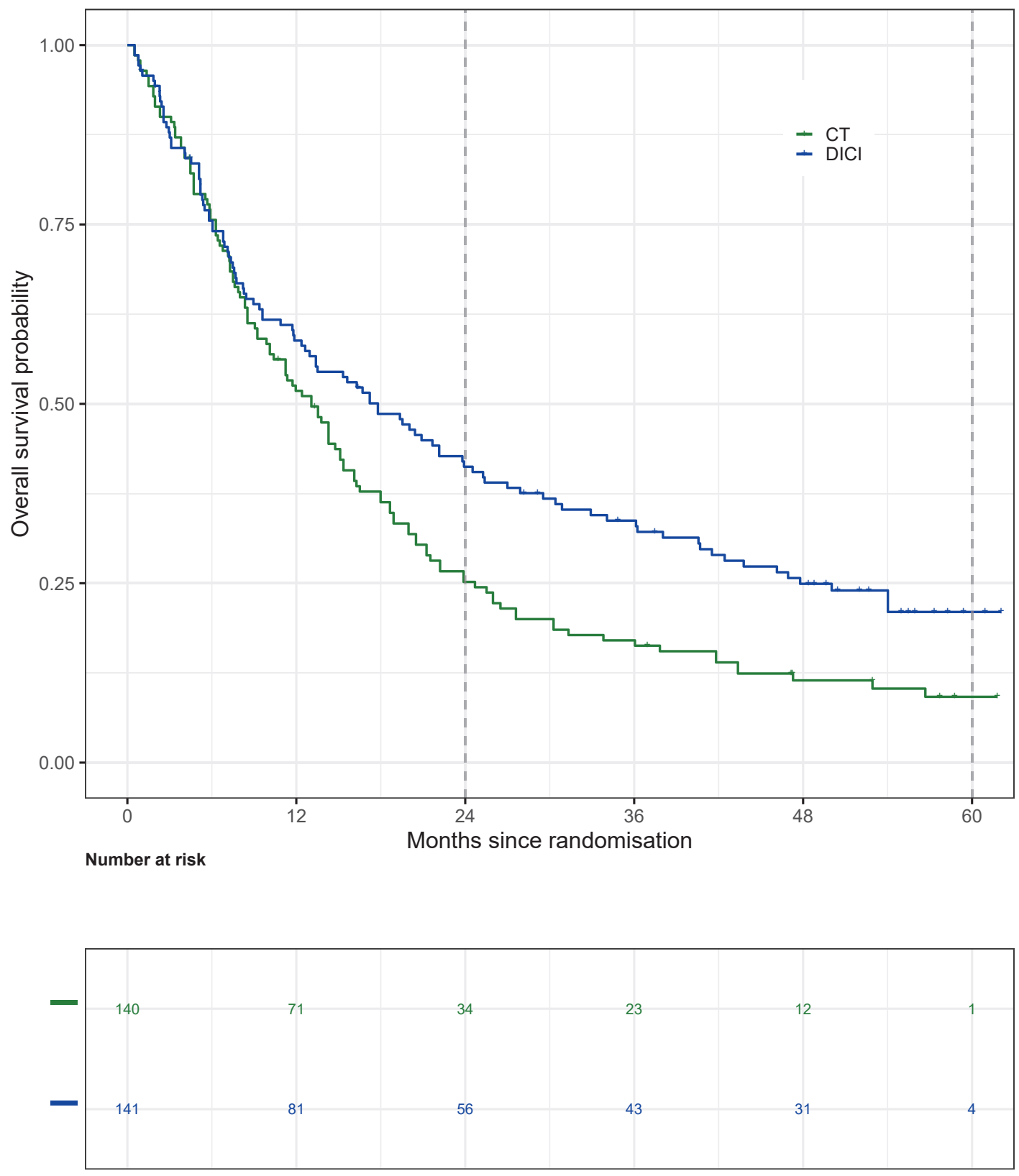

B) CheckMate 9LA

A) OS all patients

Treatment	N	Events	Median OS, mc (95% CI)	2−yr OS (95% CI)	3−yr OS (95% CI)	5−yr OS (95% CI)
CT	358	315	11.3 (10–13.7)	26.8% (22.5–31.9%)	19.1% (15.3–23.7%)	10.8% (7.9–14.7%)
DICI−CT	361	306	15.7 (13.8–19.7)	38.3% (33.5–43.6%)	26.7% (22.5–31.7%)	18.4% (14.8–22.9%)

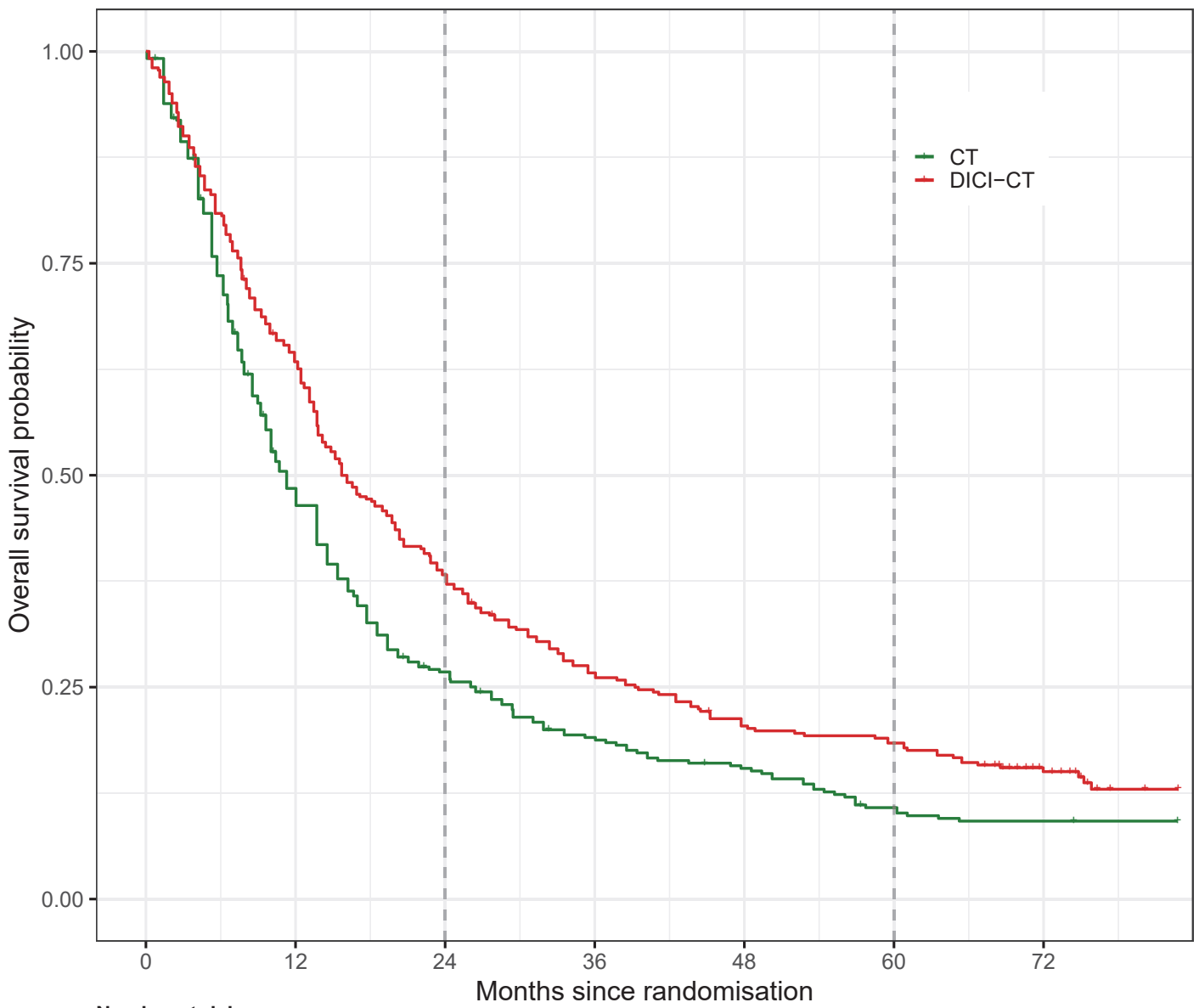

Number at risk

	0	12	24	36	48	60	72
CT	358	168	91	63	50	34	29
DICI−CT	361	227	137	94	71	64	33

B) PFS all patients

Treatment	N	Events	Median PFS, m (95% CI)	2−yr PFS (95% CI)	3−yr PFS (95% CI)	5−yr PFS (95% CI)
CT	358	287	5.2 (4.6–5.7)	8.7% (5.9–12.9%)	5.3% (3.1–8.9%)	4.3% (2.4–7.8%)
DICI−CT	361	298	6.7 (5.8–8.4)	19.3% (15.4–24.2%)	14.1% (10.7–18.6%)	8.7% (6–12.6%)

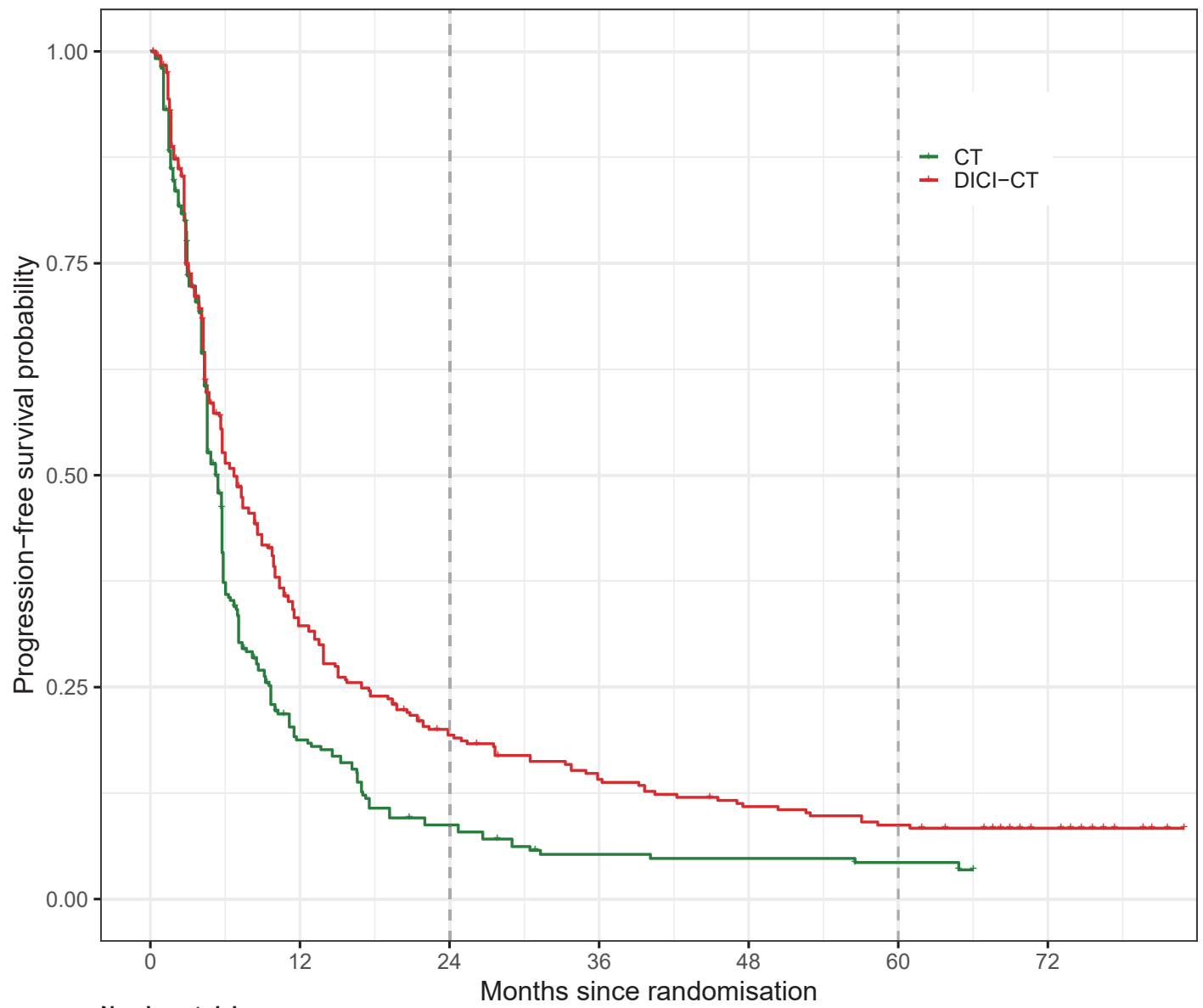

Number at risk

	0	12	24	36	48	60	72
CT	358	49	21	11	10	5	0
DICI−CT	361	101	57	40	30	24	10

C) OS PD-L1 ≥ 1%

Treatment	N	Events	Median OS, mc (95% CI)	2−yr OS (95% CI)	3−yr OS (95% CI)	5−yr OS (95% CI)
CT	204	179	10.6 (9.7–13.2)	27.9% (22.4–34.9%)	19.2% (14.4–25.5%)	10.8% (7.1–16.3%)
DICI−CT	204	173	15.7 (13.8–22.9)	41% (34.7–48.3%)	28.5% (22.9–35.5%)	17.7% (13.1–23.9%)

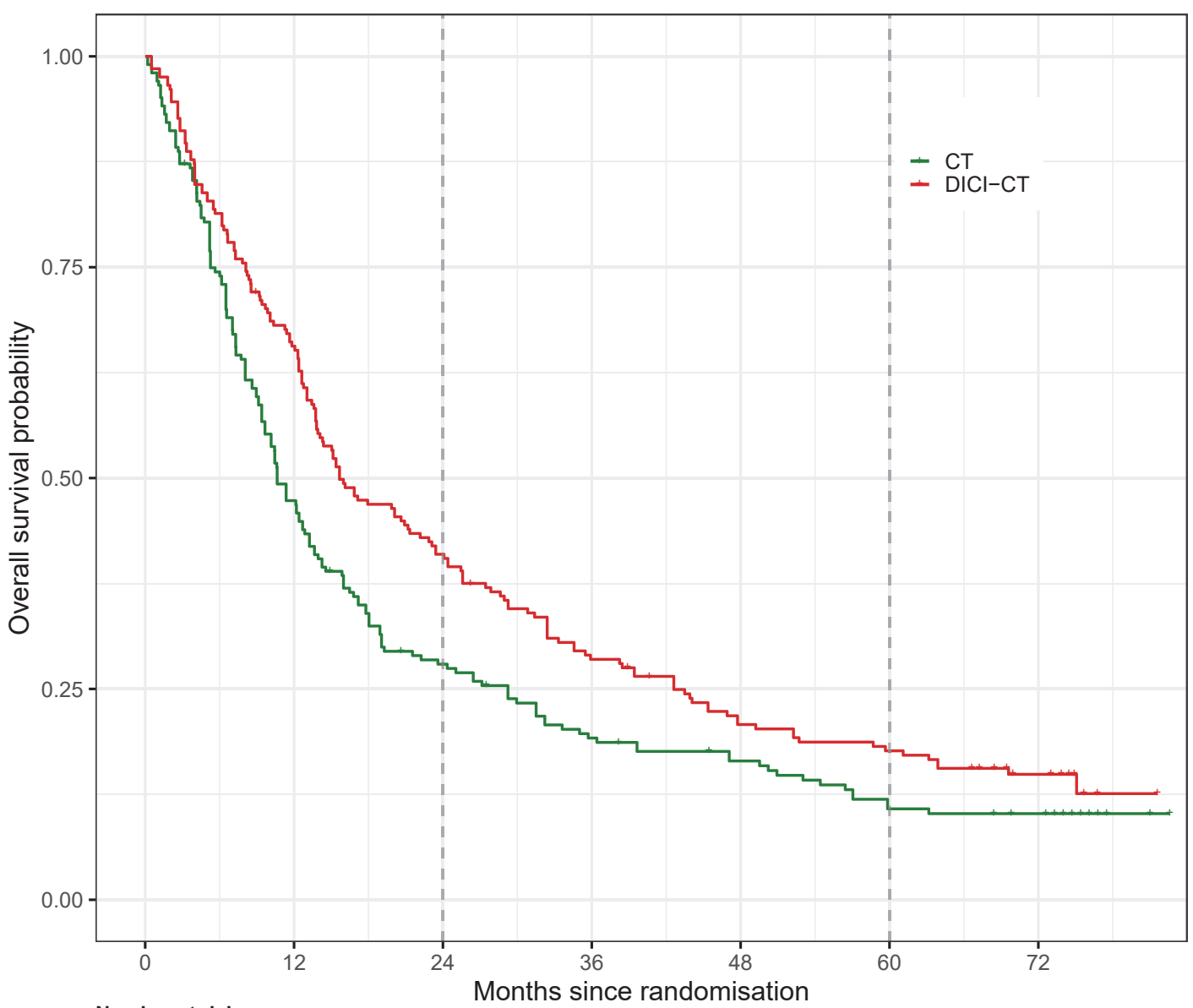

Number at risk

	0	12	24	36	48	60	72
CT	204	96	55	37	29	19	16
DICI−CT	204	133	83	57	40	34	20

D) OS PD-L1 < 1%

Treatment	N	Events	Median OS, mc (95% CI)	2−yr OS (95% CI)	3−yr OS (95% CI)	5−yr OS (95% CI)
CT	129	117	10 (7.9–13.7)	21.9% (15.8–30.5%)	15.3% (10.1–23.1%)	8.1% (4.4–14.9%)
DICI−CT	135	108	17.9 (14.1–22)	37.9% (30.4–47.1%)	25.6% (19.1–34.2%)	21.7% (15.7–30%)

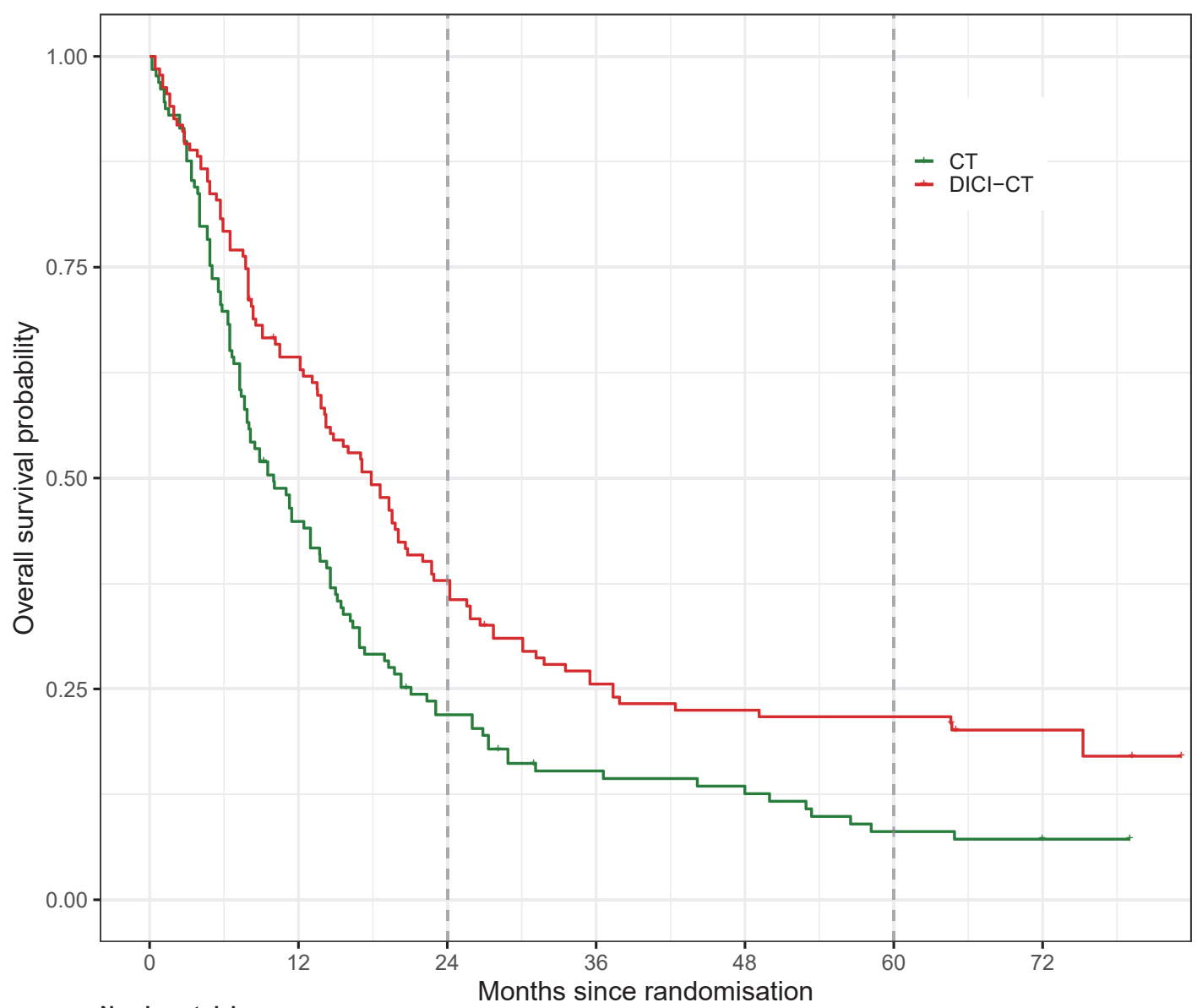

Number at risk

	0	12	24	36	48	60	72
CT	129	57	27	17	14	9	1
DICI−CT	135	85	50	33	29	28	13

E) OS SQUAMOUS

Treatment	N	Events	Median OS, mc (95% CI)	2−yr OS (95% CI)	3−yr OS (95% CI)	5−yr OS (95% CI)
CT	112	105	9.2 (7.4–12.4)	20.2% (13.9–29.2%)	11% (6.5–18.7%)	6.4% (3.1–13.1%)
DICI−CT	115	99	14.6 (13.6–19.8)	35.2% (27.5–45.2%)	24.7% (17.9–34%)	18.3% (12.4–27%)

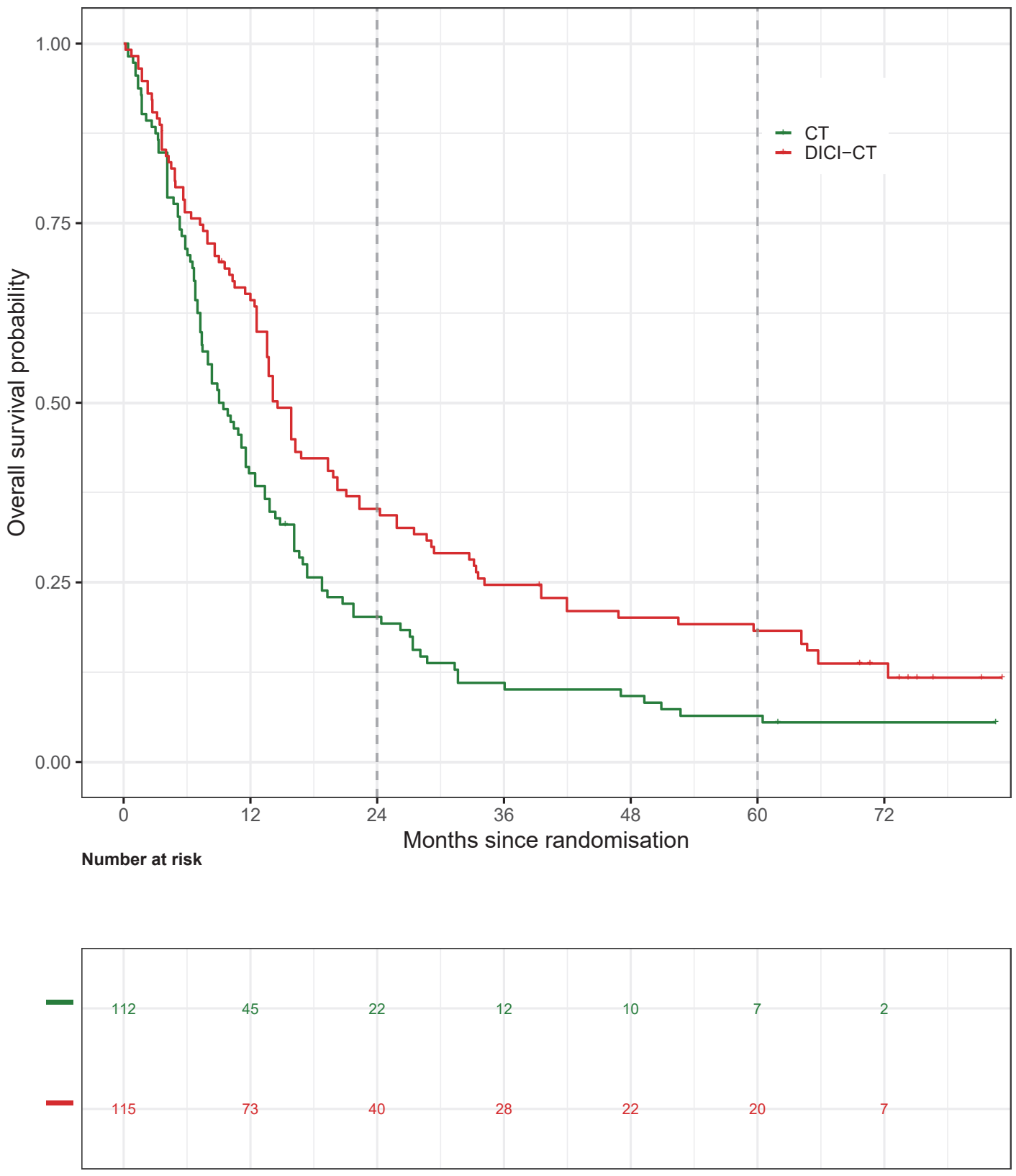

F) OS NONSQUAMOUS

Treatment	N	Events	Median OS, mc (95% CI)	2−yr OS (95% CI)	3−yr OS (95% CI)	5−yr OS (95% CI)
CT	246	210	12 (10–14.1)	29% (23.8–35.3%)	22.9% (18.1–28.9%)	12.6% (8.9–17.8%)
DICI−CT	246	203	17.6 (14.4–22.3)	40.8% (35–47.5%)	27.9% (22.8–34.1%)	19.1% (14.7–24.7%)

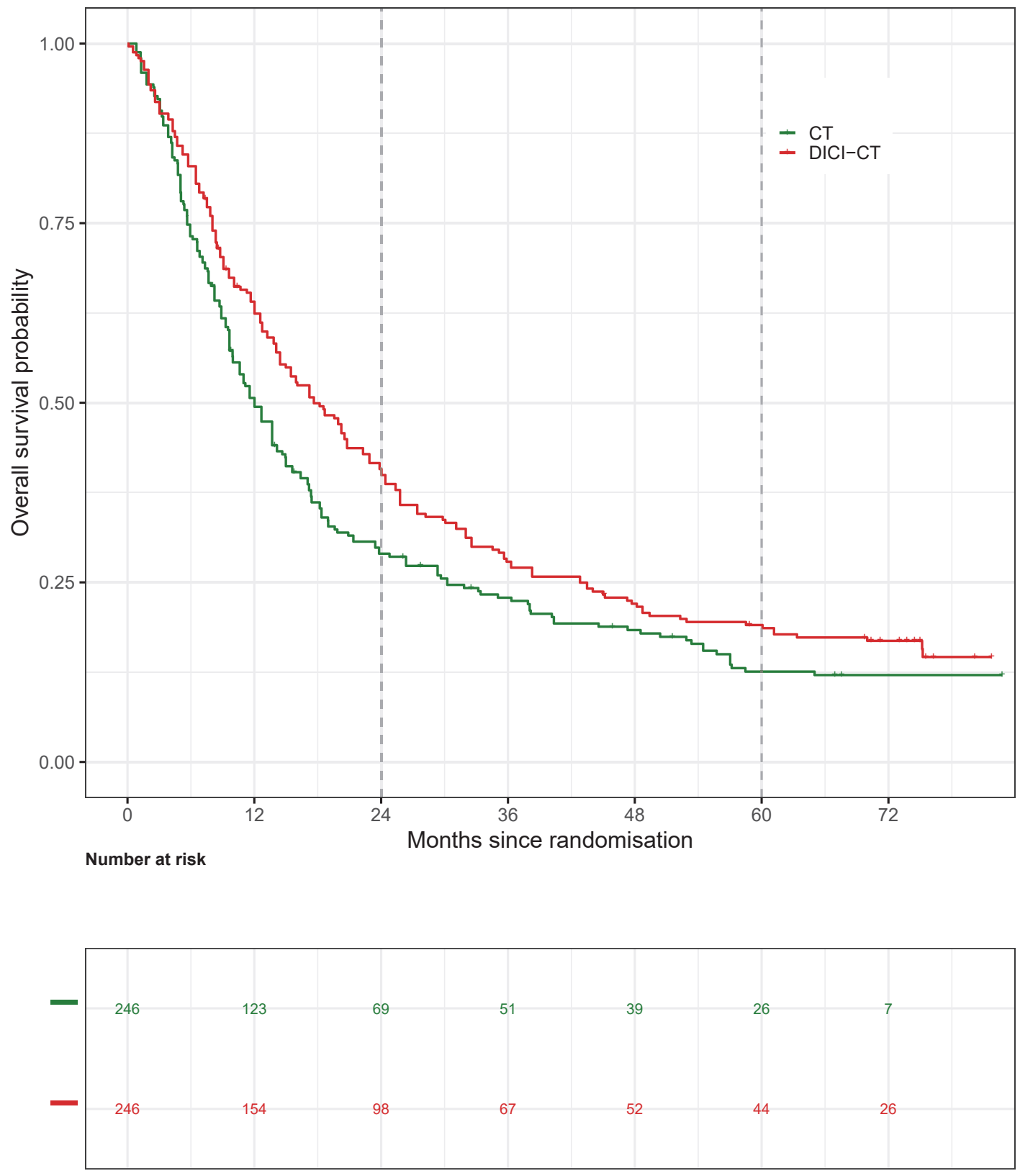

G) OS STK11 mutated

Treatment	N	Events	Median OS, mo (95% CI)	2−yr OS (95% CI)	5−yr OS (95% CI)
CT	36	30	10.6 (7.4–19)	24.4% (13.7–43.7%)	15.3% (6.9–33.7%)
DICI−CT	50	42	13.8 (9.5–23.6)	30% (19.6–45.8%)	19.4% (10.9–34.4%)

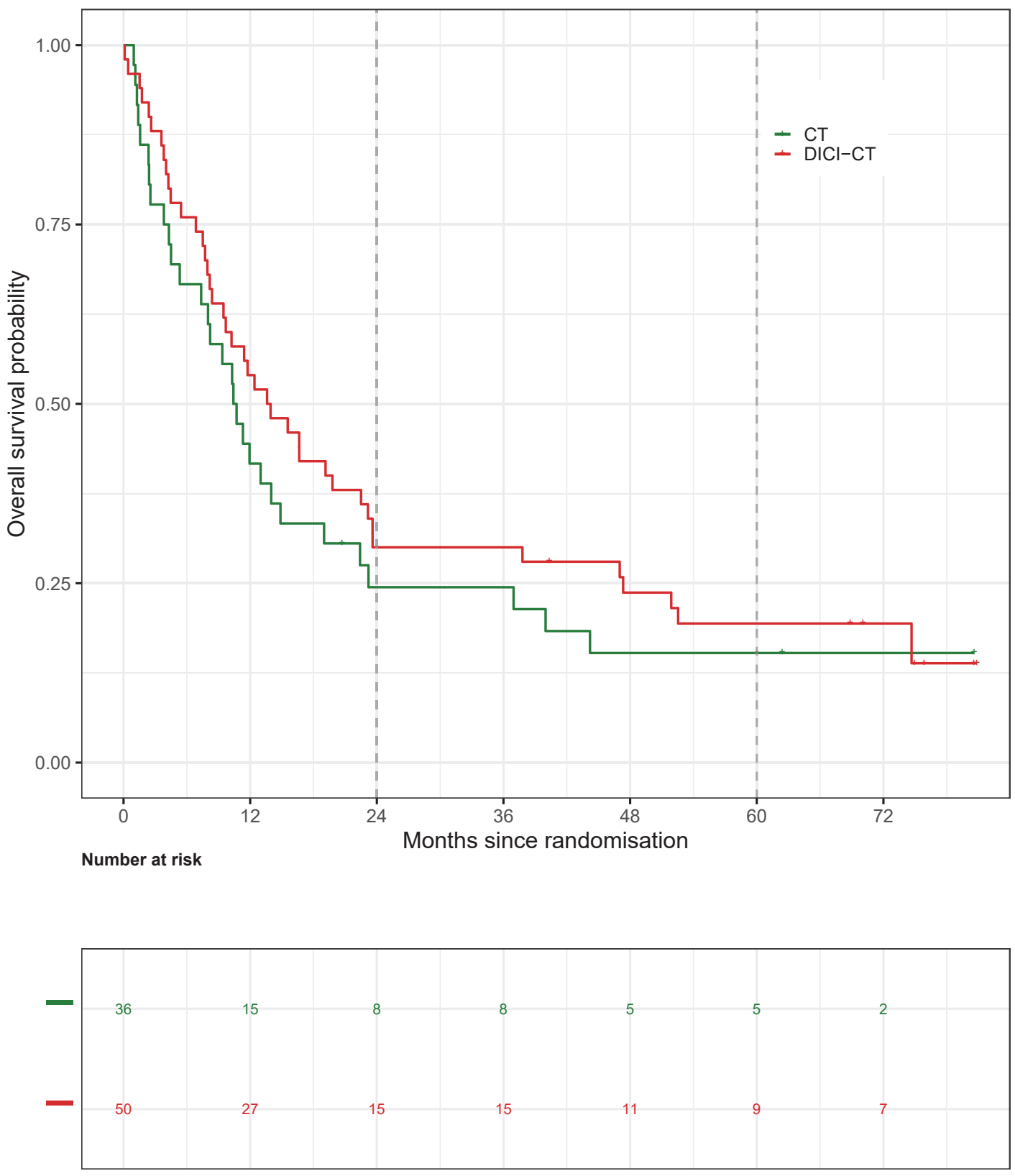

H) OS STK11 wild-type

Treatment	N	Events	Median OS, mo (95% CI)	2−yr OS (95% CI)	5−yr OS (95% CI)
CT	111	92	14.1 (11.5–18.1)	36% (28.1–46.2%)	14.3% (8.8–23.2%)
DICI−CT	116	93	17.9 (14–22.9)	38.5% (30.6–48.5%)	21.9% (15.5–30.9%)

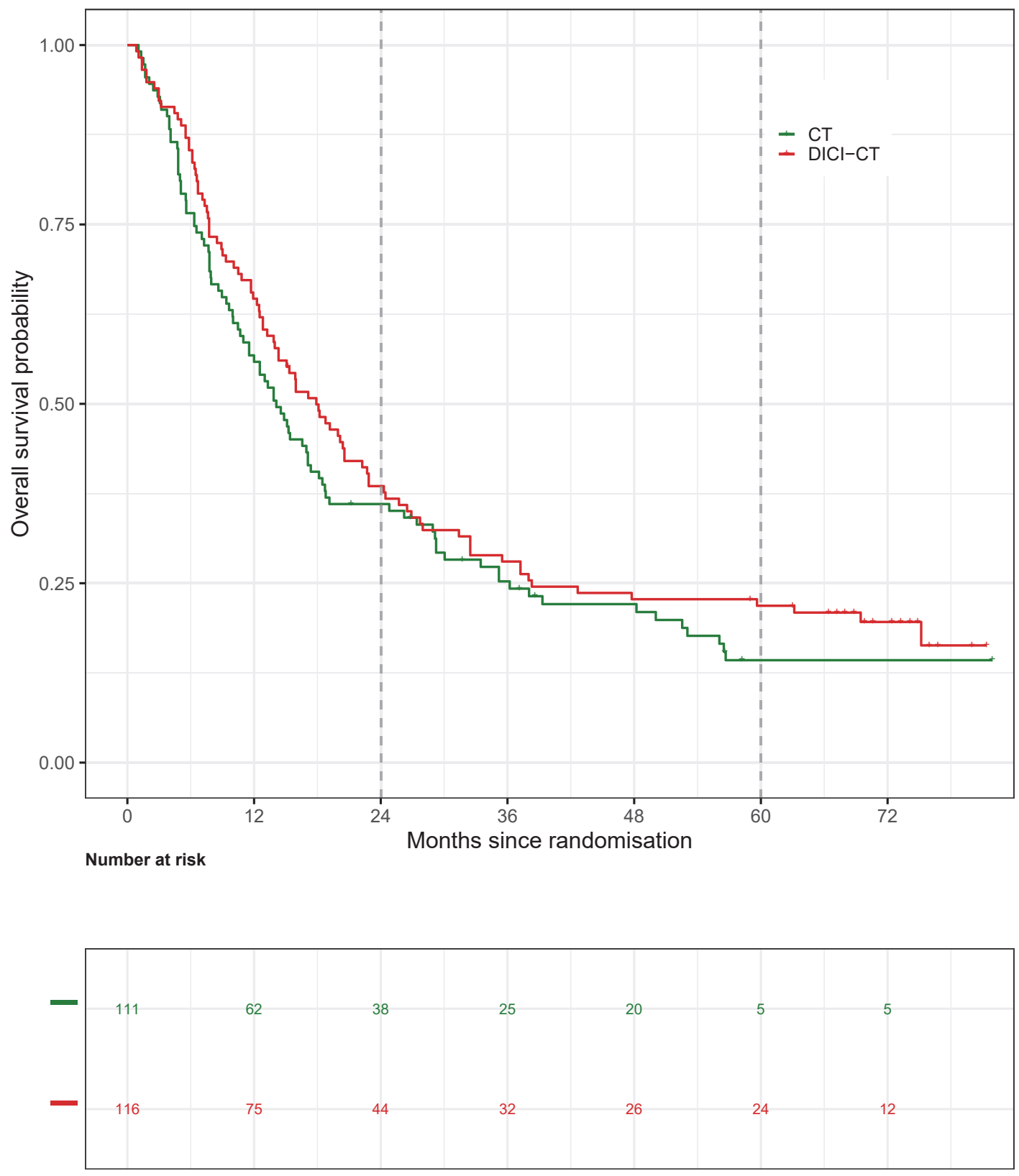

C) MYSTIC

A) OS all patients

Treatment	N	Events	Median OS, mc (95% CI)	2−yr OS (95% CI)	3−yr OS (95% CI)	5−yr OS (95% CI)
CT	372	290	12 (10.6–13.6)	24.7% (20.6–29.7%)	NR	NR
DICI	372	280	11.2 (9.6–12.9)	29% (24.7–34%)	18.4% (12.9–26.2%)	NR

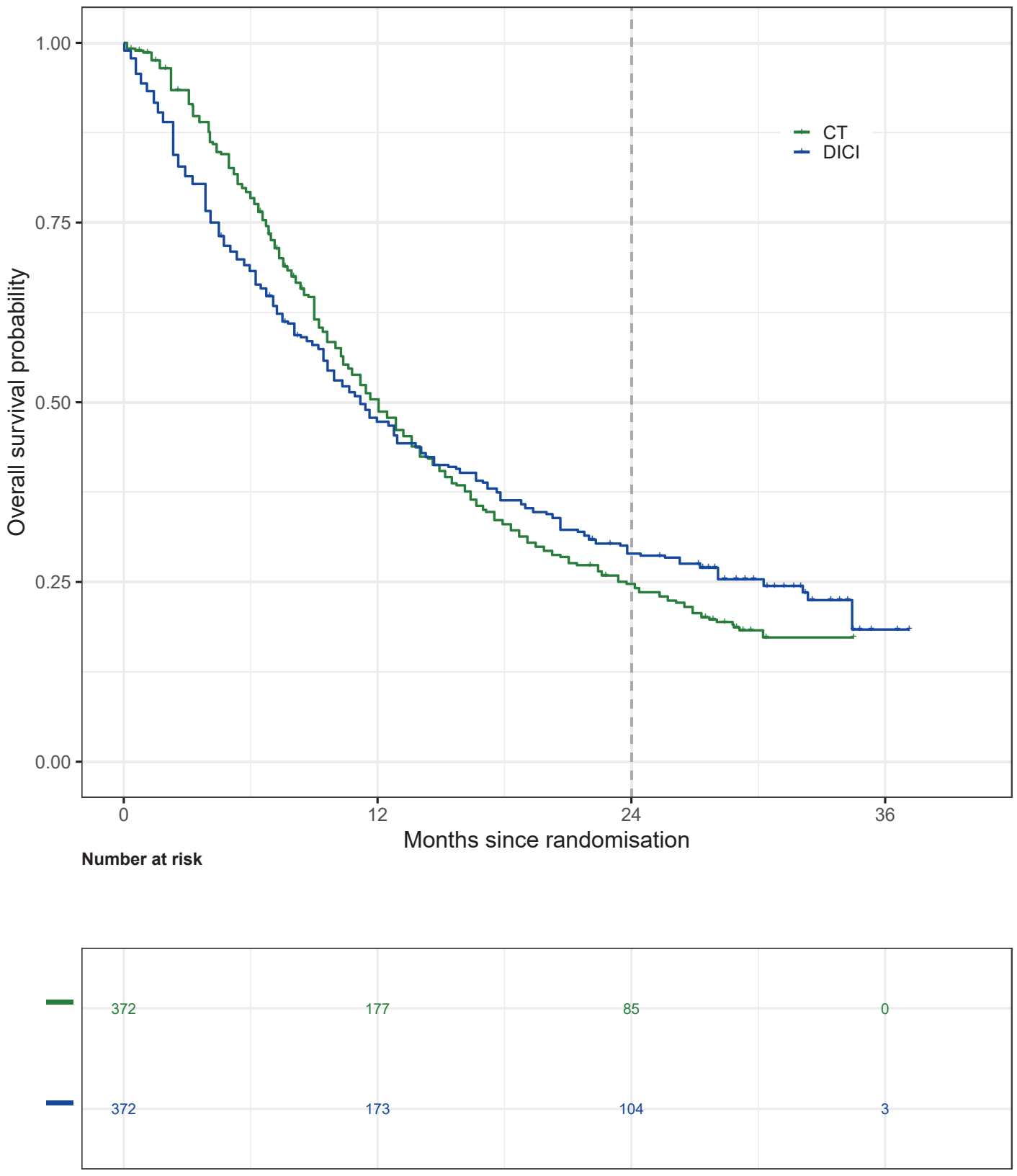

B) PFS all patients

Treatment	N	Events	Median PFS, mo (95% CI)	1−yr PFS (95% CI)	2−yr PFS (95% CI)
CT	162	110	5.4 (4.8–6.2)	15.3% (9.7–24.2%)	NR
DICI	163	117	3.9 (2.8–5.5)	26% (19.7–34.3%)	NR

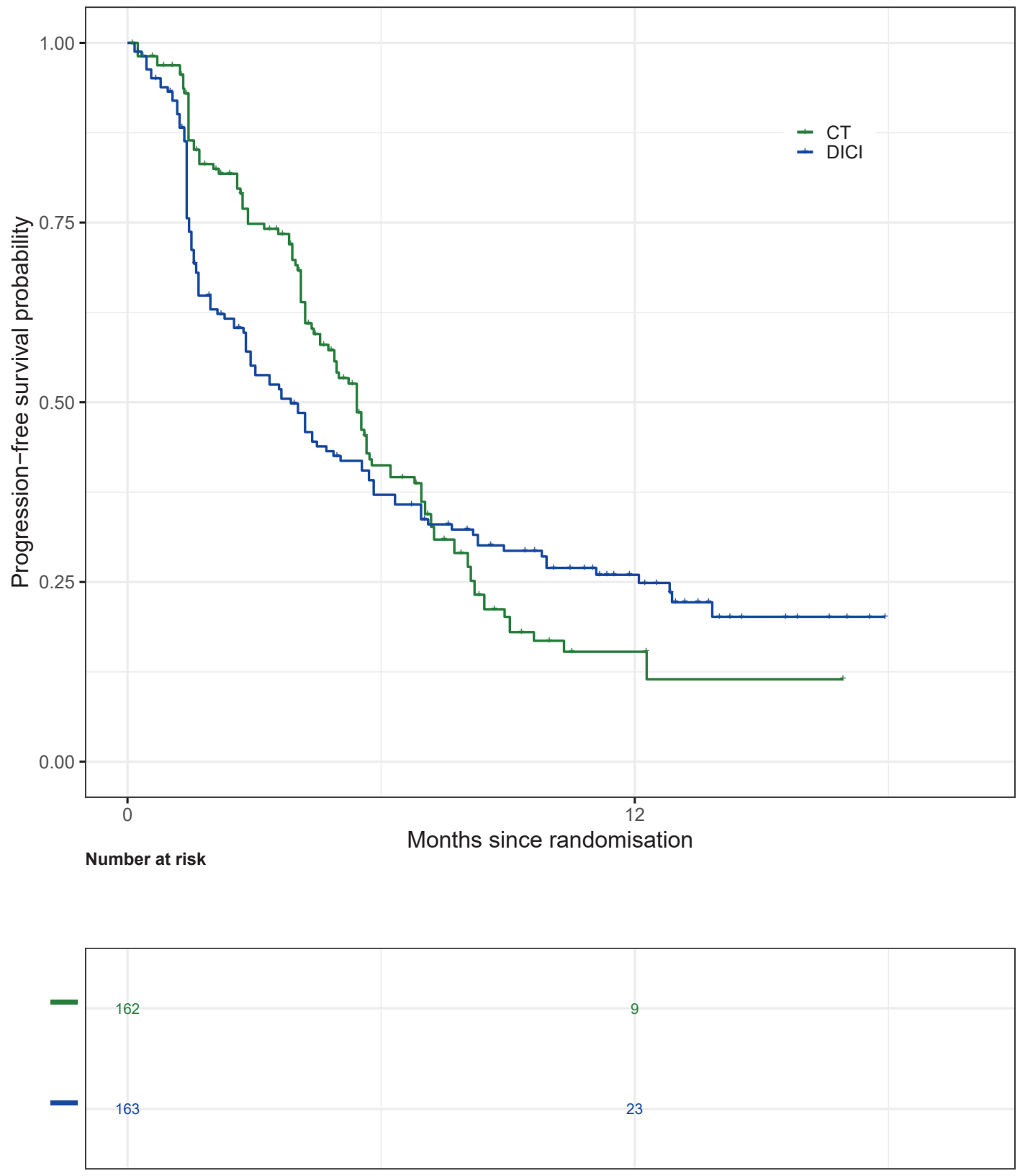

C) OS PD-L1 ≥ 25%

Treatment	N	Events	Median OS, mc (95% CI)	2−yr OS (95% CI)	3−yr OS (95% CI)	5−yr OS (95% CI)
CT	162	127	13.1 (10.8–15.2)	22.7% (17–30.3%)	NR	NR
DICI	163	113	12 (9.2–18.3)	35% (28.3–43.3%)	25.2% (17.1–37.2%)	NR

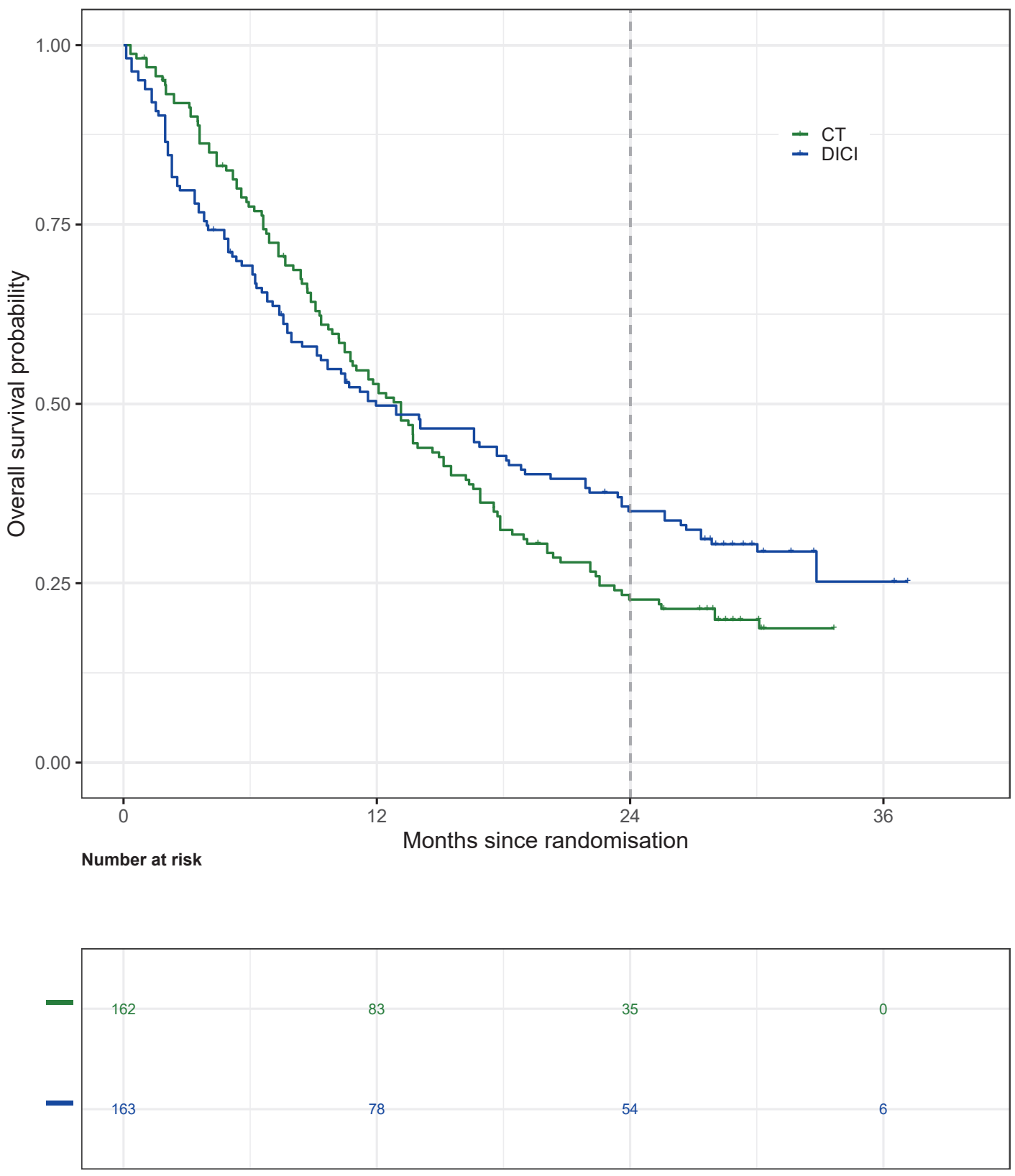

D) OS KEAP1 mutated

Treatment	N	Events	Median OS, mo (95% CI)	1−yr OS (95% CI)	2−yr OS (95% CI)
CT	53	49	6.2 (4.8–10.5)	28.3% (18.4–43.4%)	13.2% (6.6–26.3%)
DICI	63	52	9.2 (5–12.6)	36.5% (26.4–50.6%)	19% (11.4–31.7%)

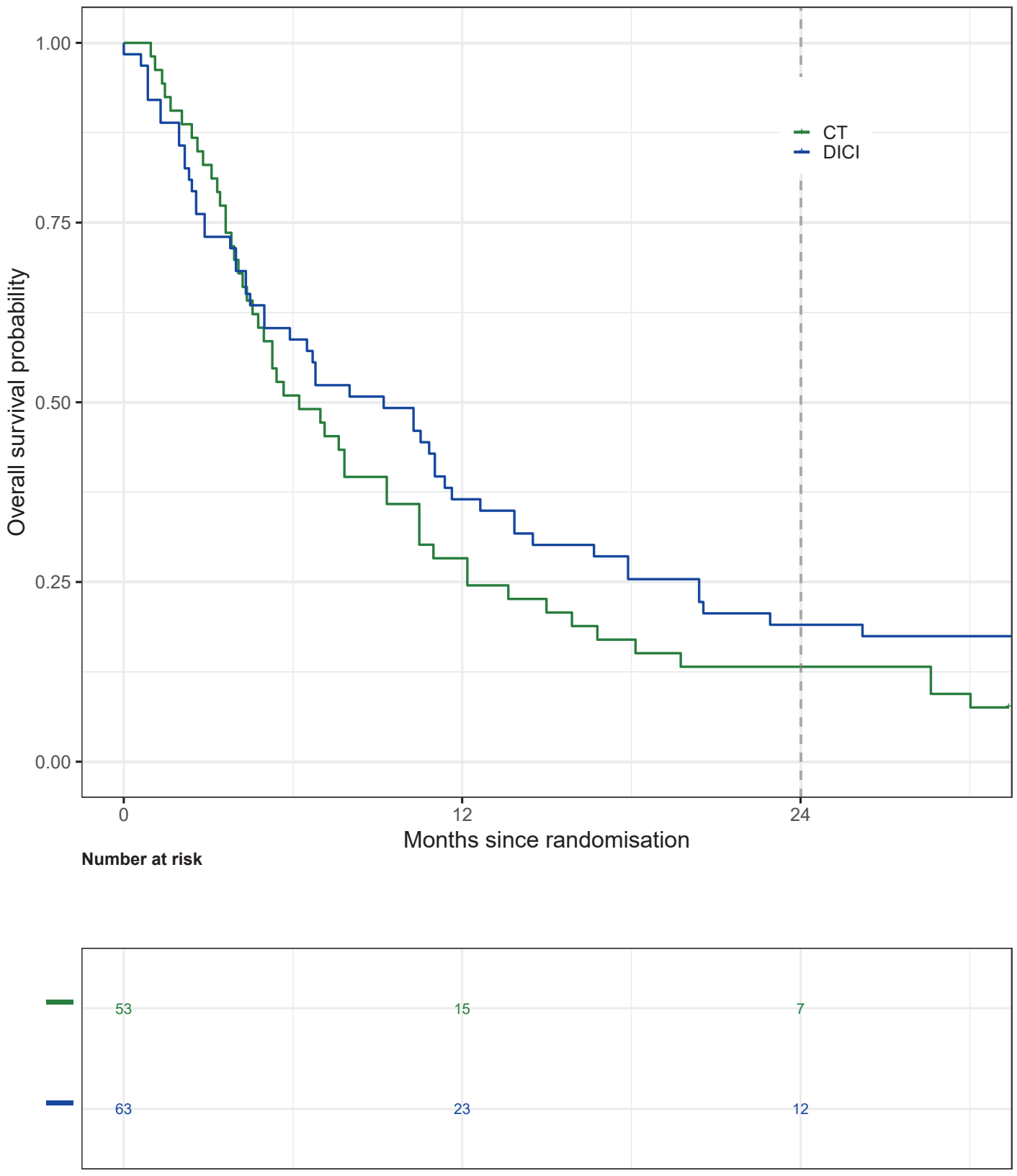

E) OS KEAP1 wild-type

Treatment	N	Events	Median OS, mo (95% CI)	1−yr OS (95% CI)	2−yr OS (95% CI)
CT	256	208	13.6 (12.1–15.9)	55.9% (50.1–62.3%)	29.3% (24.2–35.4%)
DICI	259	208	11.4 (9.5–14.3)	48.3% (42.5–54.7%)	29.7% (24.7–35.9%)

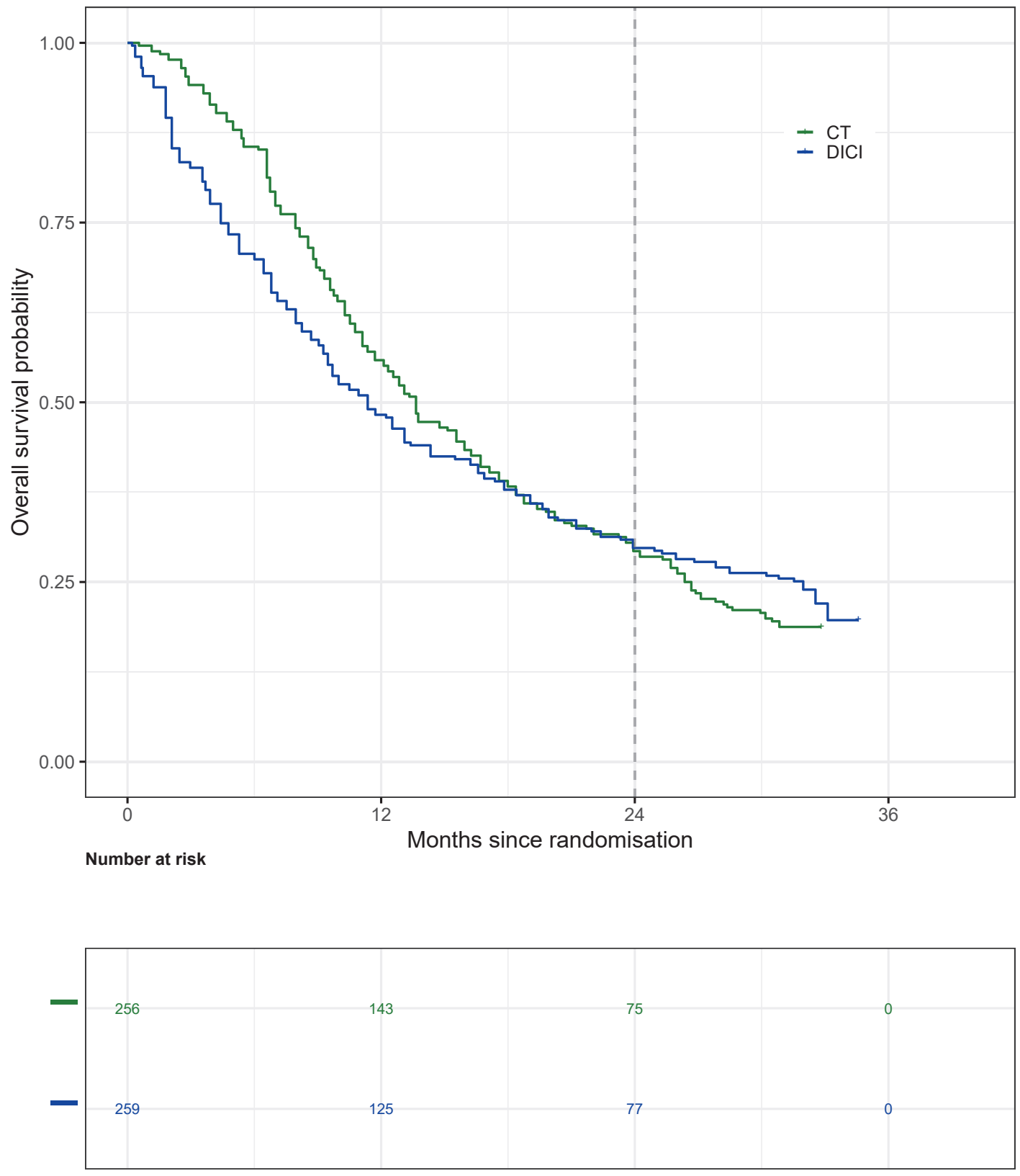

F) OS STK11 mutated

Treatment	N	Events	Median OS, mo (95% CI)	1−yr OS (95% CI)	2−yr OS (95% CI)
CT	41	40	6.5 (4.9–10.3)	22% (12.3–39.1%)	2.4% (0.4–16.9%)
DICI	51	42	4.4 (3.2–6.8)	27.5% (17.6–42.9%)	17.6% (9.8–31.9%)

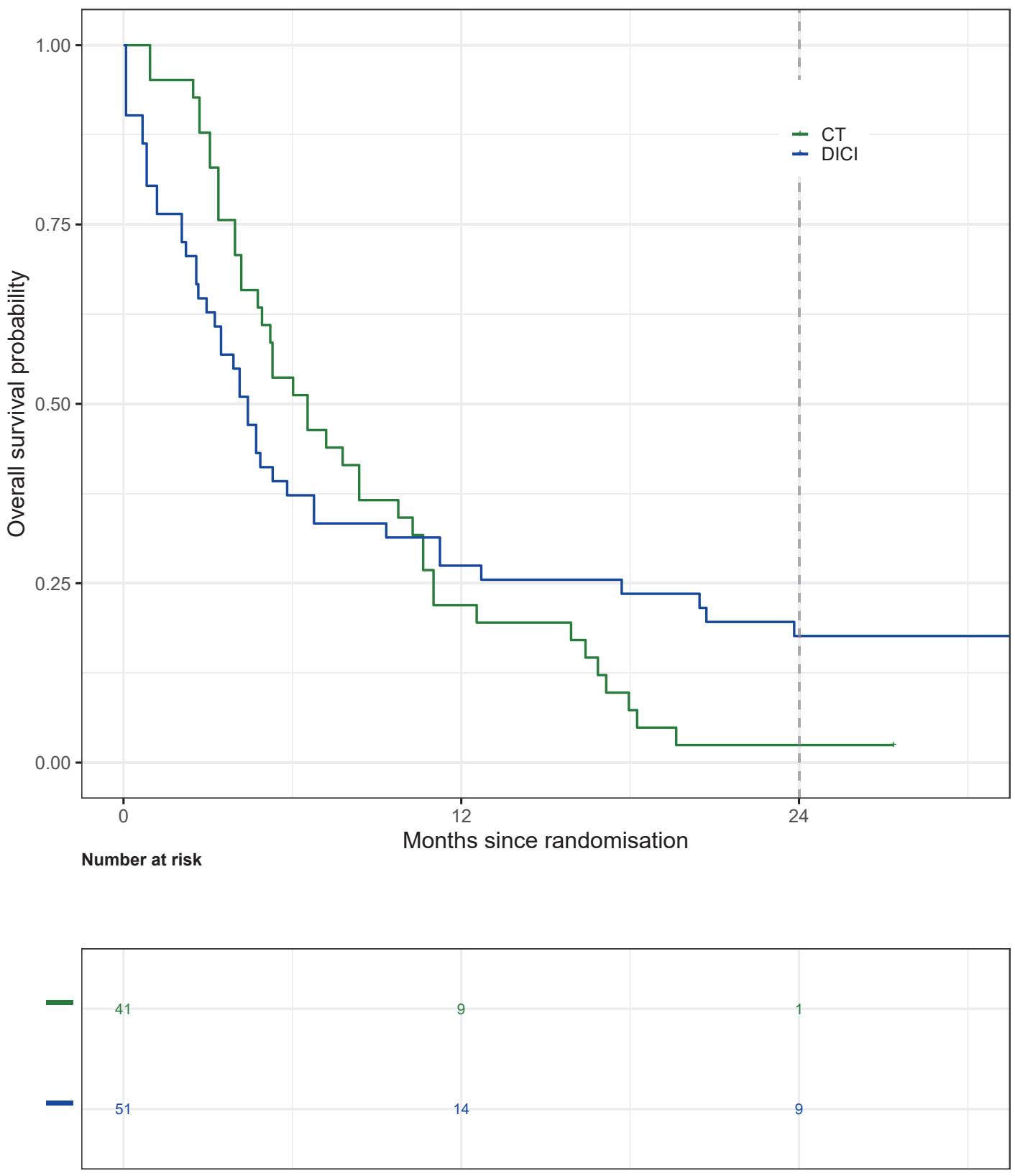

G) OS STK11 wild-type

Treatment	N	Events	Median OS, mo (95% CI)	1−yr OS (95% CI)	2−yr OS (95% CI)
CT	268	215	13.4 (11.9–15.6)	55.2% (49.6–61.5%)	29.9% (24.8–35.9%)
DICI	271	220	11.5 (10.5–13.8)	49.4% (43.8–55.8%)	28.4% (23.5–34.3%)

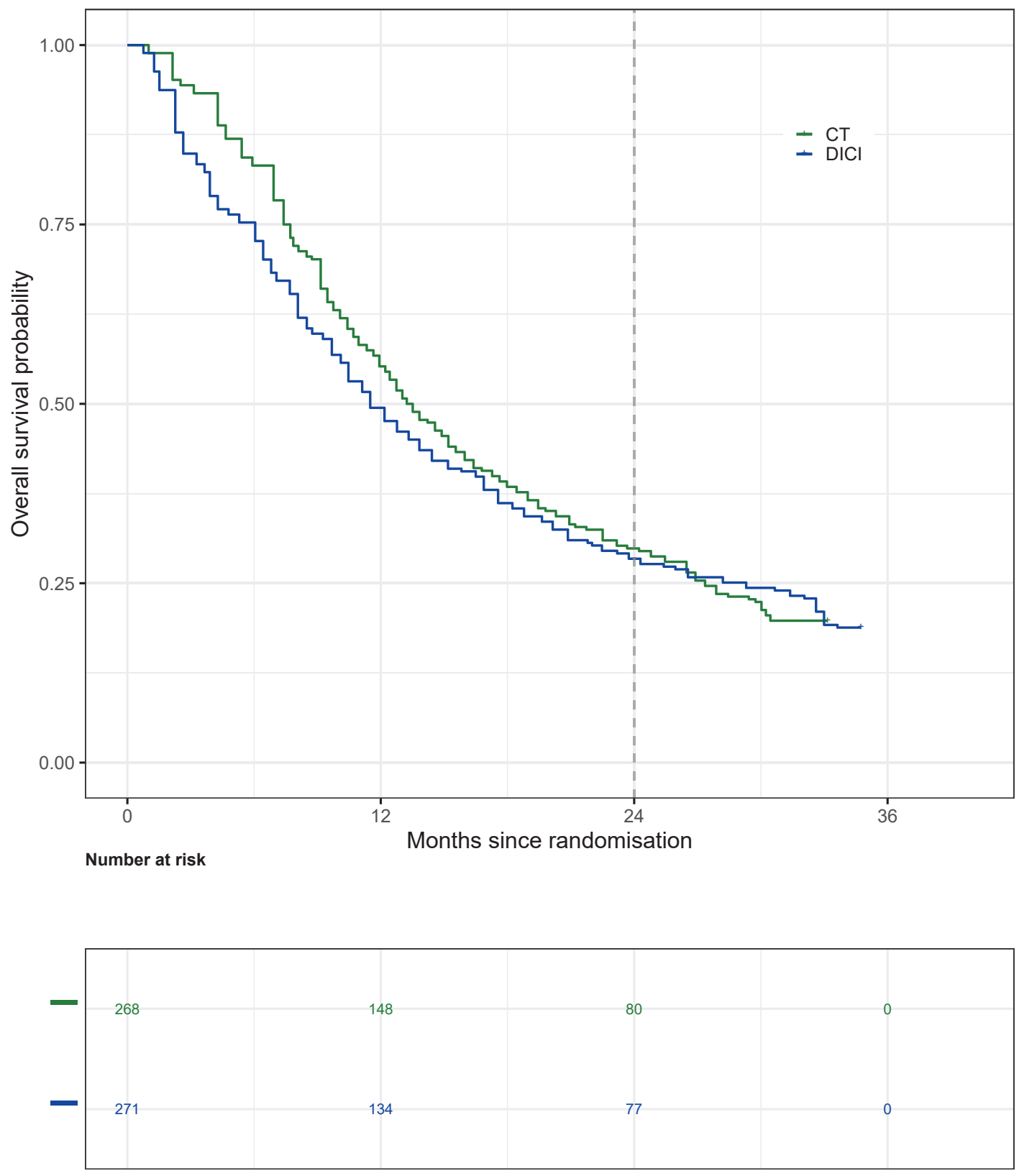

D) POSEIDON

A) OS all patients

Treatment	N	Events	Median OS, mc (95% CI)	2−yr OS (95% CI)	3−yr OS (95% CI)	5−yr OS (95% CI)
CT	337	297	11.8 (10.7–13.3)	22.5% (18.3–27.6%)	13.4% (10.1–17.8%)	7.3% (4.8–10.9%)
DICI−CT	338	274	14.1 (12–16.5)	33.5% (28.7–39%)	24.5% (20.2–29.6%)	15.5% (12–20.1%)

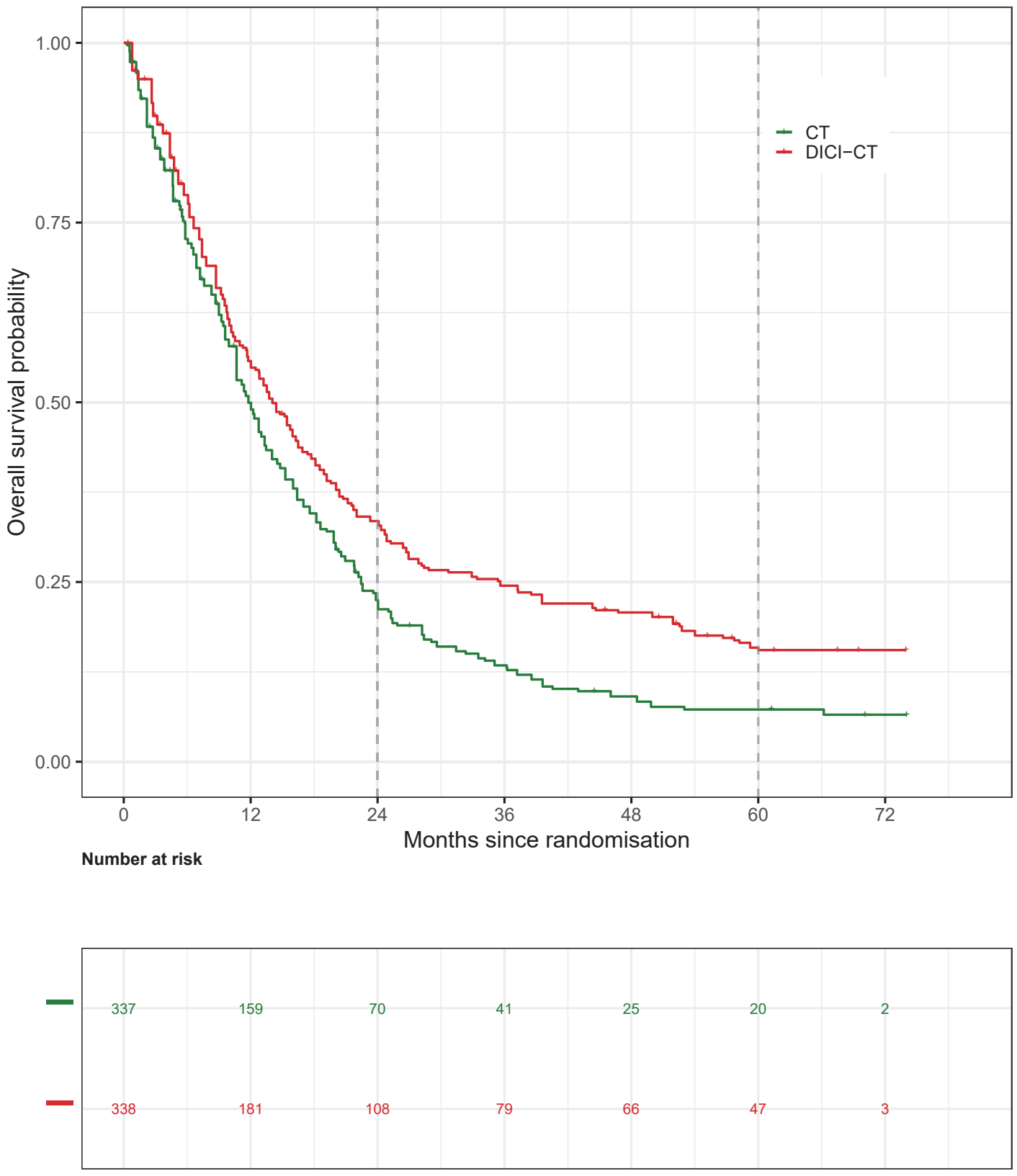

B) PFS all patients

Treatment	N	Events	Median PFS, mo (95% CI)	1−yr PFS (95% CI)	2−yr PFS (95% CI)
CT	337	257	4.8 (4.6–5.8)	13.9% (10.2–18.9%)	NR
DICI−CT	338	237	6.3 (5–6.5)	27.2% (22.5–32.7%)	NR

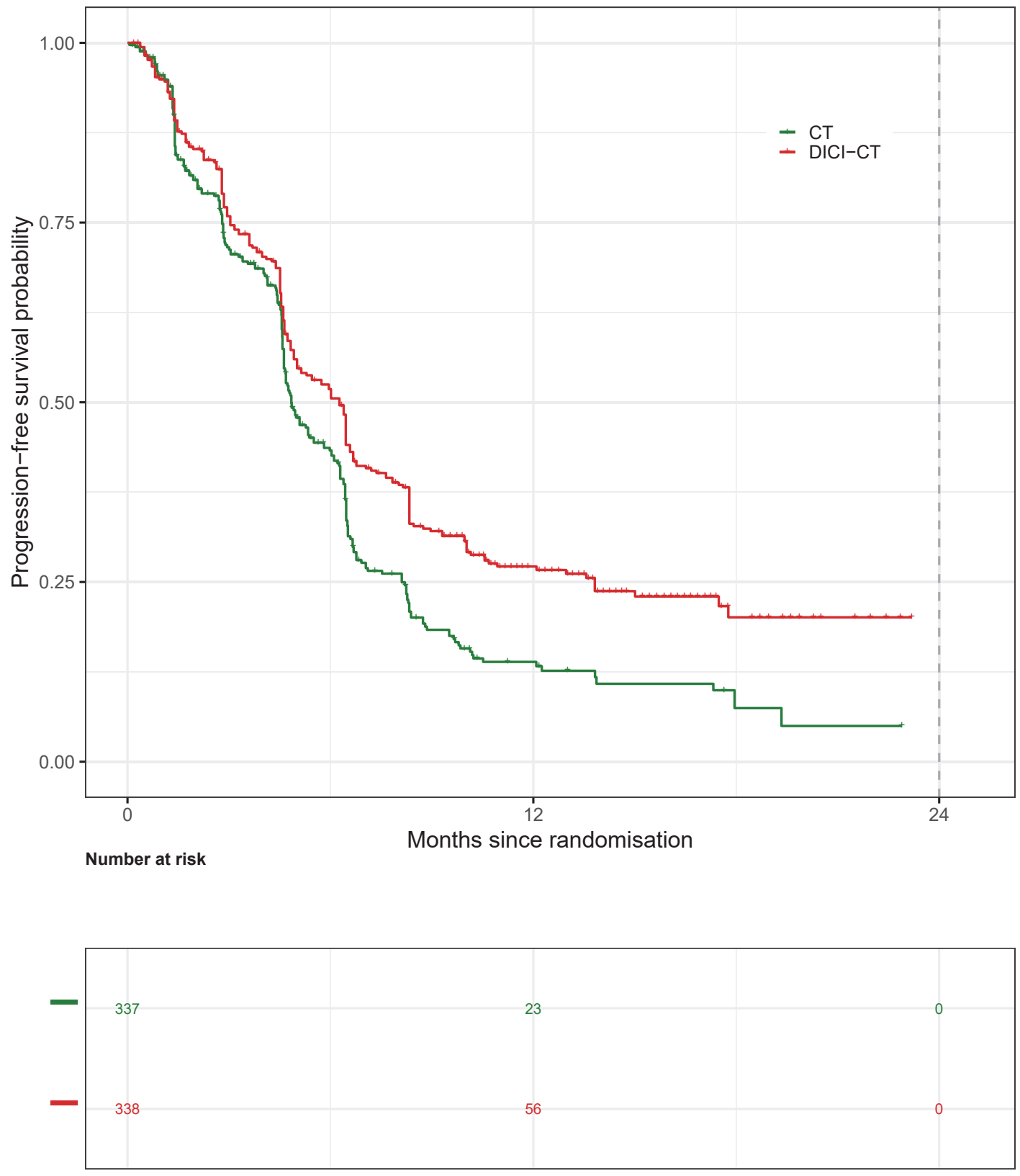

C) OS PD-L1 ≥ 1%

Treatment	N	Events	Median OS, mc (95% CI)	2−yr OS (95% CI)	3−yr OS (95% CI)	5−yr OS (95% CI)
CT	207	181	12.4 (10.4–15.1)	24.6% (19.2–31.3%)	16.8% (12.3–22.9%)	9.1% (5.8–14.4%)
DICI−CT	213	161	15.8 (11.5–19.6)	38.9% (32.8–46.1%)	28.7% (23.1–35.5%)	21.6% (16.2–28.8%)

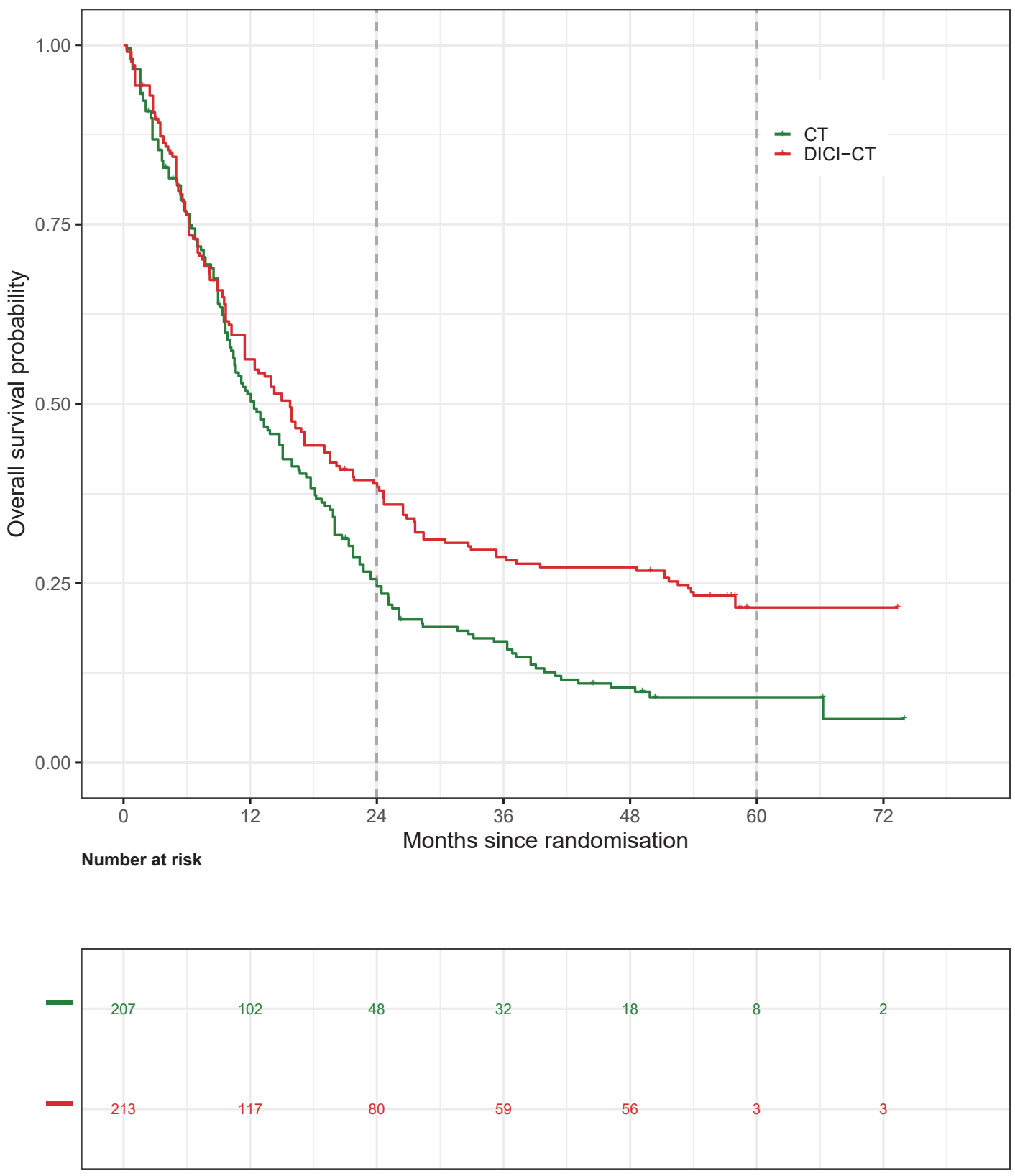

D) OS PD-L1 < 1%

Treatment	N	Events	Median OS, mc (95% CI)	2−yr OS (95% CI)	3−yr OS (95% CI)	5−yr OS (95% CI)
CT	130	118	10.8 (8.9–13.3)	18.5% (12.8–26.9%)	8.4% (4.7–15.2%)	4.2% (1.8–9.9%)
DICI−CT	125	103	12.9 (10.7–16.6)	24.4% (17.4–34.1%)	18.5% (12.4–27.7%)	6.4% (3–13.6%)

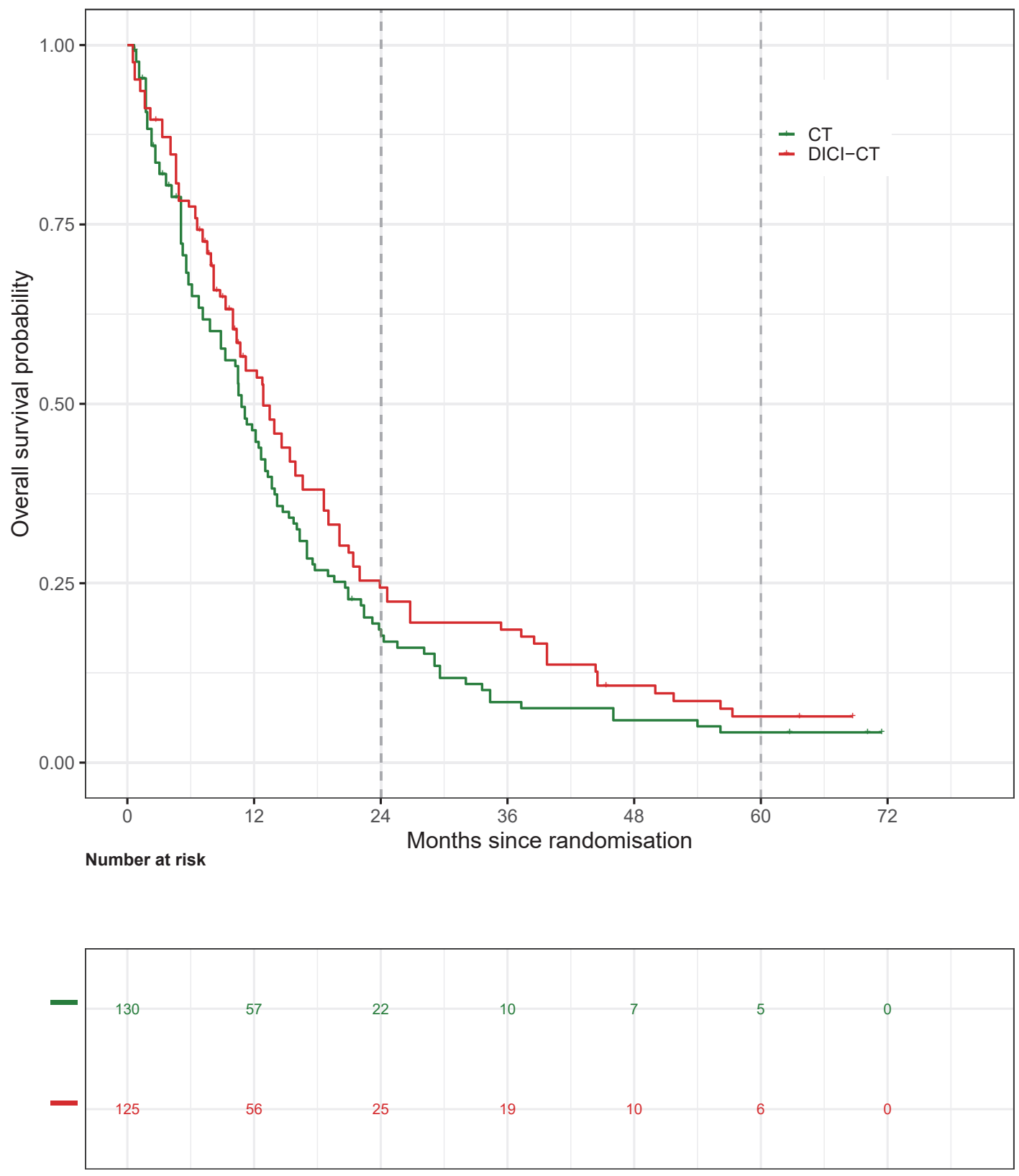

E) OS SQUAMOUS

Treatment	N	Events	Median OS, mc (95% CI)	2−yr OS (95% CI)	3−yr OS (95% CI)	5−yr OS (95% CI)
CT	122	114	10.7 (8.9–12)	14.1% (9–22.1%)	7.9% (4.3–14.8%)	2.7% (0.8–9.2%)
DICI−CT	124	112	10.6 (9.1–13.2)	18.6% (12.8–27.1%)	13.5% (8.6–21.3%)	7.1% (3.6–13.8%)

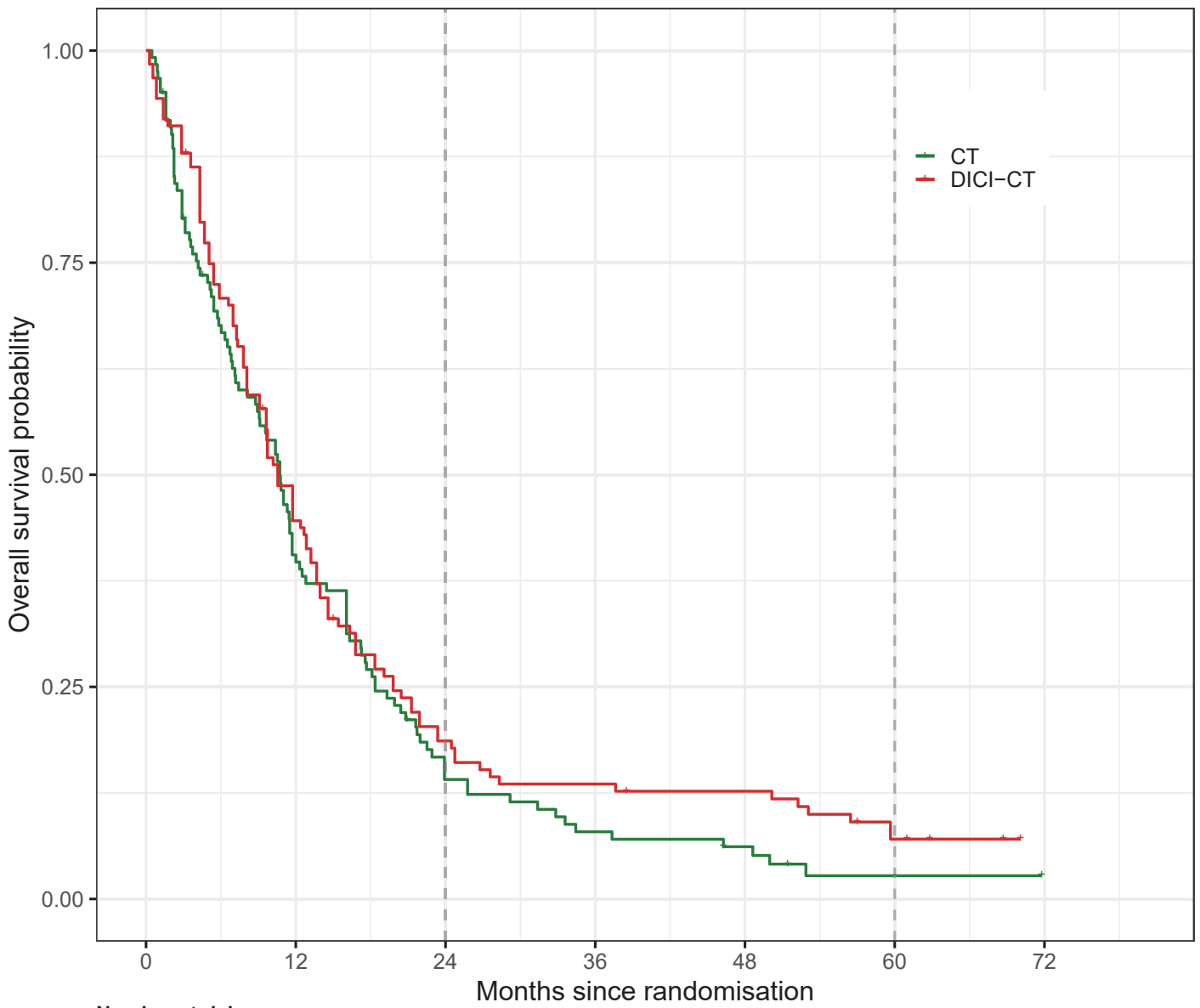

Number at risk

	0	12	24	36	48	60	72
CT	122	48	16	9	6	2	0
DICI−CT	124	54	22	16	14	7	0

F) OS NONSQUAMOUS

Treatment	N	Events	Median OS, mc (95% CI)	2−yr OS (95% CI)	3−yr OS (95% CI)	5−yr OS (95% CI)
CT	214	179	13.1 (11.3–15.6)	27% (21.5–34%)	16.7% (12.2–22.8%)	9.3% (6–14.5%)
DICI−CT	214	165	17.2 (15.3–22)	41.8% (35.6–49%)	31.9% (26.2–39%)	20.9% (16–27.3%)

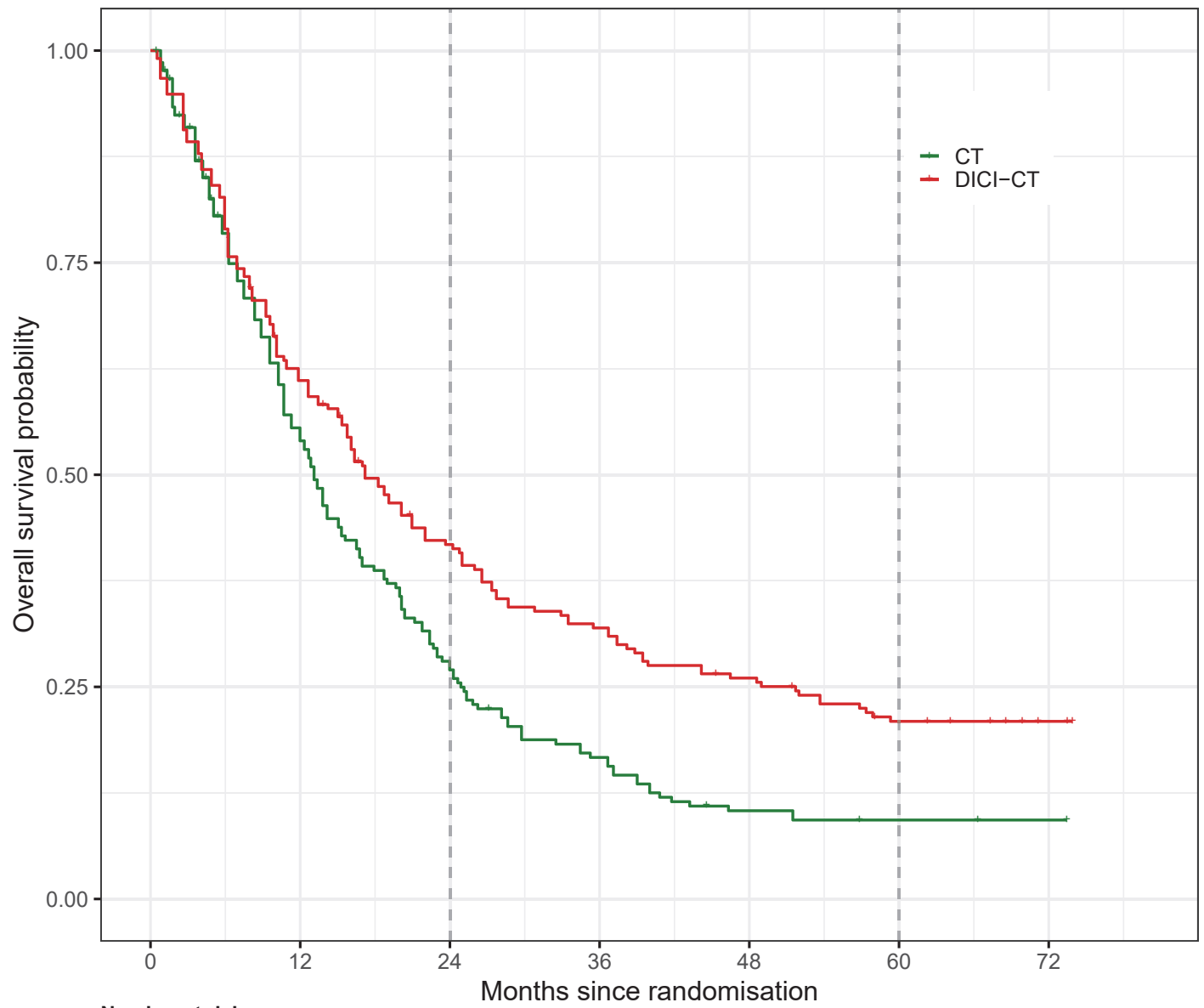

Number at risk

	0	12	24	36	48	60	72
CT	214	106	53	32	19	8	2
DICI−CT	214	129	85	65	52	40	3

G) OS KEAP1 mutated

Treatment	N	Events	Median OS, mo (95% CI)	2−yr OS (95% CI)	5−yr OS (95% CI)
CT	6	5	8.7 (6–NA)	NR	NR
DICI−CT	22	19	13.5 (9.3–37.4)	35% (19.5–62.7%)	10% (2.7–37%)

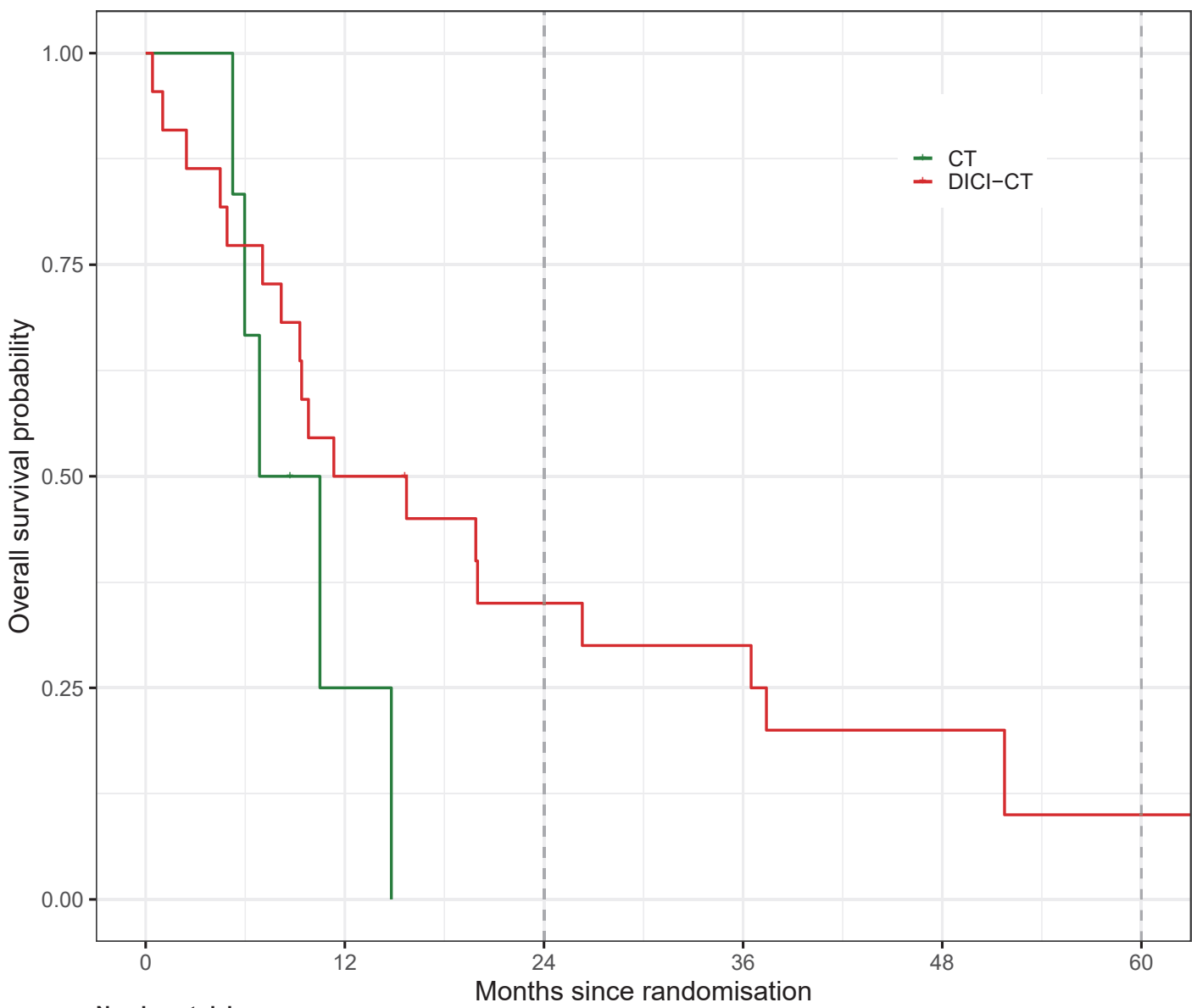

Number at risk

	0	12	24	36	48	60
CT	6	1	0	0	0	0
DICI−CT	22	11	7	6	4	2

H) OS KEAP1 wild-type

Treatment	N	Events	Median OS, mc (95% CI)	2−yr OS (95% CI)	3−yr OS (95% CI)	5−yr OS (95% CI)
CT	312	276	12 (10.7–14.1)	24.3% (19.9–29.8%)	13.6% (10.2–18.2%)	6.4% (4–10.2%)
DICI−CT	303	240	14.1 (13–17.1)	33.5% (28.5–39.5%)	24.5% (20–30.1%)	16.5% (12.7–21.5%)

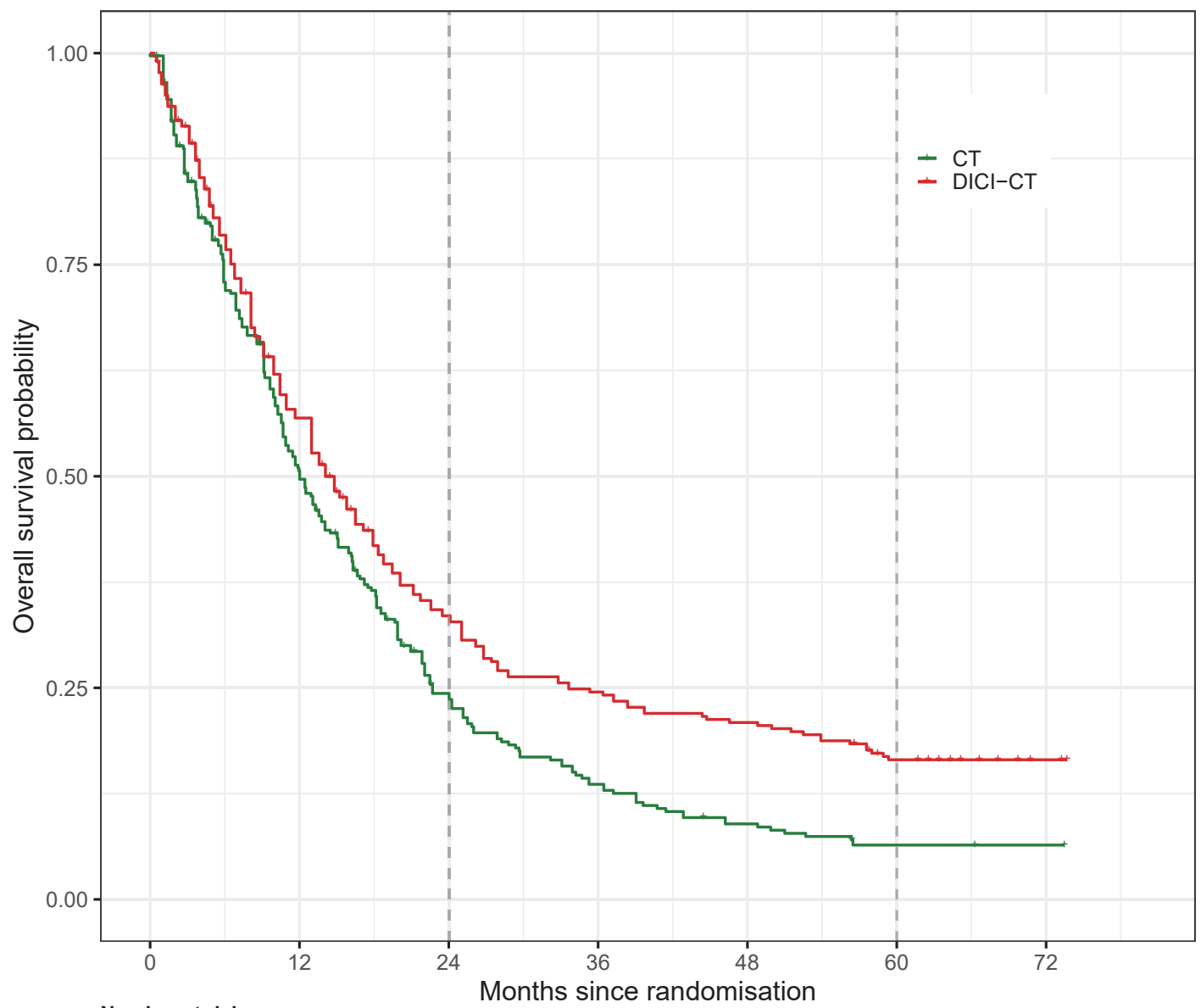

Number at risk

	0	12	24	36	48	60	72
CT	312	152	68	38	24	10	2
DICI−CT	303	165	93	68	58	43	3

I) OS STK11 mutated

Treatment	N	Events	Median OS, mo (95% CI)	2−yr OS (95% CI)	5−yr OS (95% CI)
CT	22	22	10.5 (6.8–14.9)	4.5% (0.7–30.8%)	NR
DICI−CT	31	27	14.9 (9.4–35.2)	32.3% (19.4–53.7%)	12.9% (5.2–32.2%)

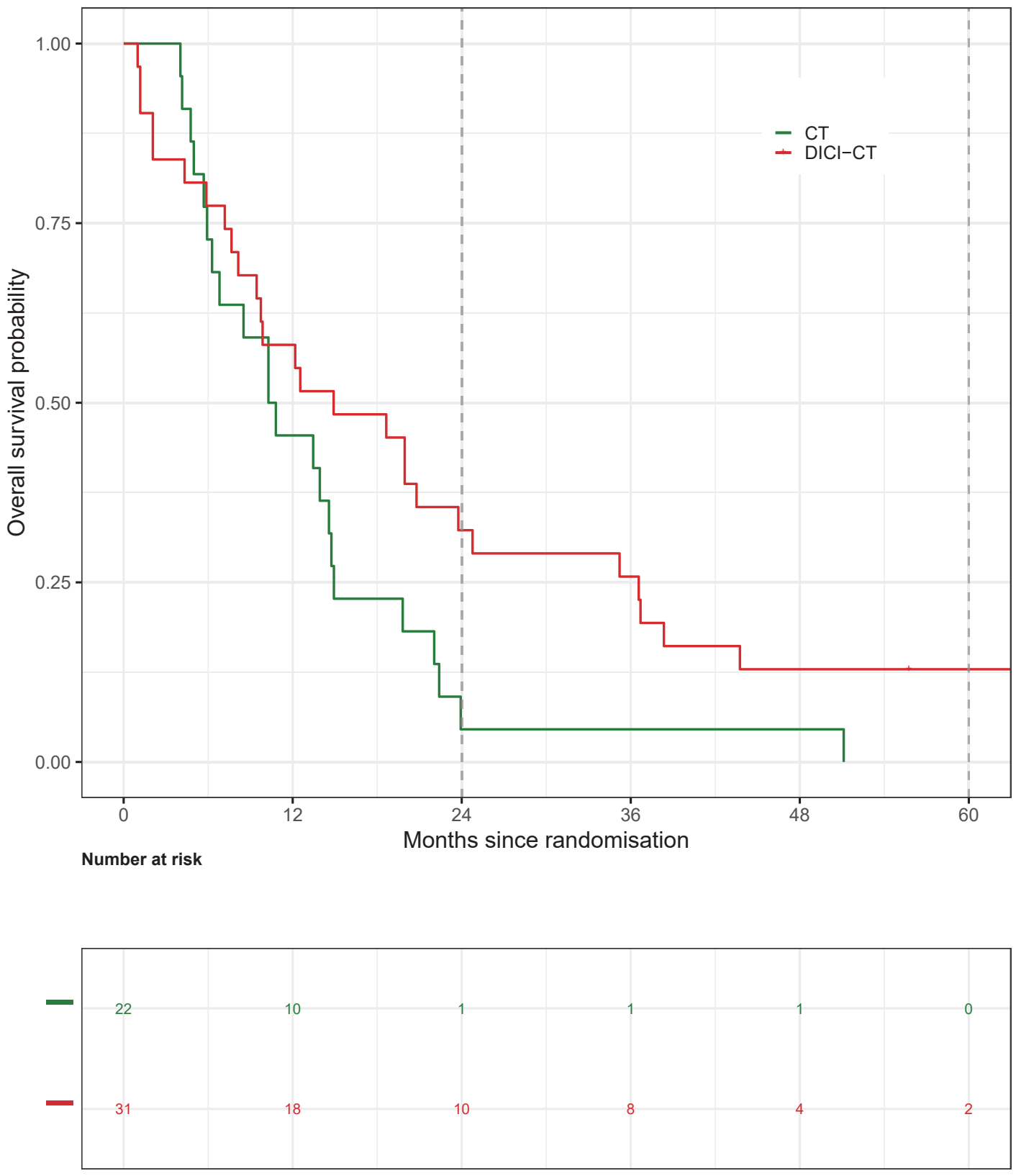

J) OS STK11 wild-type

Treatment	N	Events	Median OS, mo (95% CI)	2−yr OS (95% CI)	5−yr OS (95% CI)
CT	179	152	13.3 (12–17.9)	30.1% (23.9–37.9%)	10.5% (6.7–16.4%)
DICI−CT	177	134	17.4 (15.3–24.8)	43.2% (36.4–51.2%)	22.1% (16.6–29.4%)

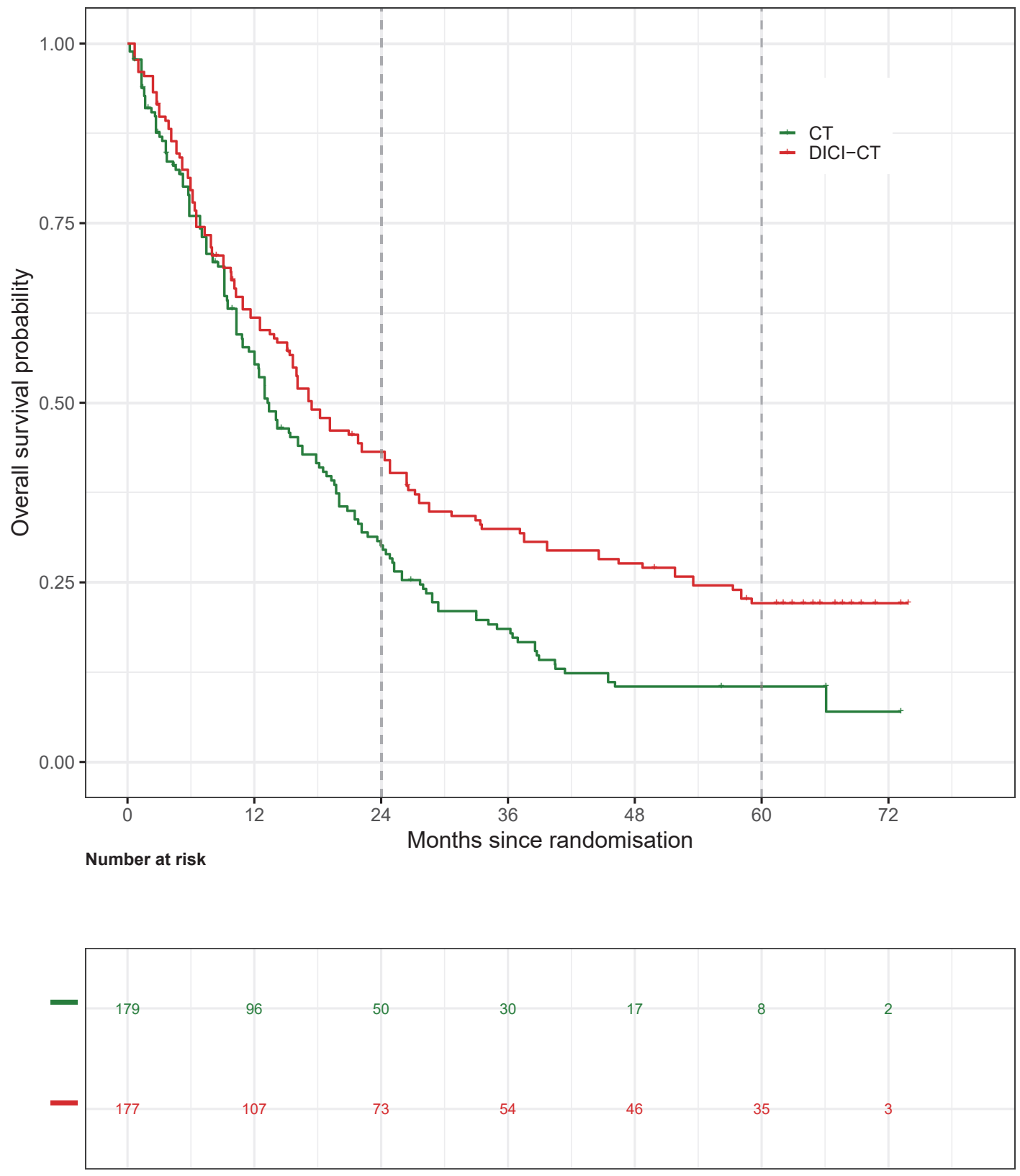

Supplementary Figure 2. Schoenfeld residuals plots: OS and PFS (all patients; PD-L1 ≥ 1%; PD-L1 < 1%; Squamous and Nonqsquamous patients)

A) OS all patients

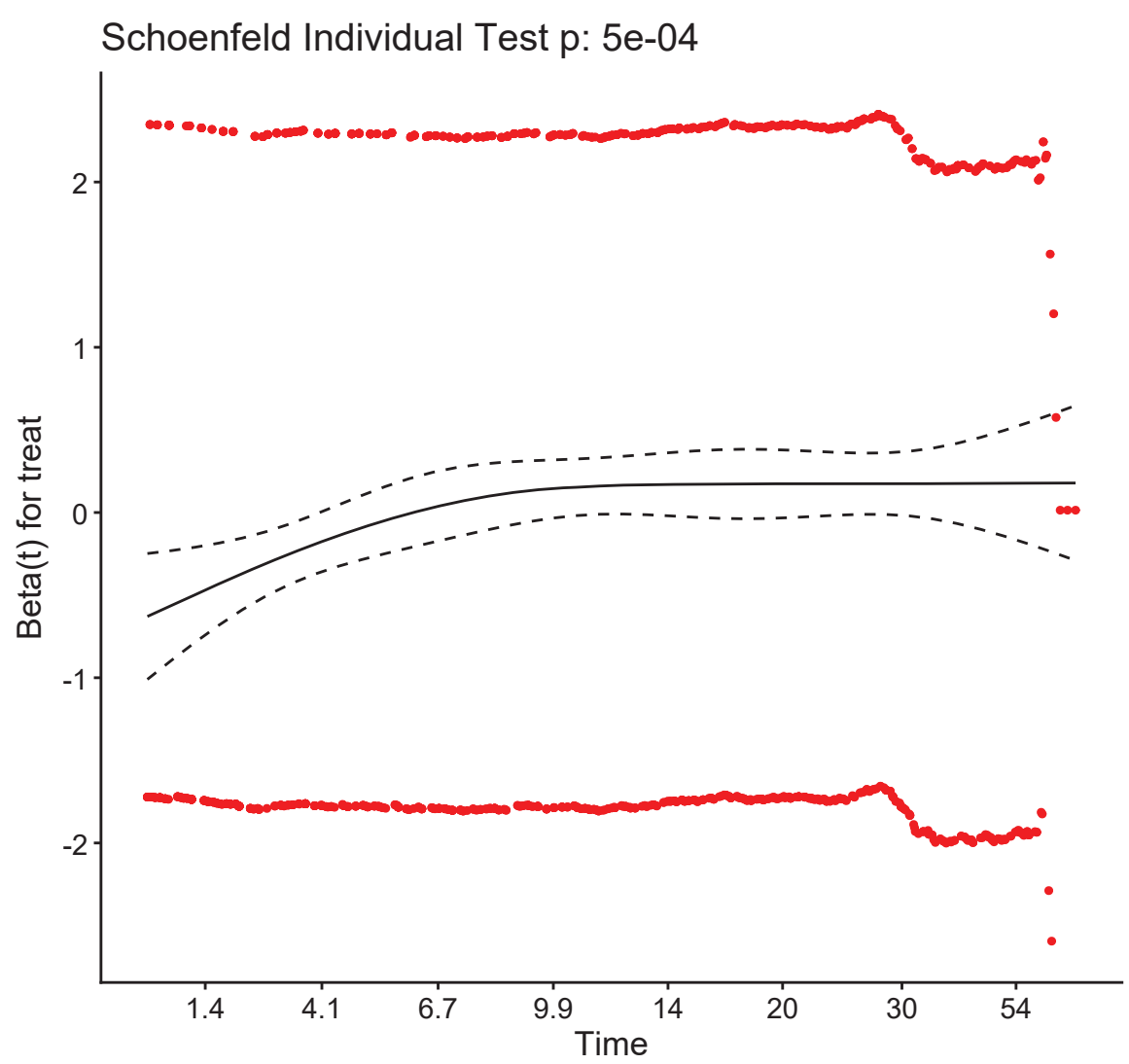

B) PFS all patients

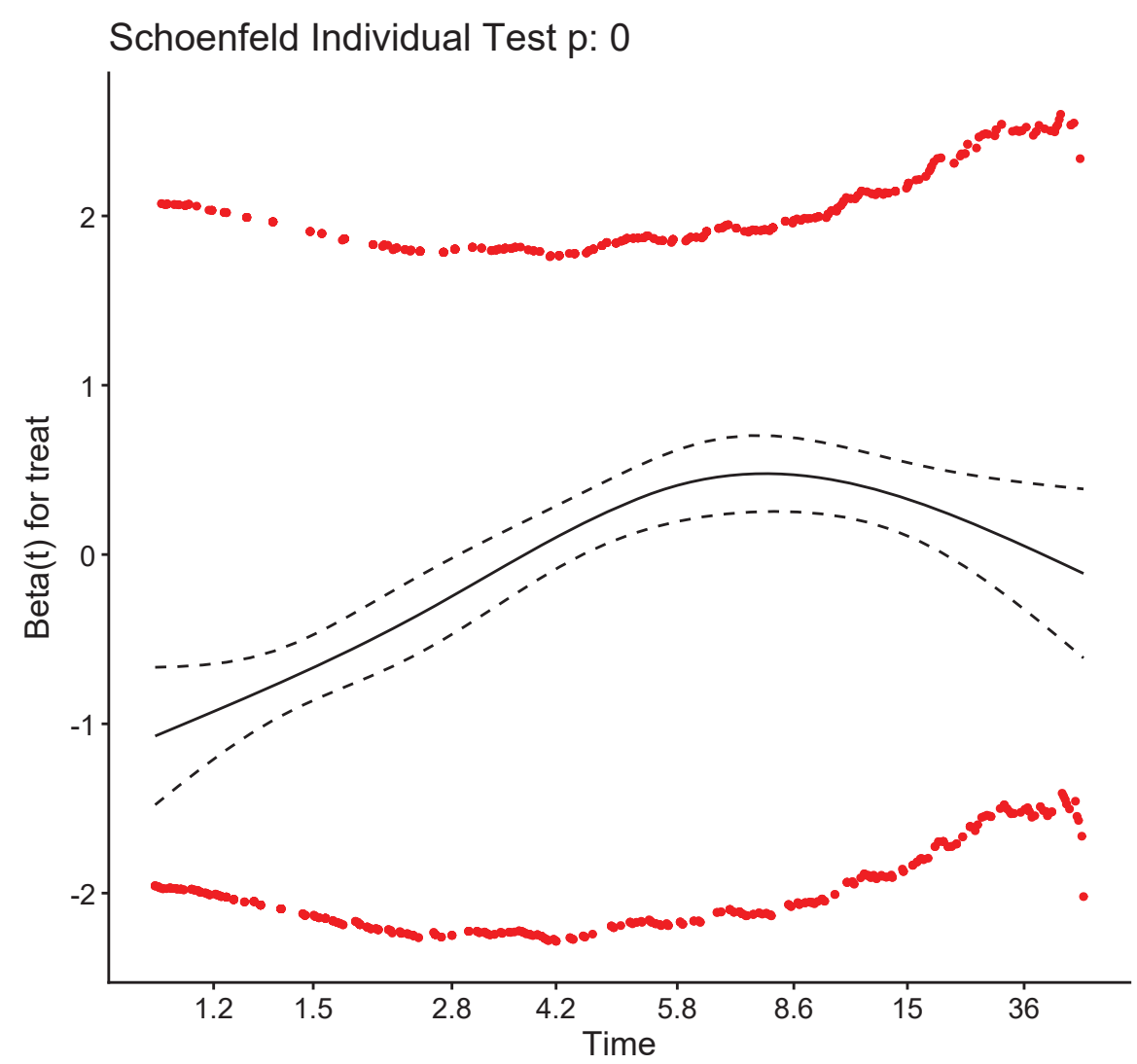

C) OS PD-L1 ≥ 1%

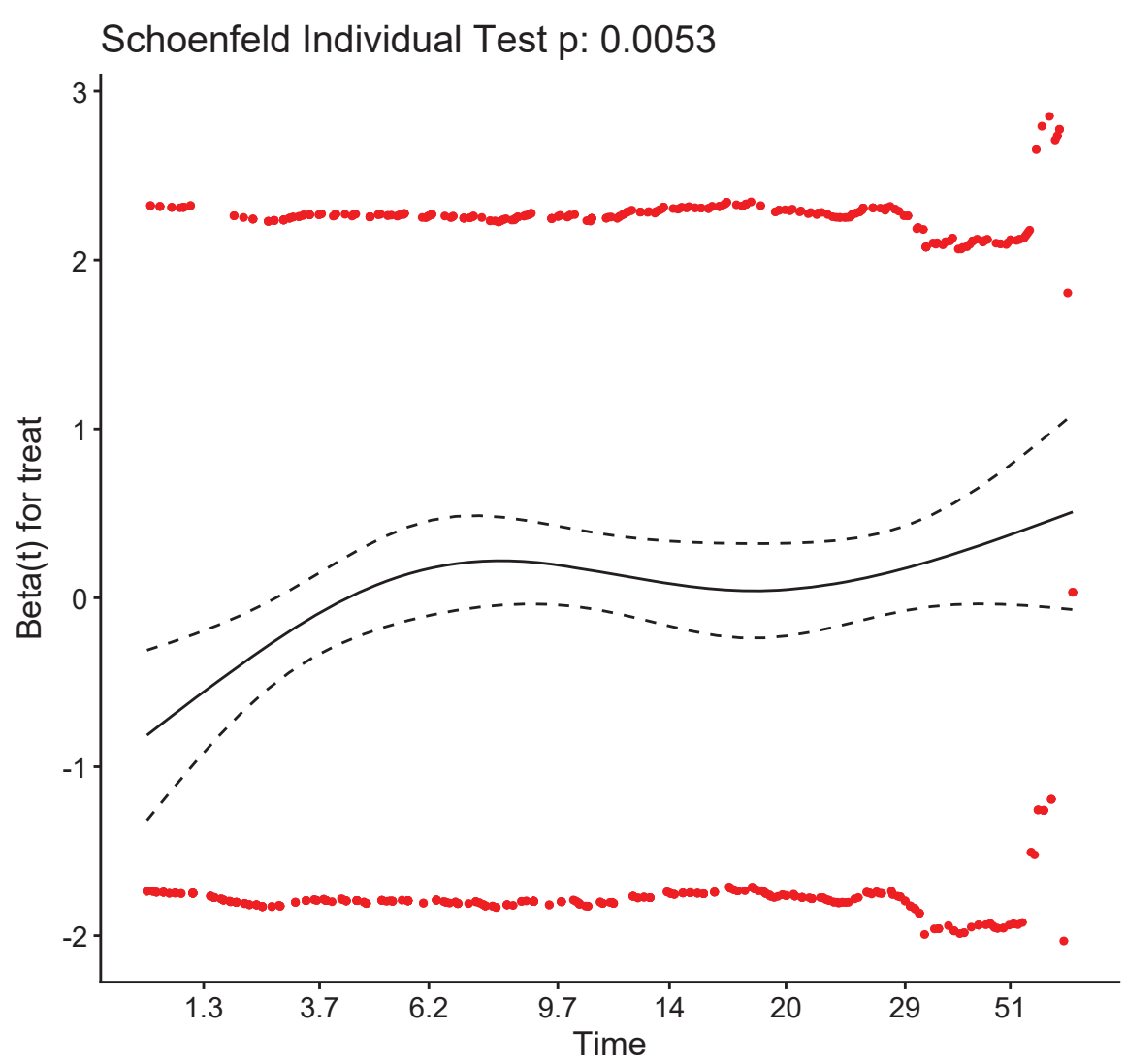

D) OS PD-L1 < 1%

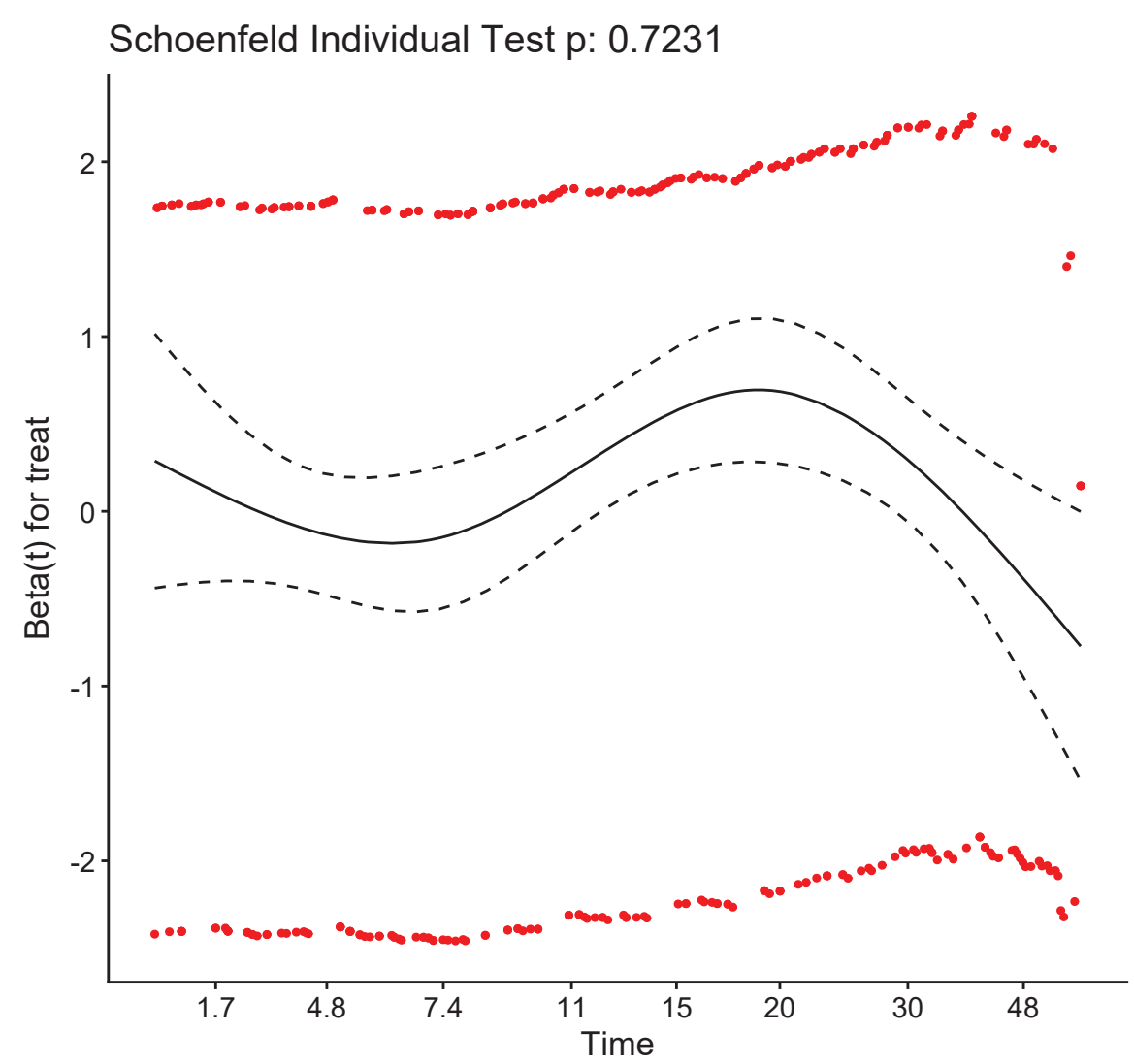

E) OS Squamous

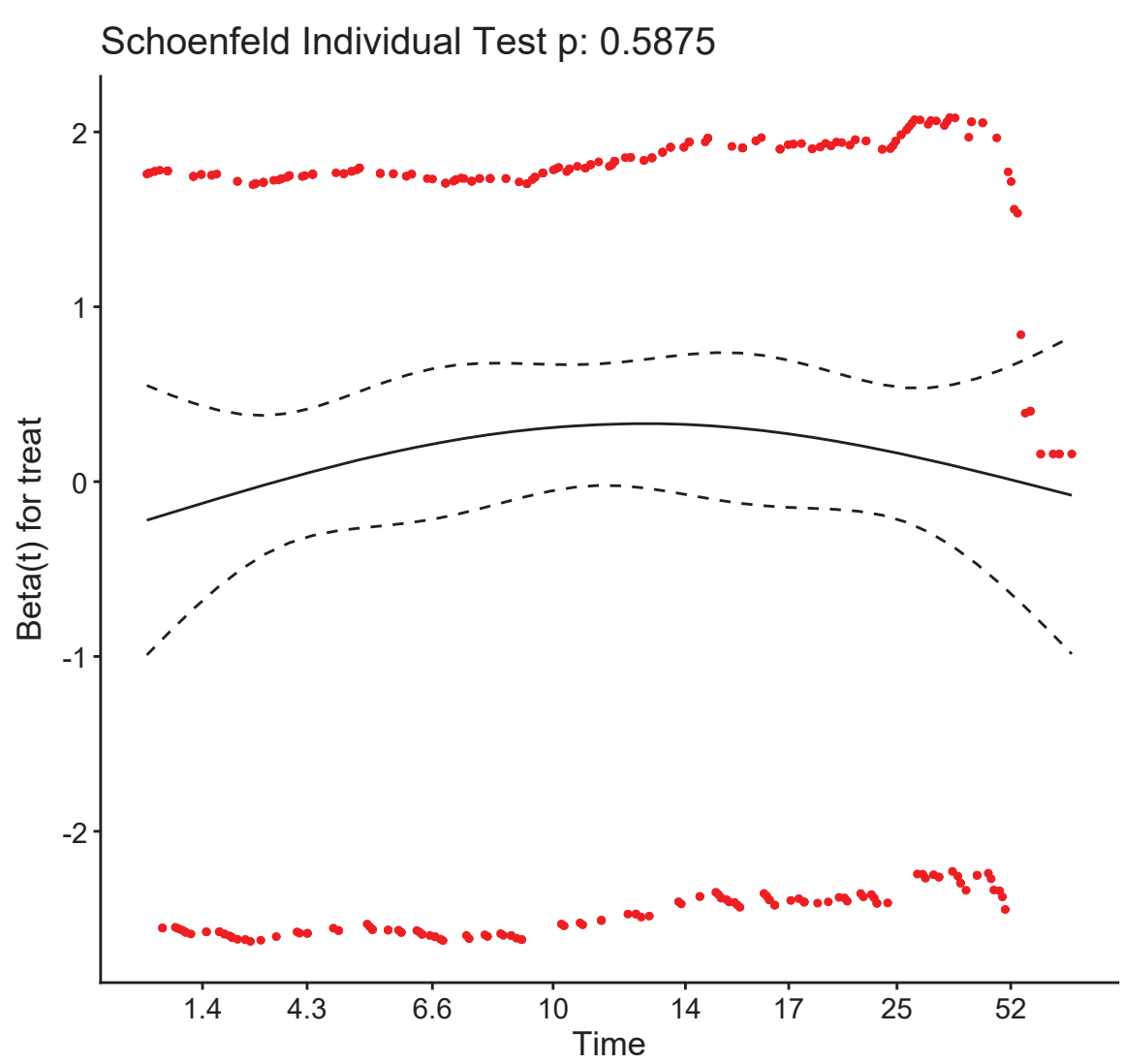

F) OS Nonsquamous

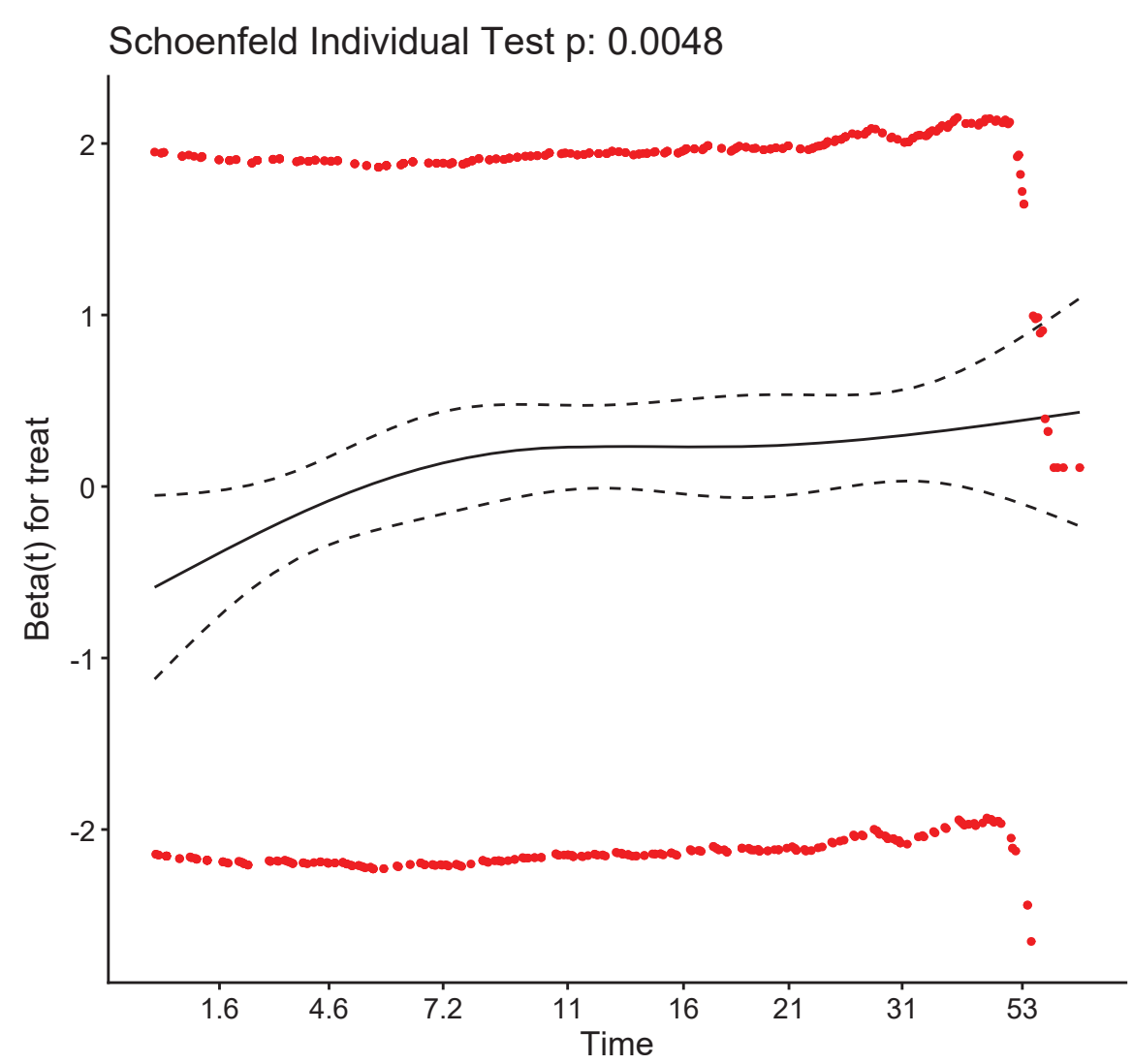

Supplementary Figure 3. Time-varying hazard ratios

A) OS all patients

Time (mo)	HR(t) (95% CI)	P−value
0	0.59 (0.43–0.80)*	<0.001*
12	1.05 (0.94–1.17)	0.406
24	1.21 (1.05–1.41)*	0.009*
36	1.33 (1.11–1.59)*	0.002*
48	1.41 (1.15–1.74)*	0.001*
60	1.49 (1.18–1.87)*	<0.001*

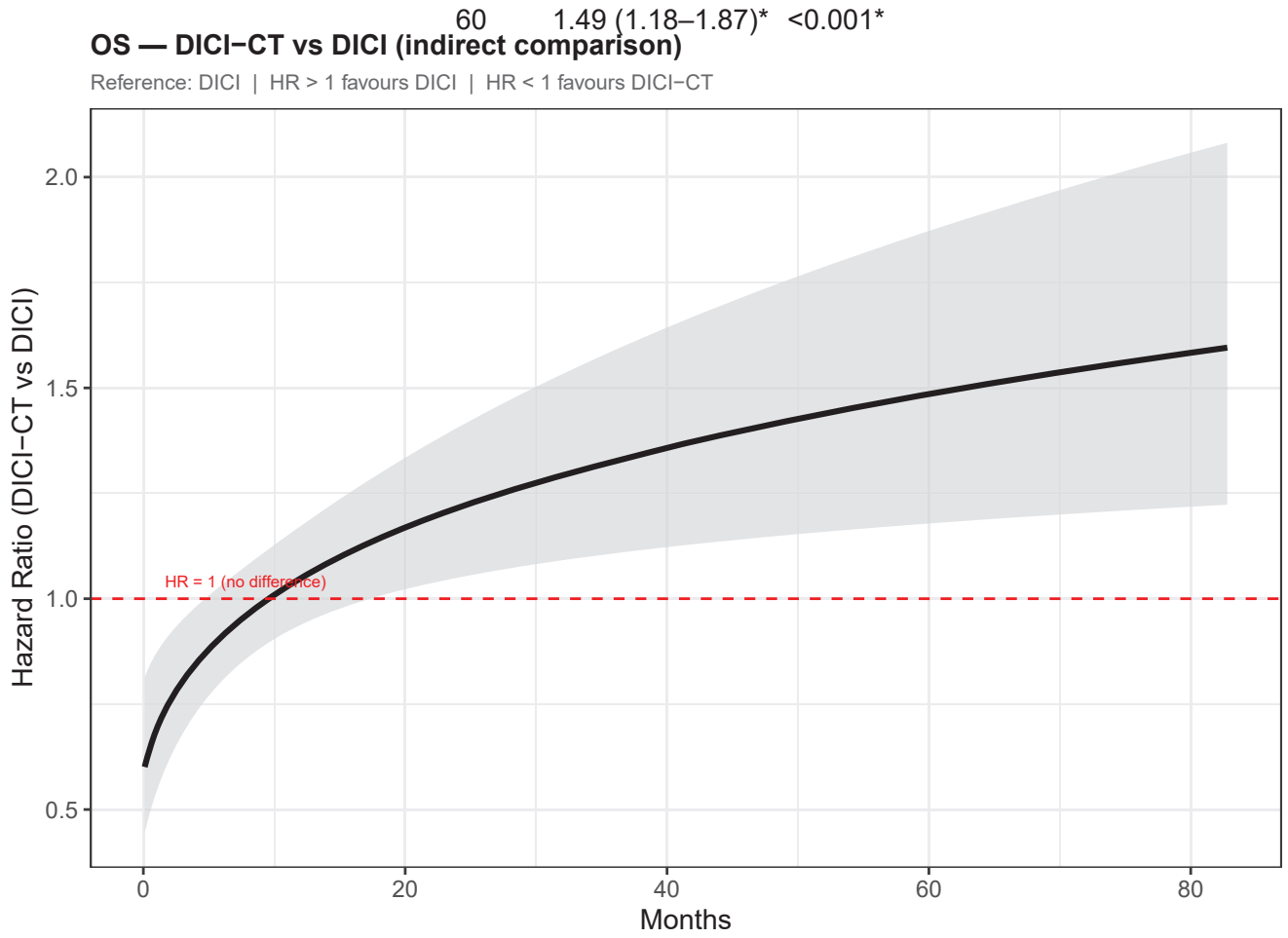

B) PFS all patients

Time (mo)	HR(t) (95% CI)	P−value
0	0.41 (0.31–0.54)*	<0.001*
12	1.31 (1.11–1.55)*	0.001*
24	1.76 (1.38–2.24)*	<0.001*
36	2.10 (1.57–2.81)*	<0.001*
48	2.39 (1.72–3.32)*	<0.001*
60	2.64 (1.84–3.77)*	<0.001*

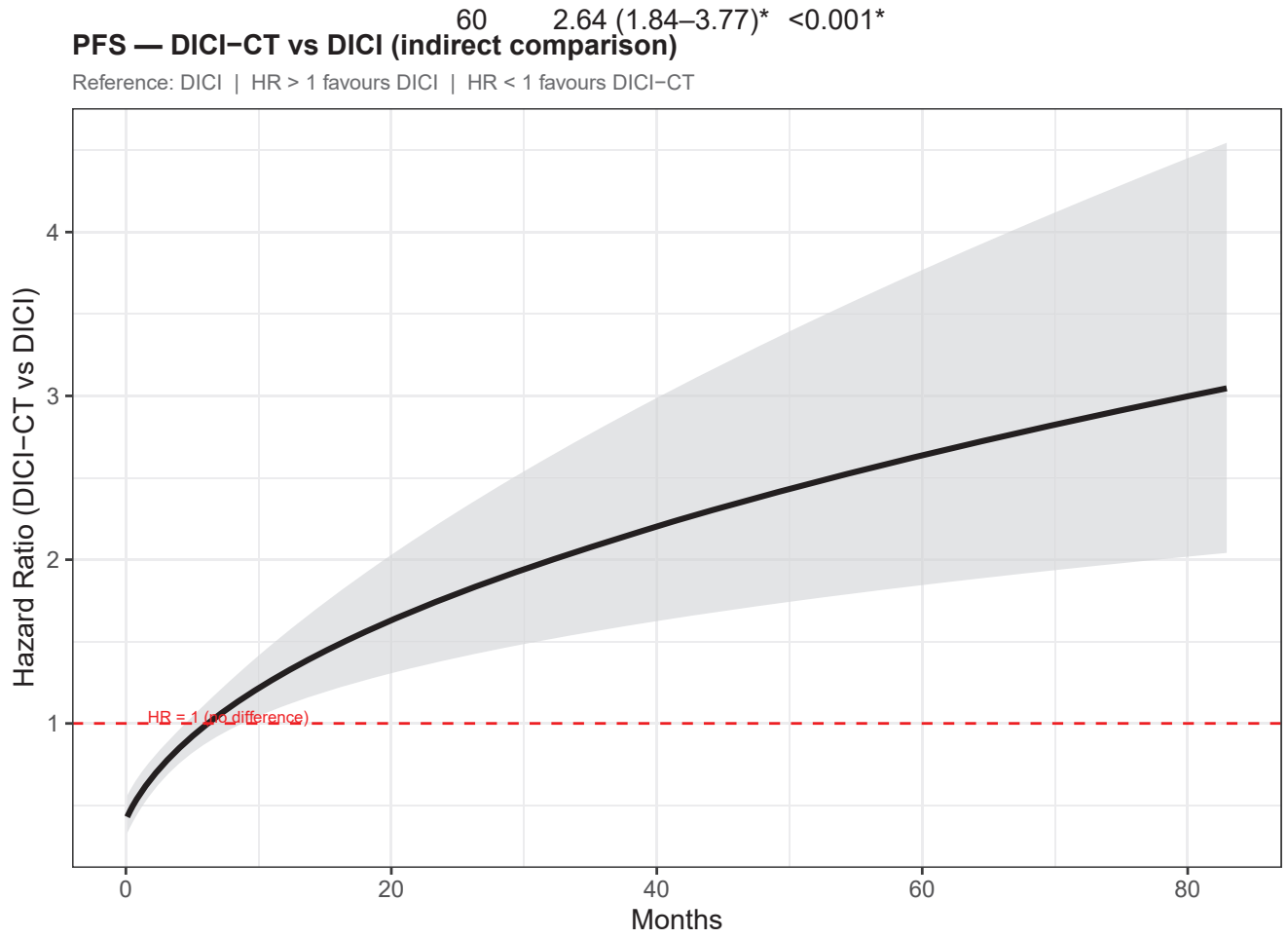

C) OS PD-L1 ≥ 1%

Time (mo)	HR(t) (95% CI)	P−value
0	0.57 (0.38–0.86)*	0.007*
12	1.07 (0.93–1.25)	0.339
24	1.26 (1.04–1.53)*	0.018*
36	1.39 (1.10–1.76)*	0.007*
48	1.49 (1.13–1.96)*	0.004*
60	1.57 (1.16–2.13)*	0.003*

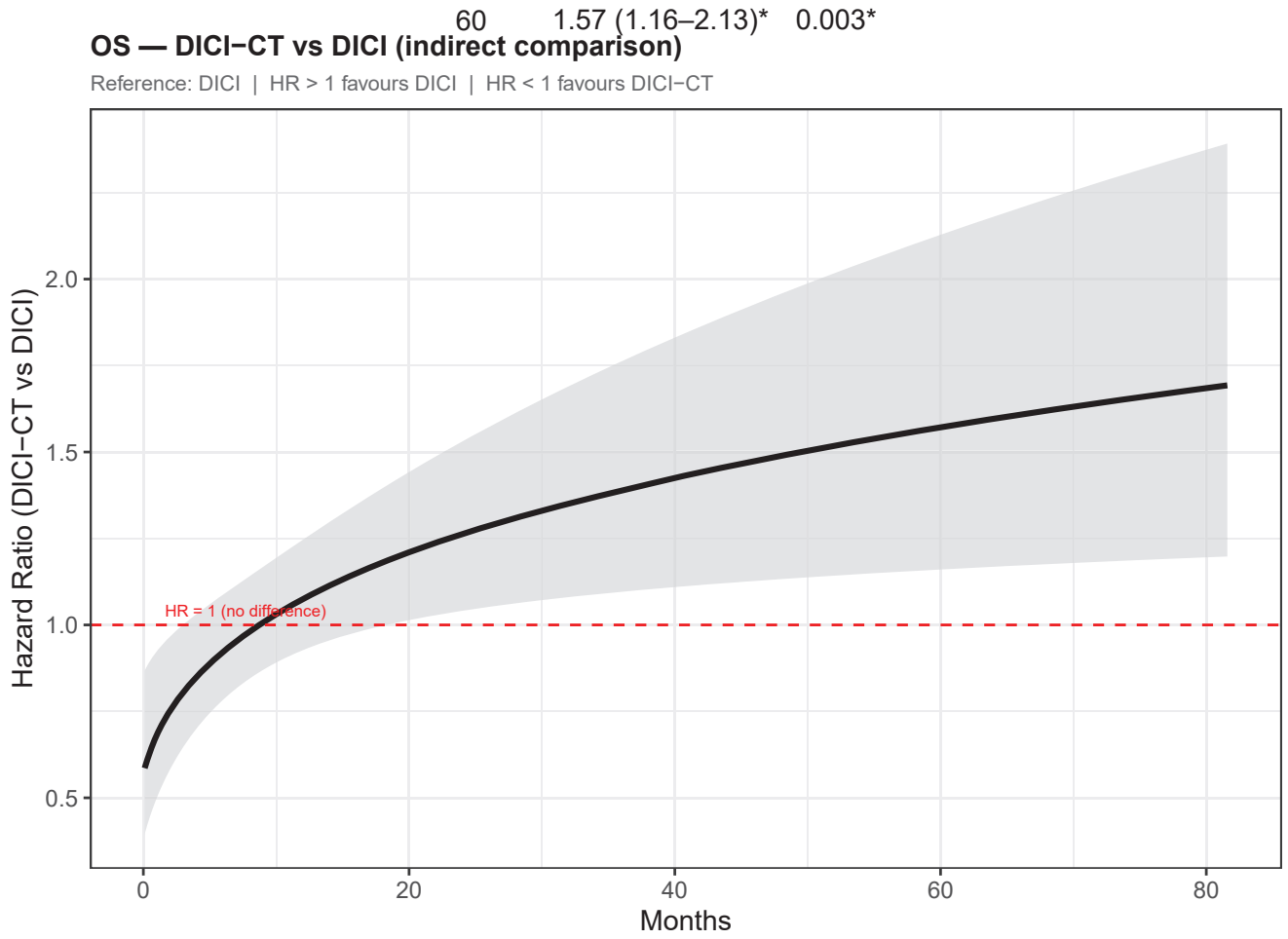

D) OS Nonsquamous

Time (mo)	HR(t) (95% CI)	P−value
0	0.59 (0.38–0.93)*	0.022*
12	1.15 (0.98–1.34)	0.086
24	1.36 (1.11–1.66)*	0.003*
36	1.50 (1.17–1.93)*	0.002*
48	1.61 (1.21–2.16)*	0.001*
60	1.71 (1.23–2.36)*	0.001*

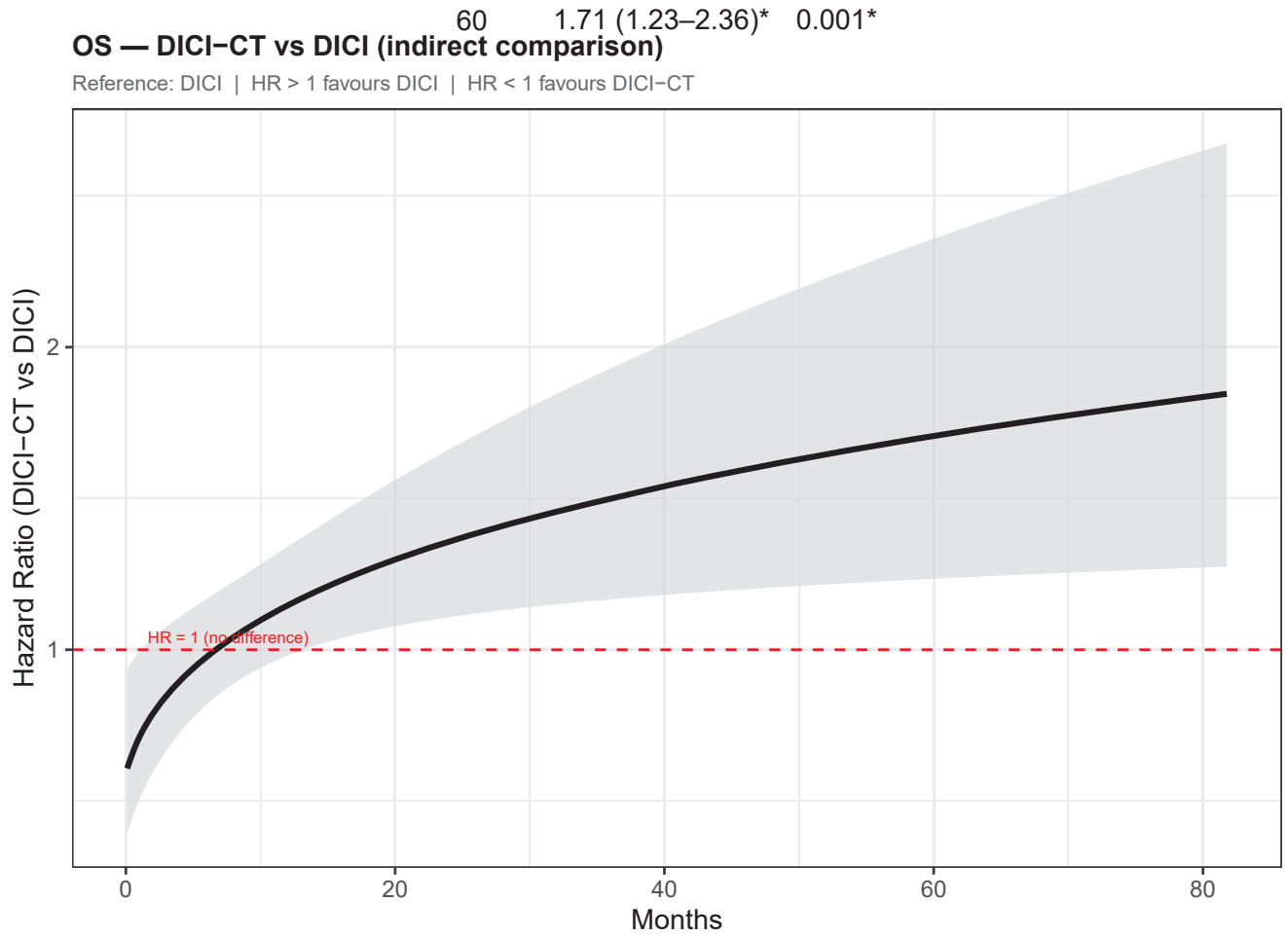

E) OS STK11 mutated

Time (mo)	HR(t) (95% CI)	P−value
0	0.14 (0.05–0.39)*	<0.001*
12	0.92 (0.52–1.65)	0.783
24	1.50 (0.65–3.48)	0.346
36	2.01 (0.72–5.58)	0.182
48	2.47 (0.78–7.86)	0.124
60	2.91 (0.82–10.29)	0.097

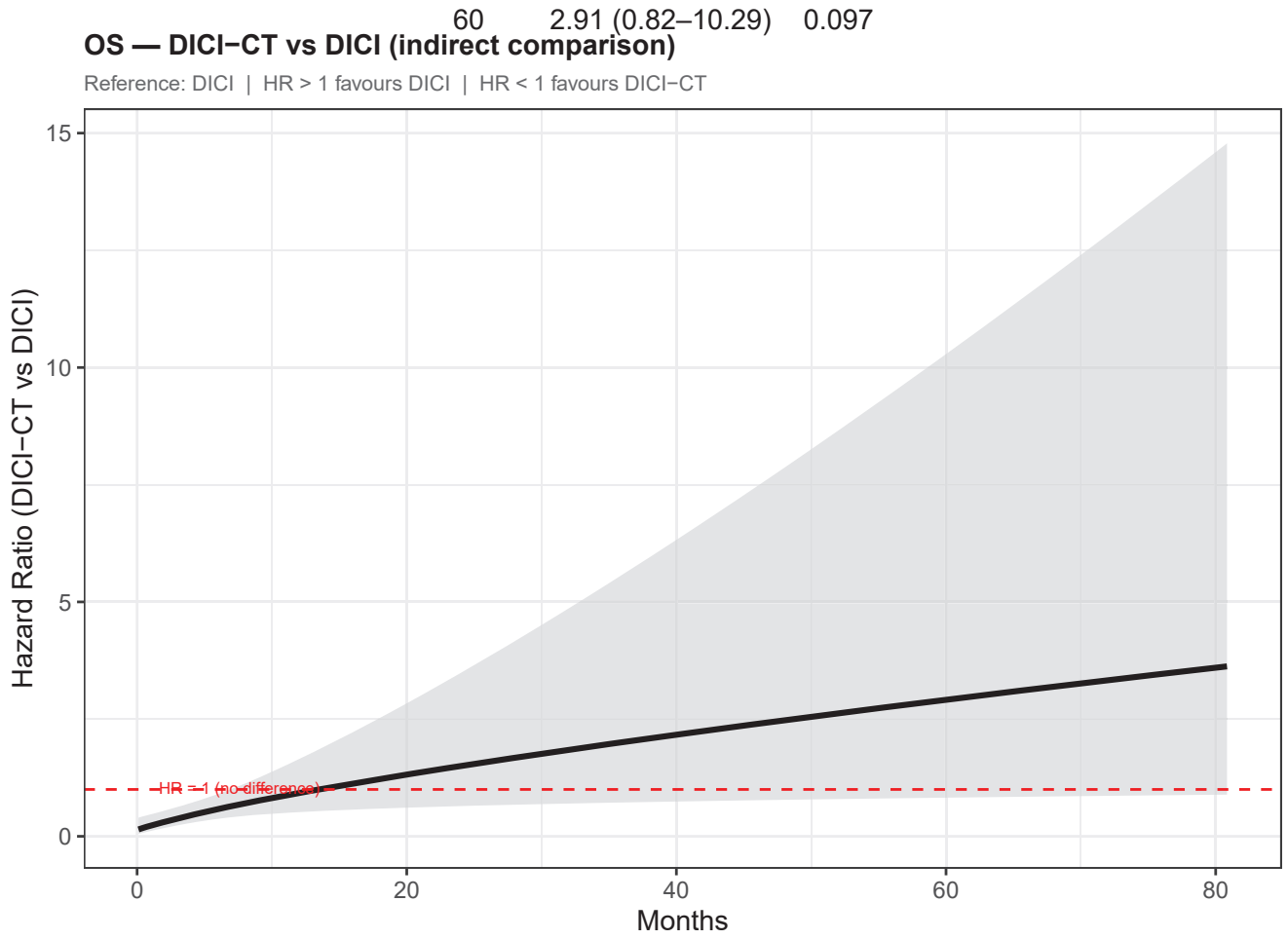

Supplementary Figure 4. Schoenfeld residuals plots: OS (KEAP1 mutated and wild-type; KTK11 mutated and wild-type patients)

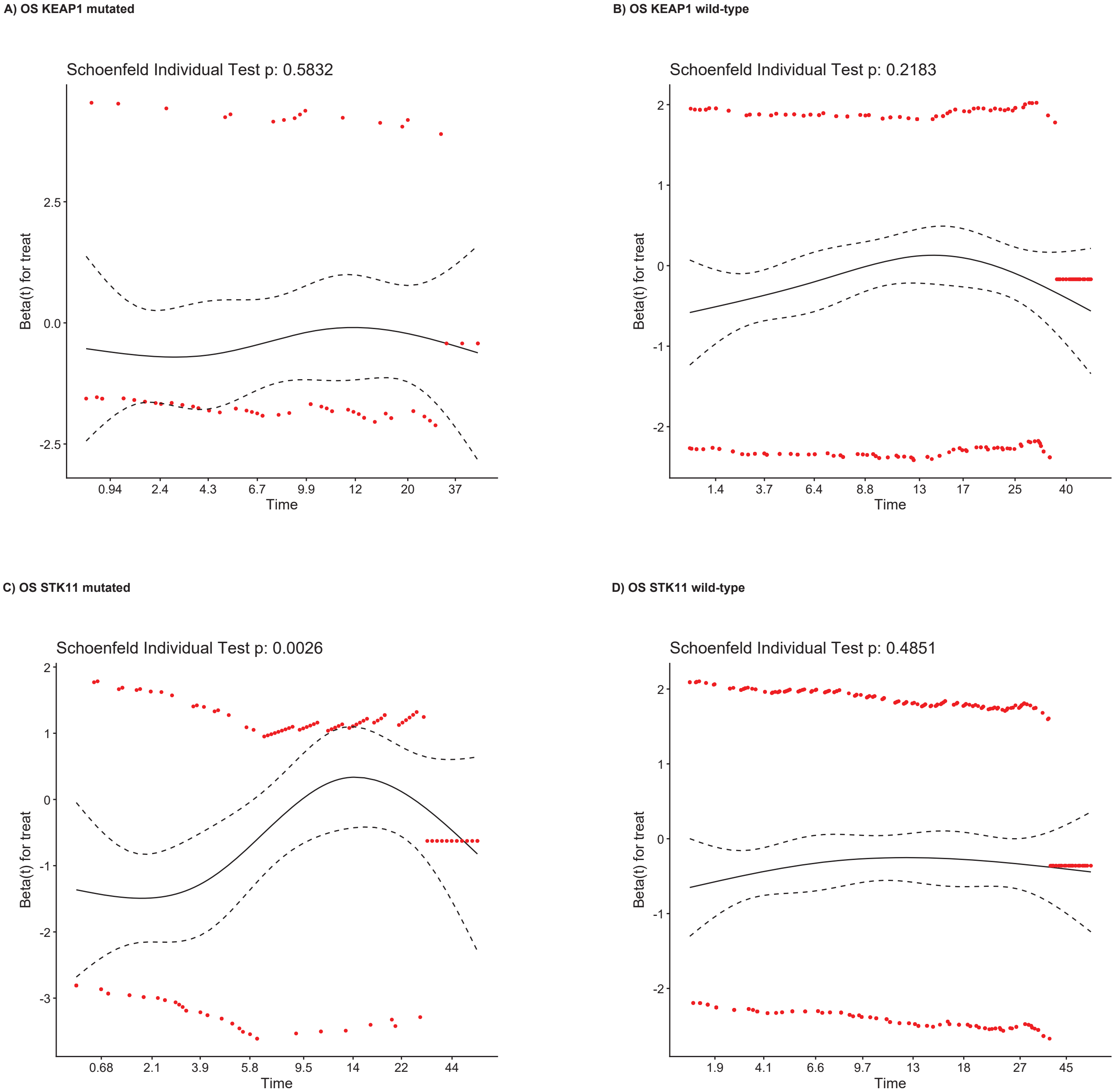

Supplementary Figure 5. Restricted Mean Survival Time plots

A) OS all patients

Restricted Mean Survival Time — OS

Shaded area = RMST up to t = 60 months

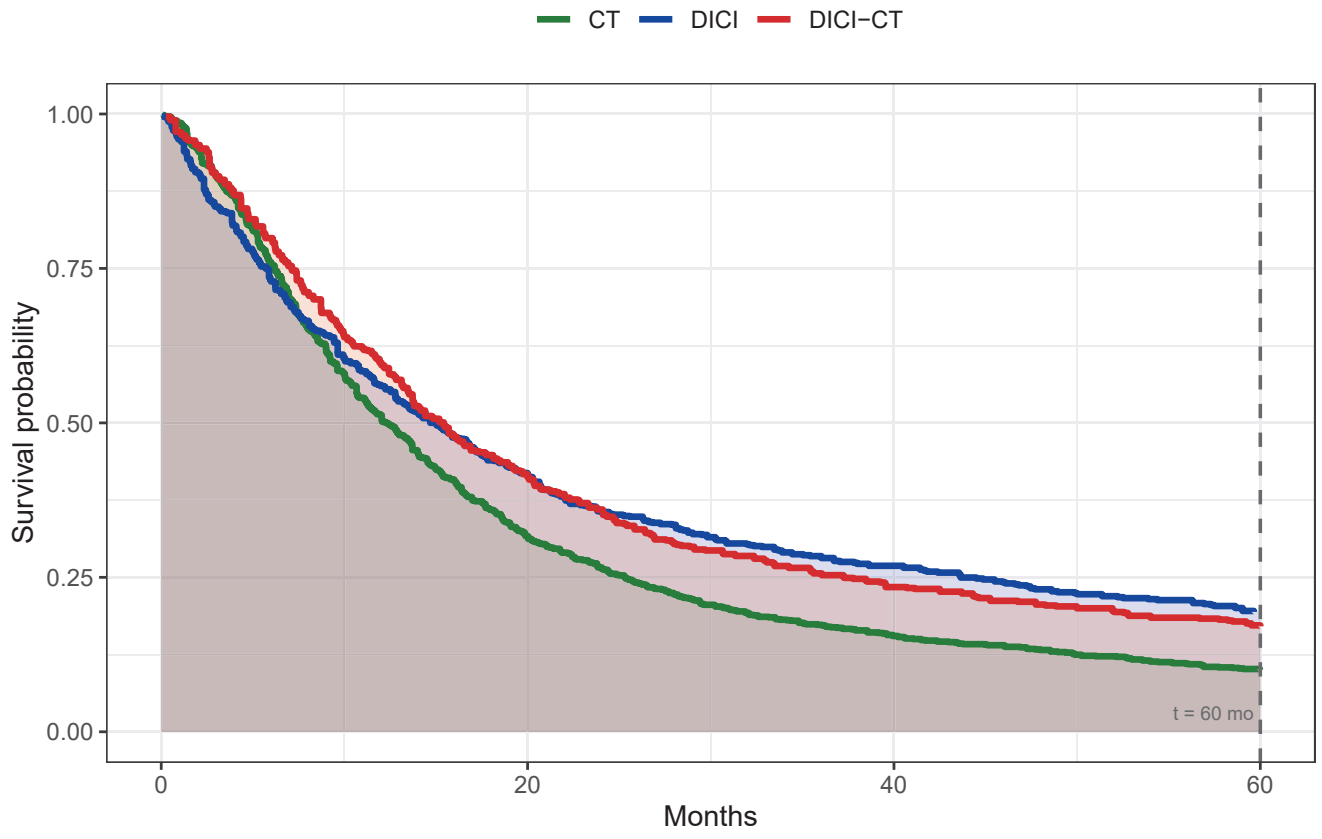

Arm	RMST (mo)	95% CI
CT	4.65	4.65 (2.96–6.33)
DICI	1.24	1.24 (1.15–1.34)
DICI−CT	1.23	1.23 (1.13–1.33)

Comparison	Diff RMST (mo)	P−value
DICI vs CT	0.89 (0.85–0.93)	0.000
DICI−CT vs CT	0.89 (0.85–0.94)	0.000
DICI vs DICI−CT (indirect)	−0.01 (−0.07–0.05)	0.815

B) PFS all patients

Restricted Mean Survival Time — PFS

Shaded area = RMST up to t = 60 months

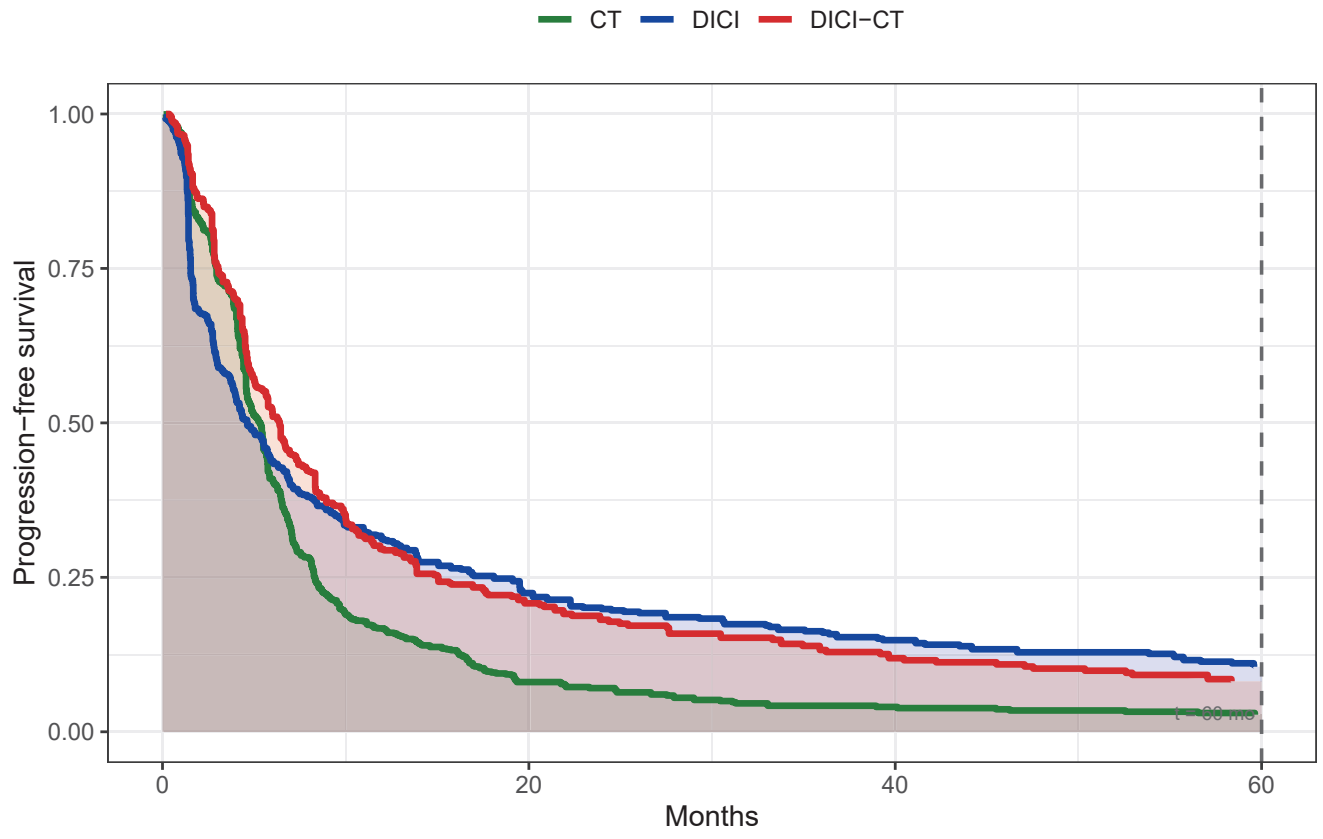

Arm	RMST (mo)	95% CI
CT	5.81	5.81 (4.06–7.56)
DICI	1.67	1.67 (1.45–1.92)
DICI−CT	1.63	1.63 (1.42–1.87)

Comparison	Diff RMST (mo)	P−value
DICI vs CT	0.89 (0.85–0.92)	0.000
DICI−CT vs CT	0.89 (0.86–0.93)	0.000
DICI vs DICI−CT (indirect)	−0.01 (−0.05–0.04)	0.764

C) OS PD-L1 ≥ 1%

Restricted Mean Survival Time — OS

Shaded area = RMST up to t = 60 months

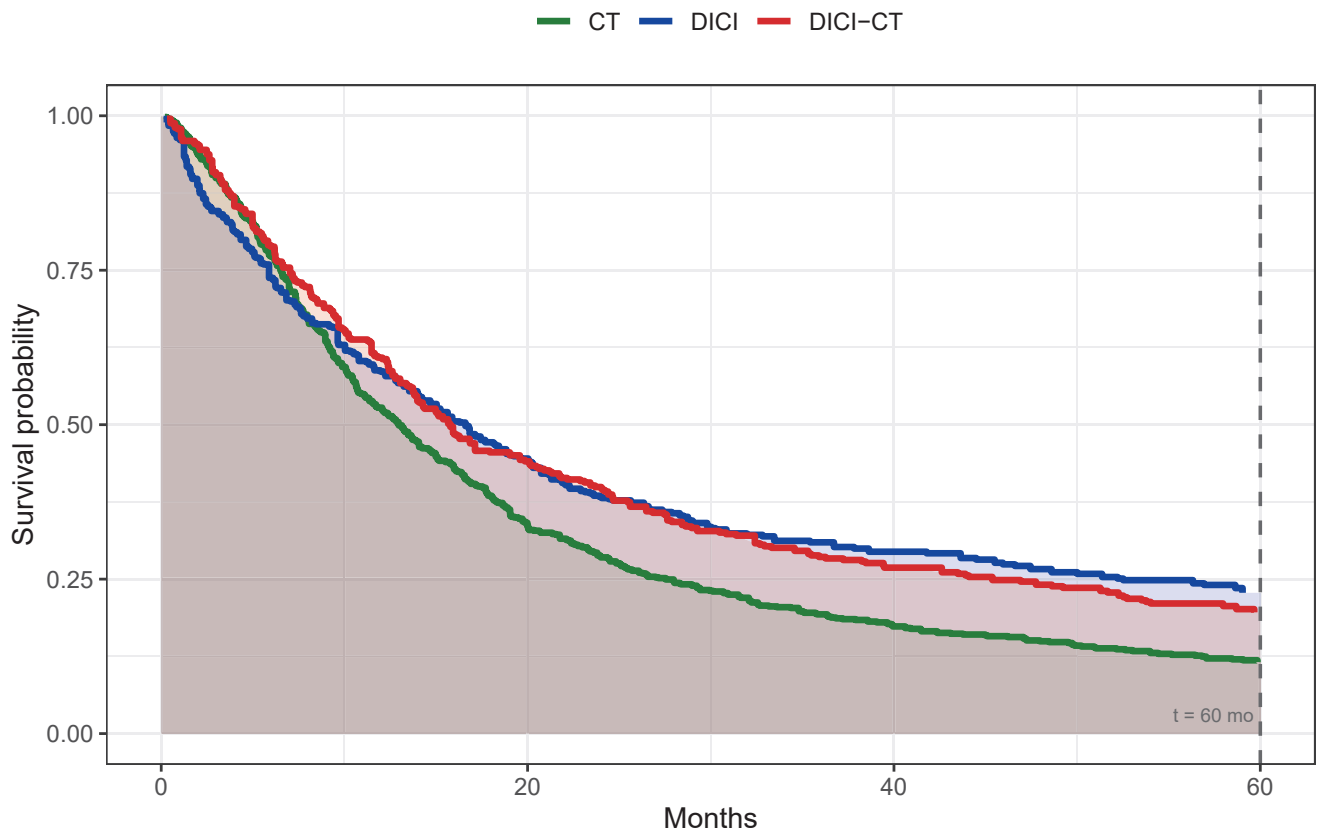

Arm	RMST (mo)	95% CI
CT	5.02	5.02 (2.75–7.30)
DICI	1.25	1.25 (1.13–1.37)
DICI−CT	1.23	1.23 (1.11–1.37)

Comparison	Diff RMST (mo)	P−value
DICI vs CT	0.87 (0.82–0.93)	0.000
DICI−CT vs CT	0.88 (0.82–0.94)	0.000
DICI vs DICI−CT (indirect)	−0.01 (−0.09–0.07)	0.855

D) OS Nonsquamous

Restricted Mean Survival Time — OS

Shaded area = RMST up to t = 60 months

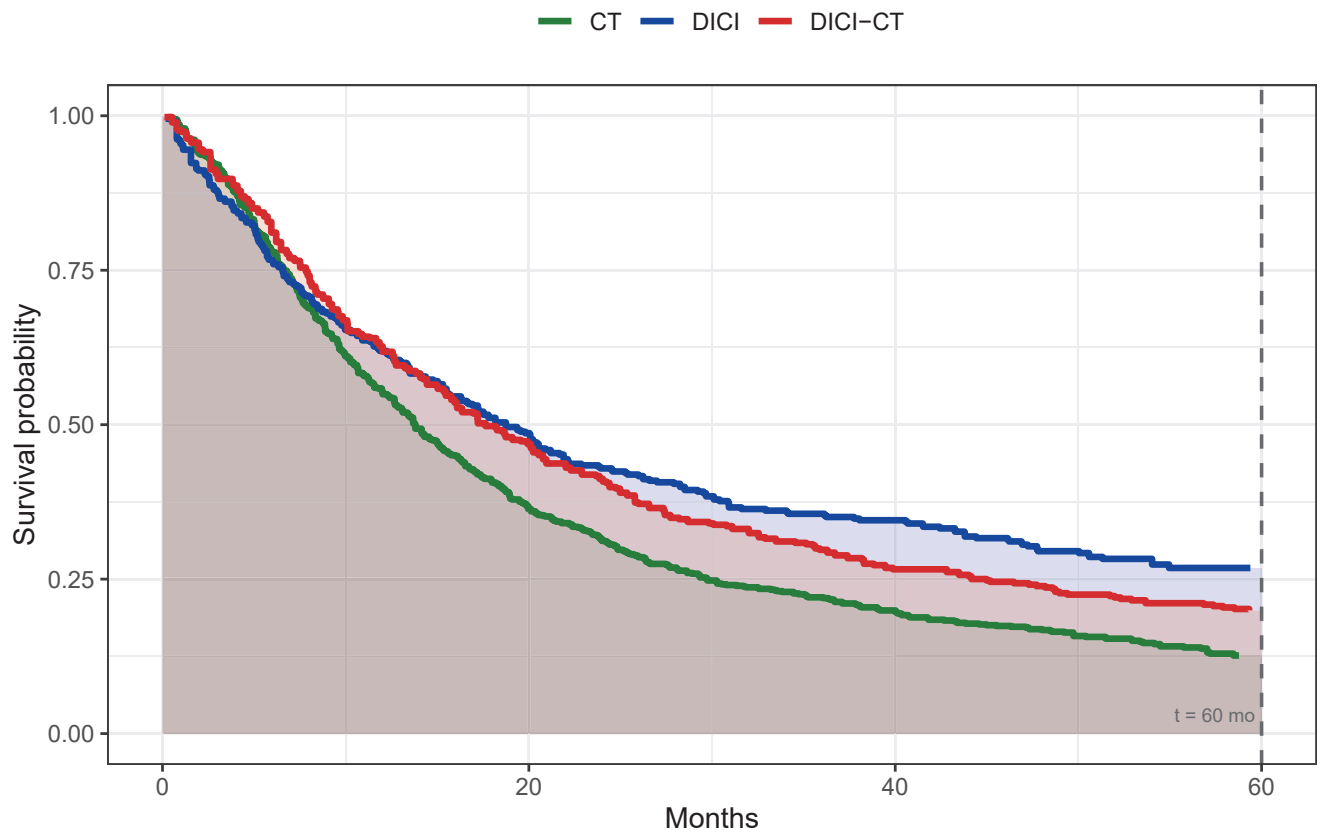

Arm	RMST (mo)	95% CI
CT	6.10	6.10 (3.52–8.69)
DICI	1.28	1.28 (1.16–1.42)
DICI−CT	1.20	1.20 (1.08–1.32)

Comparison	Diff RMST (mo)	P−value
DICI vs CT	0.84 (0.78–0.91)	0.000
DICI−CT vs CT	0.89 (0.83–0.95)	0.001
DICI vs DICI−CT (indirect)	−0.05 (−0.14–0.04)	0.269

E) OS STK11 mutated

Restricted Mean Survival Time — OS

Shaded area = RMST up to t = 24 months

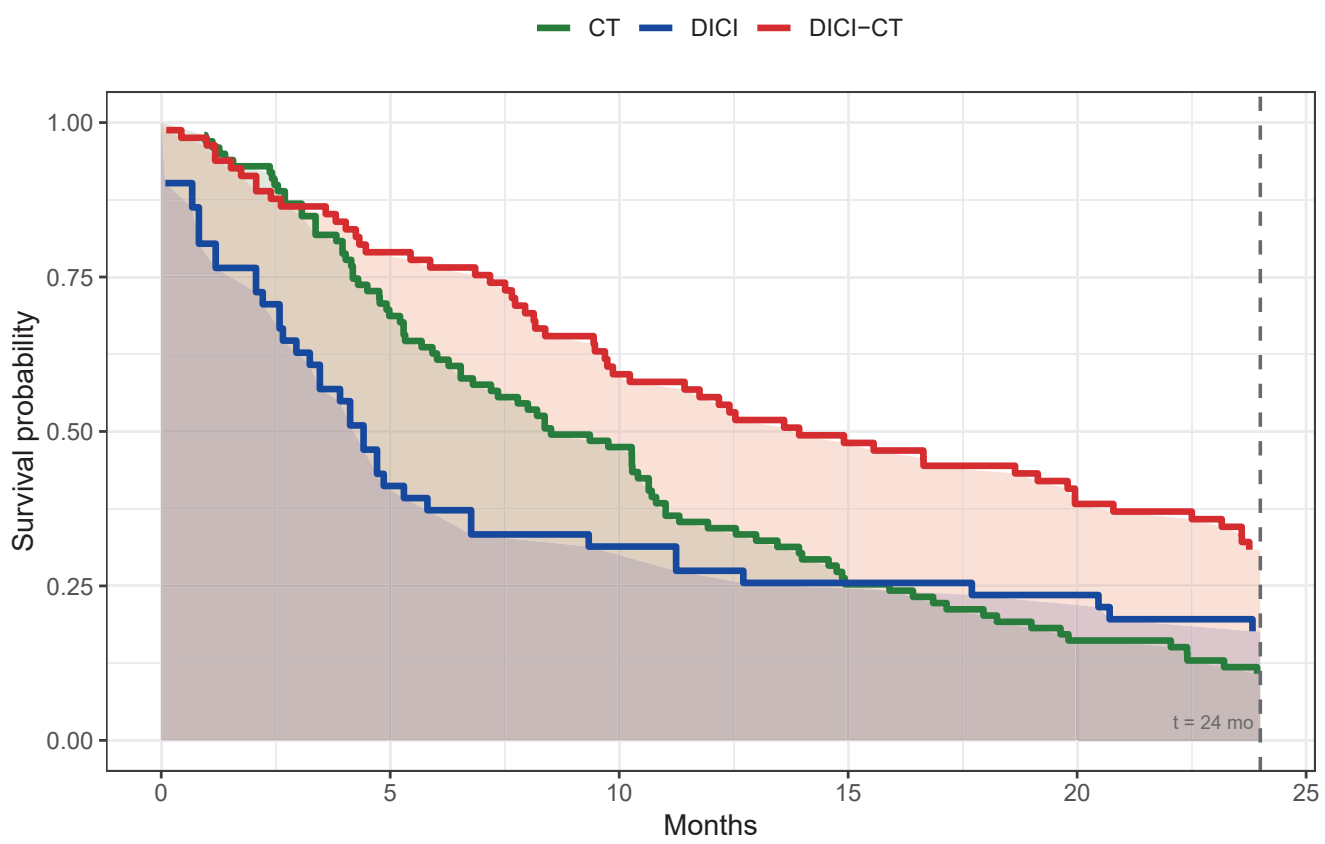

Arm	RMST (mo)	95% CI
CT	−2.02	−2.02 (−4.87–0.83)
DICI	0.81	0.81 (0.59–1.11)
DICI−CT	1.36	1.36 (1.12–1.64)

Comparison	Diff RMST (mo)	P−value
DICI vs CT	1.15 (0.95–1.39)	0.153
DICI−CT vs CT	0.72 (0.57–0.90)	0.004
DICI vs DICI−CT (indirect)	0.43 (0.16–0.71)	0.002

Supplementary Figure 6 - Traffic light plot of RoB assessment

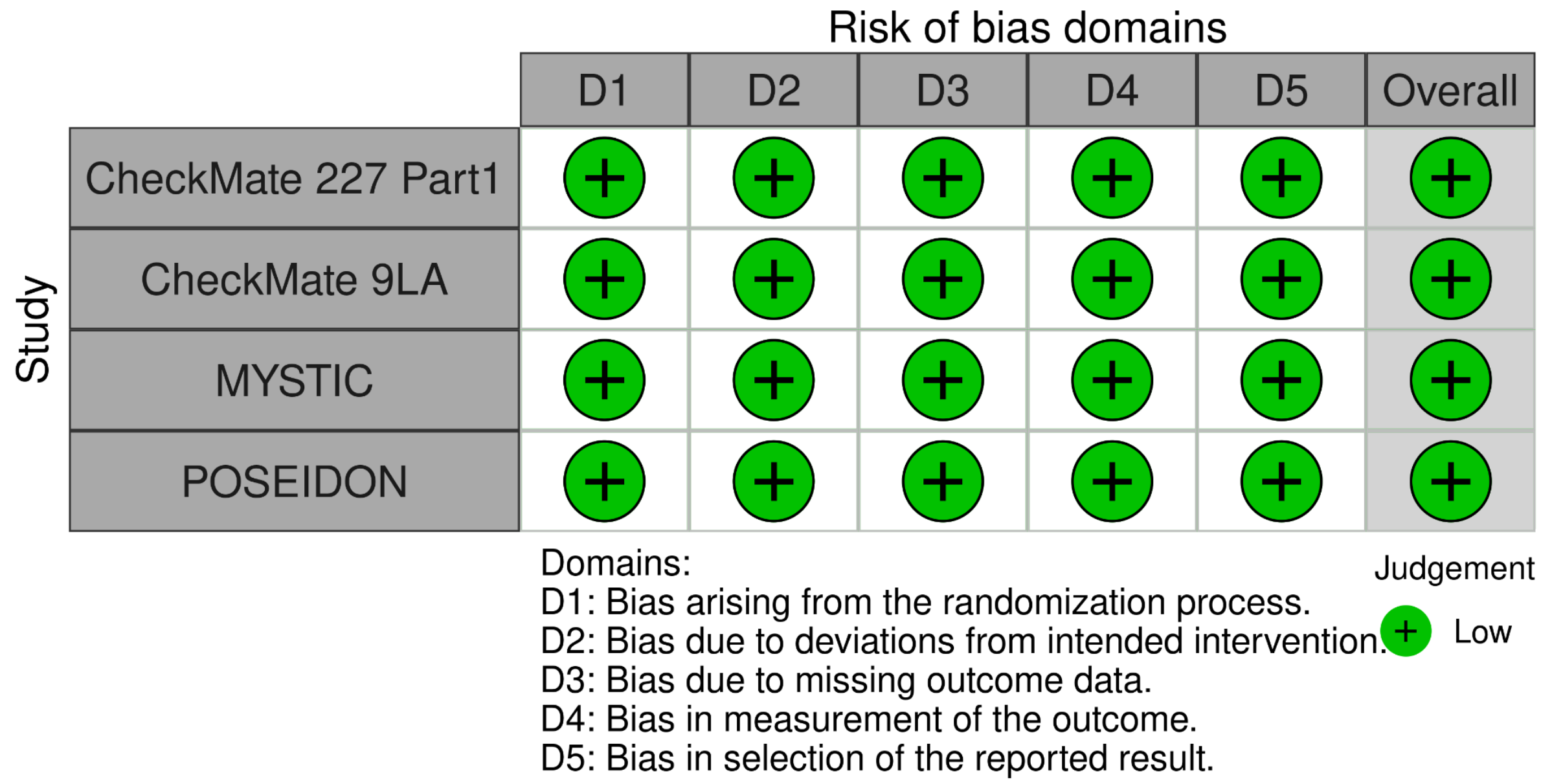

Supplementary Figure 7 - Summary plot of RoB assessment

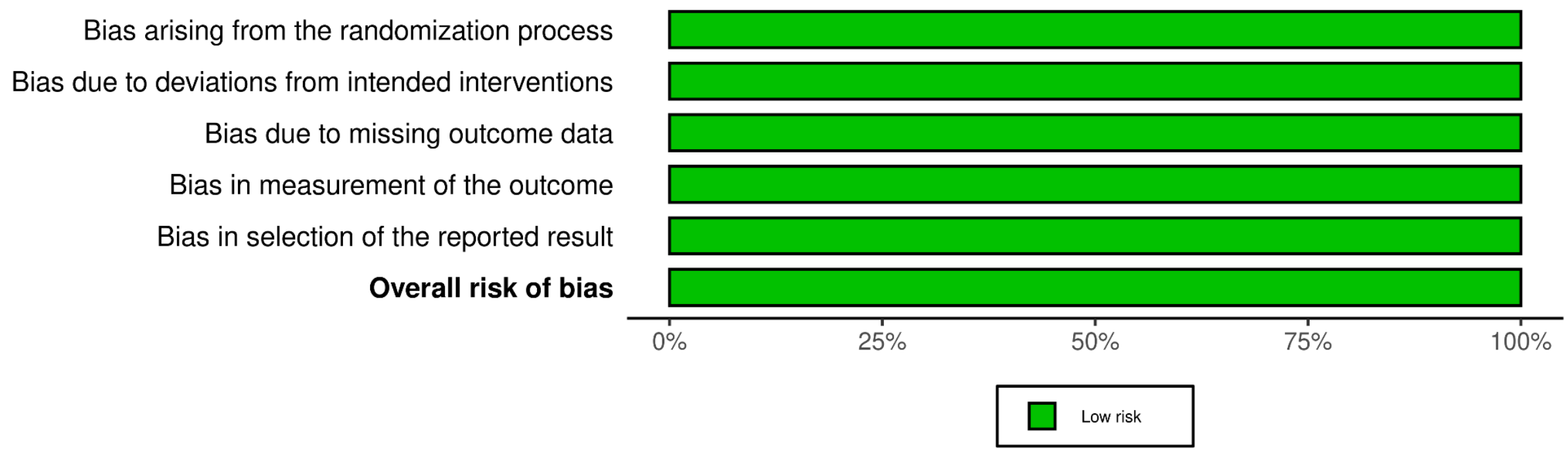

Supplement: Supplementary file 1 [file mmc1.pdf]
